# Removal of Matrix Interferences by Nano-MgO and Co-Adsorbents for Accurate Multi-Pesticide Residue Analysis in the Chinese Medicinal Herb, *Paeoniae Radix Alba*

**DOI:** 10.1155/2021/6626257

**Published:** 2021-02-04

**Authors:** Chunyu Wang, Xinquan Wang, Jiao Wang, Shanshan Di, Zhiwei Wang, Hao Xu, Huiyu Zhao, Changshan Zhao, Peipei Qi

**Affiliations:** ^1^College of Agriculture, Northeast Agricultural University, Harbin 150030, China; ^2^State Key Laboratory for Managing Biotic and Chemical Threats to the Quality and Safety of Agro-products, Institute of Quality and Standard of Agro-products, Zhejiang Academy of Agricultural Sciences, Hangzhou 310021, China; ^3^Agricultural Ministry Key Laboratory for Pesticide Residue Detection, Hangzhou 310021, China; ^4^Key Laboratory of Detection for Pesticide Residue and Control of Zhejiang, Hangzhou 310021, China

## Abstract

A simple, accurate, and high-throughput analytical method was developed to detect 123 pesticide residues in Chinese medicinal herb *Paeoniae Radix Alba* (PRA) by introducing nano-MgO as a highly efficient purification material based on quick, easy, cheap, effective, rugged, and safe (QuEChERS) design concept. Various PRA samples were extracted using 8 mL 0.5% acetic acid-acetonitrile solution and purified by a dispersive solid-phase extraction method with 30 mg nano-MgO, 40 mg primary secondary amine (PSA), and 40 mg octadecylsilane (C18) as the cleanup adsorbents, followed by liquid chromatography-tandem mass spectrometry (LC-MS/MS). 70.7% of pesticides showed a weak matrix effect after the purification process, indicating that this method can give the precise quantitative analysis of trace pesticides residue. The method was systematically validated under optimal conditions in five different kinds of PRA samples; good linearity was observed in the concentration range of 0.5–250 *μ*g/L or 1–250 *μ*g/L. Pesticide recovery in each sample spiked at concentrations of 20, 50, and 200 *μ*g/kg ranged from 98.0% to 111% and the mean relative standard deviation ranged from 2.72% to 5.70%. Furthermore, the method comparison with the traditional QuEChERS method suggested the feasibility, advantages, and potential application prospect of the present method for the multi-pesticide residue analysis in various PRA samples.

## 1. Introduction

The medicinal herb is playing an increasingly important role in our daily life, as a therapeutic drug, a dietary supplement, or a natural cosmetics additive [1, 2]. Traditional Chinese medicinal (TCM) herbs have been used to cure diseases for more than 2000 years in China [3], and 8980 medicinal herbs were recorded in the classic book *Chinese Materia Medica*. *Paeoniae Radix Alba* (PRA) is the dried root of *Paeonia lactiflora* Pall; the family is Ranunculaceae. As one of the most commonly used TCM, PRA has displayed multiple pharmacological functions, such as easing pain, nourishing blood, and treating depressive disorder [4–6]. It has also been used in cosmetics as an anti-aging ingredient in recent decades. Hence, with the extended application areas and the increasing demands, the artificial cultivation area of PRA is gradually expanding. During its cultivation, the use of pesticides is inevitable to avoid the infestation of disease, insect, and grass and consequently increase productivity [7]. Until now, China is the main producer and exporter of PRA. However, there is no pesticide registered on PRA in China; the abuse of pesticides has become a universal phenomenon, leading to serious pesticide residues in PRA, such as organophosphorus pesticides (e.g., dimethoate, chlorpyrifos, disulfoton, terbufos, and malathion) [8, 9], organochlorine pesticides [10, 11], and carbamates pesticides (e.g., carbendazim) [2]. Therefore, a reliable, simple, and high-throughput detection method is urgently needed to monitor the pesticide residue in PRA and ensure its product safety.

PRA contains many different types of ingredients, such as paeoniflorin, benzoic acid, volatile oils, and fatty oils, which makes it owning multiple functions [4]. However, the extraction of target analytes is usually accompanied by the co-extraction of these complex components, making an accurate analysis of trace pesticide residues difficult. Currently, traditional solid-phase extraction (SPE) [11], hollow fiber-based liquid-phase microextraction (HF-LPME) [12], and dispersive solid-phase extraction (dSPE) [13] have been used for pesticide residue analysis in PRA. Relatively, SPE and HF-LPME are cumbersome to operate. dSPE is more effective in purifying the sample. Solvent extraction coupled with dSPE purification was a typical quick, easy, cheap, effective, rugged, and safe (QuEChERS) method, which has become a widely accepted design concept for pesticide residue analysis in various matrices [14–17]. Currently, several studies focused on the analysis of pesticide residue in PRA based on the QuEChERS method. However, the complexity of the PRA matrix made the low pesticide coverage of these developed methods and the poor applicability for different kinds of PRA. Therefore, new efficient purification material was urgently needed to remove the matrix interferences and improve the method accuracy, efficiency, and applicability.

Nano-MgO has been reported as an excellent adsorbent to adsorb chemical pollutants such as humic acid [18] and phosphate [19] from water samples, likely due to its amphiphilic character. According to the Lewis acid-base theory, Mg^2+^ at Lewis acid sites can interact with alkaline substances, while O^2−^ at Lewis basic sites can adsorb acidic substances [20, 21]. This characteristic lets us consider the feasibility of nano-MgO in the removal of the acid active compounds in the PRA matrix. Meanwhile, as a nanooxidation material, its small particle size and large specific surface area can help to enhance its adsorption performance. Besides, owing to the diversity of the active compounds in the PRA matrix, the coadsorbents were beneficial to improve the purification effect and commercial C18 and PSA can eliminate the interference of oils and weak polar compounds, respectively.

The present work was aimed at establishing a rapid method for the detection of 123 pesticide residues in various PRA samples based on the selection of purification materials according to the QuEChERS design concept, followed by liquid chromatography-tandem mass spectrometry (LC-MS/MS) analysis. The 123 common pesticides that may be used in PRA cultivation were selected as target analytes, including organophosphorus, carbamate, nicotine, triazole, and amides. Both the extraction and purification steps were systematically optimized, and nano-MgO was innovatively used as a dSPE adsorbent to reply to the complexity of the PRA sample, significantly reducing the matrix effect. Moreover, the performance of the established method was validated and compared with the traditional QuEChERS method to verify its feasibility in pesticide residue analysis in PRA samples.

## 2. Experimental

### 2.1. Materials

Five different PRA samples were purchased from diverse main producing areas according to its planting situation, including Zhejiang, Anhui, Shandong, Henan, and Hebei provinces in China. The samples were named PRA-ZJ, PRA-AH, PRA-SD, PRA-HN, and PRA-HB, respectively. The samples were pulverized by a grinder and sieved through a 100-mesh sieve, and PRA powder was stored in a desiccator at room temperature until measured.

### 2.2. Chemicals and Regents

Nano-MgO was obtained from Nanjing XFNANO Materials Tech Co., Ltd. (Nanjing, China). High-performance liquid chromatography (HPLC) grade acetonitrile (ACN), methanol, and isopropanol were purchased from Merck (Darmstadt, Germany). Acetic acid (HAc) was of analytical grade and was purchased from YONGHUA Chemical Co., Ltd. (Jiangsu, China). Primary secondary amine (PSA), octadecylsilane (C18), anhydrous magnesium sulfate (MgSO_4_), and sodium chloride (NaCl) were obtained from Bonna-Agela Technologies Co. Ltd. (Tianjin, China). HPLC grade ammonium formate was purchased from Tedia (Fairfield, USA). Ultrapure water was used throughout the experiment. 123 pesticides were purchased from the Ministry of Agriculture, Agro-Environmental Protection Institute (Tianjin, China), or Shanghai Pesticide Research Institute (Shanghai, China). All pesticides were formulated as a mixed standard of 5 mg/L and stored in a refrigerator at 4°C.

### 2.3. Sample Preparation

The PRA powder (2 g) was weighed into 50 mL centrifuge tubes, followed by the addition of 10 mL of water and 8 mL of a 0.5% acetic acid-acetonitrile solution for the extraction process. The mixture was vortexed for 1 min to ensure adequate contact between the extraction solvent and the sample. Then, 6 g of anhydrous MgSO_4_ and 1.5 g of NaCl were added to the tube and vortexed for 1 min. The resulting mixture was centrifuged at 7000 g for 5 min using a Thermo Scientific Biofuge Primo *R* centrifuge (Germany).

**Figure 1:**
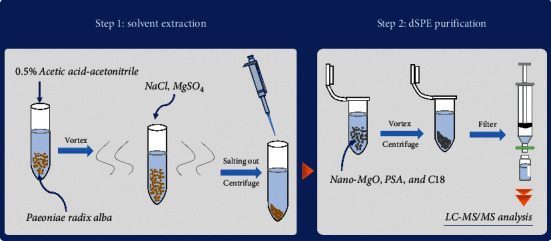
The scheme of the established pretreatment method procedure.

The supernatant acetonitrile (1 mL) in the extraction solution was transferred into 2 mL centrifuge tubes containing 30 mg of nano-MgO, 40 mg of PSA, 40 mg of C18, and 150 mg of anhydrous MgSO_4_. After being vortexed for 1 min, the resulting mixture was centrifuged at 7000 g for 5 min. Later, 600 *μ*L of supernatant was mixed with 150 *μ*L of water. The solution was filtered through 0.22 *μ*m filters before the LC-MS/MS analysis. The scheme of the established pretreatment method procedure is shown in Figure 1.

During the method optimization, PRA-ZJ was used as a typical sample and it was spiked with 123 pesticides at 0.2 mg/kg. First, the types and volume of extraction solvents were optimized for sample solution preparation. The extraction solvent was directly analyzed by LC-MS/MS without purification. Subsequently, the amounts of dSPE adsorbents, nano-MgO, PSA, and C18, were successively optimized. The extraction solution (1 mL) of the prespiked sample was transferred to a centrifuge tube containing different amounts of nano-MgO to explore the best purification effect. The amounts of PSA and C18 were sequentially tested based on the presence of nano-MgO. The recovery and matrix effect were used as the main parameters to determine the type and volume of extraction solvent and the optimal cleanup adsorbent dosage.

### 2.4. LC-MS/MS Analysis

The detection of 123 pesticides in the PRA samples via LC-MS/MS was carried out on a Nexera X2 LC system (Shimadzu, Kyoto, Japan) and Shimadzu LCMS-8050 with an electrospray ion source (ESI). An analytical column, ACE Excel 2 C18 (100 mm × 2.1 mm, 1.7 *μ*m; Phenomenex, United Kingdom), was used for the chromatographic separation with a column temperature of 35°C. The mobile phase consisted of 5 mmol/L ammonium formate aqueous solution (phase A) and methanol (phase B) with a gradient elution procedure. The gradient elution program is shown in Table S1. The total run time was 10 min and the flow rate was set at 0.3 mL/min, while the injection volume was 2 *μ*L. The multiple reaction monitoring (MRM) mode was adopted for tandem spectrometry. The optimal collision energy and MRM precursor ion, product ion, and declustering potential for all target analytes are shown in Table S2. The retention time of each pesticide was 2 ms. The electrospray ionization source was used with positive and negative ion modes. The temperature of the ESI capillary, heart block, and DL was 300°C, 400°C, and 250°C, respectively. Both the heating and drying gas flow were set at 10 L/min, while the flow rate of the nebulizing gas was set at 3 L/min.

### 2.5. Method Validation

The calibration curves of the analytes were fabricated by plotting the peak area as a function of the concentration and were used to evaluate the linearity of the pesticides. Both matrix-matched and solvent standard solutions were prepared with the concentration of each analyte at 0.5, 1, 2, 5, 10, 20, 50, 100, 200, and 250 *μ*g/L. The recovery experiments were implemented by spiking five PRA samples with 123 pesticides at three different concentration levels (20, 50, and 200 *μ*g/kg). The accuracy was evaluated with 3 replicates for each spiked concentration level.

## 3. Results and Discussion

The recovery parameter was used to evaluate the extraction and purification efficiency, which is the ratio of the detected concentration to the spiked concentration [22]. The distribution of pesticide recovery was statistically divided and evaluated due to the presence of numerous pesticides. In the multi-pesticide residue analysis, a recovery range of 70–130% was required for accurate analysis, while 90–110% was more satisfactory.

### 3.1. Selection and Volume of the Extraction Solvent

**Figure 2:**
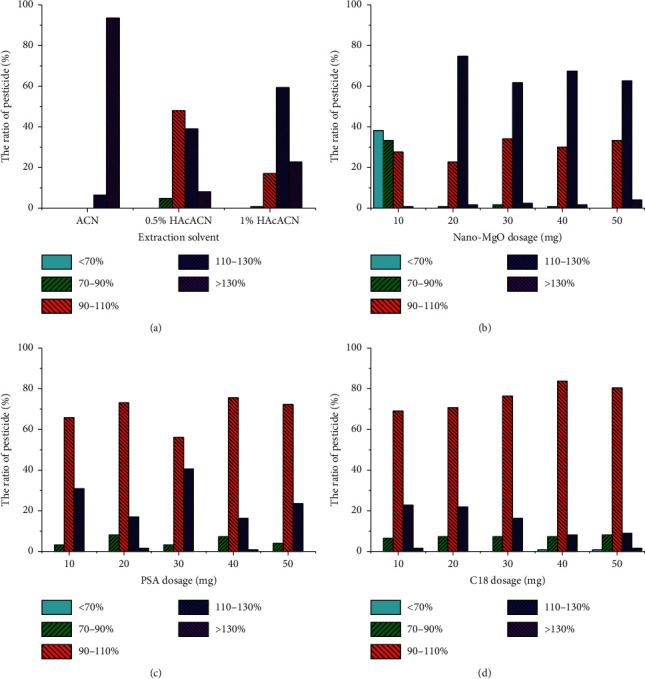
Influence of the extraction solvent (a), nano-MgO dosage (b), PSA dosage (c), and C18 dosage (d) on the pesticide recovery distribution.

The extraction solvent was selected to ensure efficient extraction of the target analyte and reduction of the interference of the coextracts. Three types of extraction solvents were compared, including pure acetonitrile, acetonitrile containing 0.5% acetic acid, and acetonitrile containing 1% acetic acid. The volume of extraction solvents was 8 mL. The recovery distribution of the pesticides extracted by different solvents is shown in Figure 2(a). When acetonitrile was used as the extraction solvent, pesticide recovery was higher than 110%, with 93.5% of pesticides with rates higher than 130%. When acetic acid was added to acetonitrile, the rate of the analytes in the matrix was distinctly improved. When acetonitrile containing 0.5% acetic acid was used, 90.0% of the pesticides were recovered in the range from 75.7% to 128%, with 48.0% of the pesticides with recoveries of 90–110%. When the content of acetic acid in acetonitrile was increased to 1%, 77.2% of the pesticides were recovered with a range between 70% and 130%, and only 17.1% of the pesticides were in the recovery range of 90–110%. Hence, acetonitrile containing 0.5% acetic acid was used as the extraction solvent.

After the extraction solvent was determined, the solvent volume was optimized. The spiked PRA samples were extracted with 6, 8, and 10 mL of acetonitrile containing 0.5% acetic acid. The recovery distribution of these pesticides was then calculated. The results (Figure S1) showed that when different volumes of extraction solvent were used, 71.5% (6 mL), 78.1% (8 mL), and 76, 4% (10 mL) of pesticides were in the recovery range of 90–110%, respectively. It has been illustrated that 8 mL extraction solvent was better than 6 mL and 10 mL. Hence, 8 mL acetonitrile containing 0.5% acetic acid was used in the further experiment.

### 3.2. Optimization of the Purification Materials

Nano-MgO was expected as a purification material to remove these acid compounds, such as benzoic acid and volatile oils in the PRA matrix. The nano-MgO dosage (10, 20, 30, 40, and 50 mg) was firstly optimized as the abovementioned method in section 2.3. The recoveries obtained with different amounts of nano-MgO are shown in Figure 2(b). It can be seen that when the dosage was 10 mg, the recovery of all pesticides was less than 110%, with 38.2% of pesticides presenting recovery rates lower than 70%. Recovery increased with increasing MgO content, specifically with more than 95.9% of pesticides observed in the recovery range of 70–130%. When the amount of nano-MgO increased to 30 mg, 34.2% of pesticides were in the recovery range of 90–110%, which was maintained with increasing nano-MgO content. Under the premise of comprehensive consideration of recovery efficiency and MgO dosage, 30 mg was chosen as the optimum nano-MgO content.

PSA is further employed as coadsorbent due to its characteristics as a weak anion exchanger, which can be used to remove oil, fatty acids, and waxy interferences. The combination of PSA and nano-MgO was expected to effectively remove a wide variety of impurities. The various amounts of PSA (10, 20, 30, 40, and 50 mg) were studied with the presence of 30 mg nano-MgO. The experimental operation was performed according to the procedures described previously and the recovery rate distribution results are shown in Figure 2(c). When the dosage of PSA was 40 mg, 75.6% of pesticides were in the recovery range of 90–110%, which was slightly higher than the other four dosages. Compared with the purification without PSA, the percentage of pesticides in the recovery range of 90–110% increased from 34.2% to 75.6%. This fully demonstrates that additional components were removed by adding PSA, and consequently, the recovery rates were improved.

C18 is reported to be useful in removing lipids and non-polar interferences in the solid-phase extraction, so the component was added to the purification process. The amounts of C18 were set as 10, 20, 30, 40, and 50 mg, respectively. It can be clearly seen from Figure 2(d) that the pesticide recovery rate distribution was significantly better when 40 mg of C18 was used. The proportion of pesticides with recovery rates in the range of 90–110% was as high as 83.7%. Compared with the purification with nano-MgO and PSA alone, the proportion of pesticides in the range of 90–110% increased by 8.10%. Hence, the ultimate purification materials in this method were nano-MgO (30 mg), PSA (40 mg), and C18 (40 mg).

### 3.3. Purification Effect Evaluation

The matrix effect has been an inevitable problem in pesticide residue analysis based on LC-MS/MS [23], making quantitative analysis inaccurate. Hence, the matrix effect was used to evaluate the purification effect in the developed method. Typically, the evaluation of the matrix effect can be performed through two different methods. The first one is based on the peak area ratio (*R*_a_) of the matrix-matched standard solution to the solvent standard solution at a certain concentration. The second one is based on the ratio of the slope of the matrix-matched standard solution curve to that of the solvent standard solution curve. The results obtained by the two different methods usually are in good agreement. Here, the peak area ratio was used in the optimization process. In general, a result in which *R*_a_ is equal to 1 means no matrix effect, and an *R*_a_ value within 0.8–1.2 is considered an acceptable weak matrix effect [24, 25]. The *R*_a_ values were divided into five groups to evaluate the matrix effect: 0–0.2, 0.2–0.5, 0.5–0.8, 0.8–1.2, and >1.2. *R*_a_ values within 0.8–1.2 represented a weak matrix effect as mentioned before, and it is acceptable for an accurate analysis, while *R*_a_ values lower than 0.8 displayed signal suppression. Specifically, the smaller the *R*_a_ value, the stronger the signal suppression. Moreover, *R*_a_ values greater than 1.2 displayed signal enhancement.

**Figure 3:**
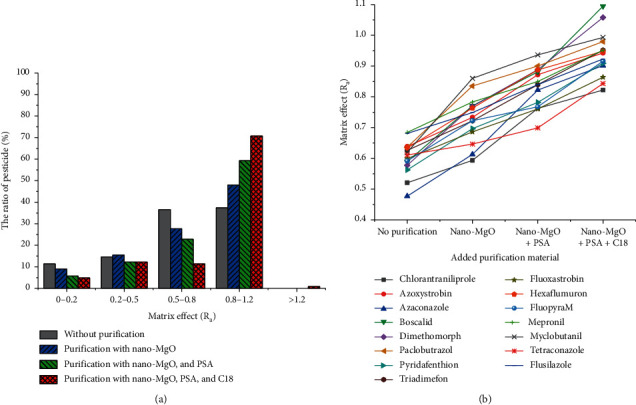
Effects of different purifications on matrix effect of pesticides (a) and fifteen representative pesticides (b).

**Figure 4:**
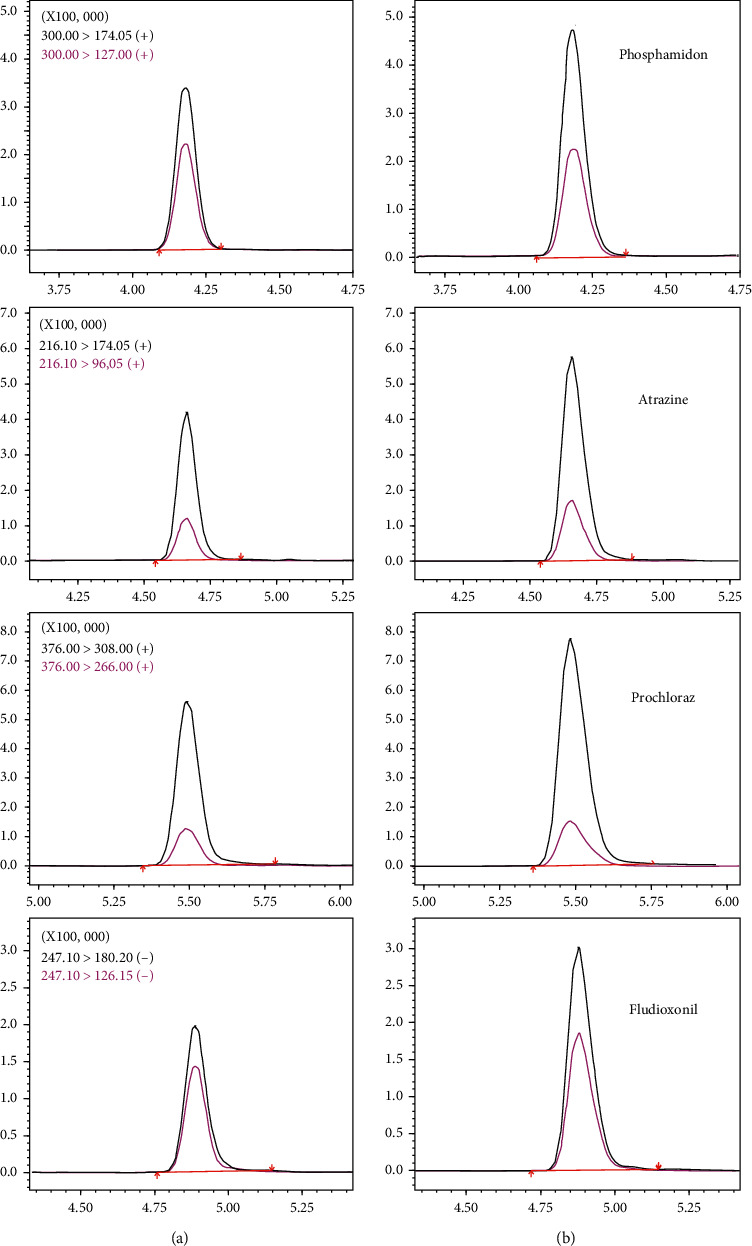
XIC chromatograms of four representative pesticides at 40 *μ*g/L in unpurified solution (a) and purified solution (b).

The distribution of *R*_a_ values obtained from the results is displayed in Figure 3. Comparing the results without purification materials (Figure 3(a)), the percentage of pesticides with weak matrix effects at the optimum dosage of 30 mg nano-MgO increased from 37.4% to 48.0%. The results indicate that the addition of nano-MgO reduced the matrix effect of some of the pesticides. When PSA was added, 59.4% of pesticides exhibited *R*_a_ values within 0.8–1.2, demonstrating the matrix effect of some of the pesticides had been further improved. With the addition of C18, more than 70.0% of pesticides showed a weak matrix effect. Additionally, the results in Figure 3(a) clearly demonstrate that the ratio of analytes with *R*_a_ values of 0.5–0.8 decreased with the gradual addition of purification materials, whereas *R*_a_ values of 0.8–1.2 increased. Moreover, it can be clearly seen from Figure 3(b) that for 15 pesticides, such as chlorantraniliprole, azoxystrobin, and azaconazole, the *R*_a_ values finally displayed weak or no matrix effect compared with the initial strong signal suppression and increasingly approached 1 in the optimization process. Most pesticides with a reduced matrix effect were triazole and amides. In particular, azaconazole, paclobutrazol, and myclobutanil showed the most significant improvement, with *R*_a_ values increased from 0.48, 0.64, and 0.62 to 0.90, 0.98, and 0.99, respectively. In addition, the signal intensity before and after purification was compared, and the XIC chromatograms of four representative analytes are provided in Figure 4. Higher peak-heights of phosphamidon, atrazine, prochloraz, and fludioxonil were obtained after purification. The above results fully illustrate the superiority of the established method in improving matrix effects.

### 3.4. Method Validation

The feasibility and adaptability of the method were validated by evaluating linearity, sensitivity, matrix effect, and precision of the pesticide residue analysis in five PRA samples: PRA-ZJ, PRA-AH, PRA-SD, PRA-HN, and PRA-HB. The linear regression equations for five different PRA samples are provided in Tables S3–S7. In sample PRA-ZJ, 83.7% of the analytes were linear in the range of 0.5–250 *μ*g/L, and the correlation coefficient (*R*) was higher than 0.99. Because of the high detection sensitivity of the instrument for some of the compounds, the upper limit of the linear range could be reduced to 50 *μ*g/L or 100 *μ*g/L for some pesticides. Additionally, 14 pesticides were within the linear range from 0.5 to 100 *μ*g/L, including malaoxon, hexazinone, phorate sulfone, azoxystrobin, and flutolanil (Tables S3–S7). The linearity range of four other pesticides (vitavax, ethiofencarb, mepronil, and phosalone) was from 0.5 to 50 *μ*g/L. Moreover, the linear range of pesticides changed slightly with different matrices. The limit of detection (LOD) was determined as three times the concentration of the signal to noise ratio of the instrument. As shown in Tables S3–S7, LODs were in the range of 0.16–4.91 *μ*g/kg for sample PRA-ZJ, 0.14–4.99 *μ*g/kg for samples PRA-AH and PRA-SD, 0.11–4.98 *μ*g/kg for PRA-HN, and 0.16–4.98 *μ*g/kg for PRA-HB.

The limit of quantification (LOQ) of the method was determined according to the European Commission guidance document SANTE/12682/2019 [26]. In the present work, the lowest spiked concentration of the pesticides was 20 *μ*g/kg. Satisfactory recoveries were achieved for 94.0% of the pesticides at this concentration, and thus the method LOQ of these pesticides was 20 *μ*g/kg. Other six pesticides, such as 3-hydroxy-carbofuran, abamectin, clothianidin, dicrotophos, monocrotophos, and terbufos, showed satisfactory recoveries at 50 *μ*g/kg. Therefore, the method LOQ of these six pesticides was determined to be 50 *μ*g/kg.

The matrix effect was evaluated by comparing the ratio of the slope of the matrix to the solvent-matched curves in the validation process [27]. The results of the matrix effect are shown in Tables S3–S7. The pesticides with a ratio from 0.8 to 1.2 for PRA-ZJ, PRA -AH, PRA-SD, PRA-HN, and PRA-HB account for 61.8%, 58.5%, 54.5%, 63.4%, and 59.4% of all pesticides, respectively. This shows that the method was applicable to five different PRA samples, and more than half of the analytes had acceptable matrix effects.

In addition, recovery experiments were used to evaluate the accuracy and precision of the method. The detailed results of the recovery and the relative standard deviation (RSD) for all pesticides in five different PRA samples at three concentration levels are shown in Tables S8–S12. The mean of the recovery rate and RSDs and the number of pesticides not detected at each spiked concentration are collated in Table 1. It was determined that the mean of the recovery rates of the five different PRA samples ranged from 98% to 111% and the mean RSD was less than 6.00%. The overall average recovery between all the PRA samples for the concentrations of 20, 50, and 200 *μ*g/kg was 103%, 106%, and 103%, respectively, with mean RSD values of 4.83%, 3.82%, and 2.87%. When the spiked concentration was 20 *μ*g/kg, the number of undetected analytes ranged from 6 to 17. When the concentration increased to 200 *μ*g/kg, all pesticides were detected. More than 78.9% of the detected pesticides had satisfactory recovery rates on the three concentration levels in the five PRA samples. The number of pesticides with recovery rates between 70% and 120% at each concentration level in each PRA sample is shown in Table 1. Based on the aforementioned results, it can be confirmed that the method has satisfactory precision.

### 3.5. Method Comparison

**Figure 5:**
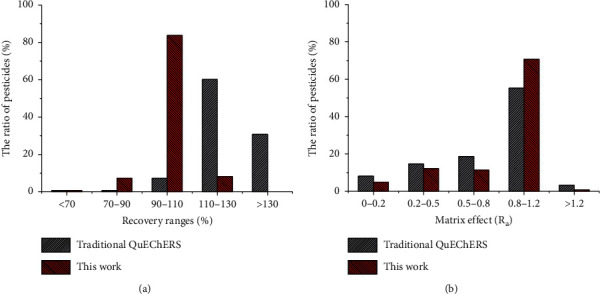
Comparison of recovery (a) and matrix effect (b) of the developed method with the traditional QuEChERS method for pesticides analysis of PRA-ZJ sample.

The performance of this method was compared with that of the traditional QuEChERS method [8], in which no acid is added in the extraction process and the extraction solution is purified by C18 and PSA. Sample PRA-ZJ, at a spiking concentration of 0.2 mg/kg, was extracted and purified by the two methods, and the results of the recovery rates and matrix effect are shown in Figure 5. The recovery rate and *R*_a_ value distribution were compared to analyze the two methods. As shown in Figure 5(a), compared with the traditional QuEChERS method, the percentage of pesticides with recovery rates in the range of 90–110% increased from 7.32% to 83.7%, demonstrating a significant improvement in the recovery of pesticides by using the present method. In terms of the matrix effect (Figure 5(b)), the proportion of pesticides with an *R*_a_ value in the range of 0.8–1.2 increased from 55.3% to 70.7%. It can be hypothesized that the matrix effect of the analytes was improved due to the introduction of nano-MgO. As a summary of the above results, the improved method displayed a better ability to solve the complexity of PRA matrices.

The performance of the established method for PRA sample analysis was compared with other previously reported methods (Table 2). The present study was rapid, simple, and convenient due to the improvement of the traditional QuEChERS method. The advantages of the present method include low sample and organic solvent consumption, cheap purification material, and no special instrument required during the operation [11, 13, 28, 29]. The established method can simultaneously determine 123 pesticides in five different PRA samples to achieve high-throughput analysis and wide applicability. In addition, this method has outstanding advantages in reducing the matrix effect.

## 4. Conclusions

A rapid and efficient multiresidue analytical method was developed to detect 123 pesticides residues in various PRA samples with nano-MgO as dispersive solid-phase adsorbent. The cleanup adsorbent combination of nano-MgO, C18, and PSA was proved to be excellent in reducing the matrix effect, providing accurate quantitative results of the analytes. A systematic method validation for five different PRA samples was performed to evaluate linearity, LODs, matrix effect, recoveries, and RSDs, demonstrating the satisfactory sensitivity, precision, accuracy, and universality of the method. Furthermore, the developed method was effective, and with a high throughput when compared with other reported methods. Nano-MgO was used as a cleanup material for the first time to analyze pesticides residues in PRA samples, demonstrating an efficient purification performance and good application prospects in a complex matrix. The established method could fully meet the multiple pesticide residue analysis requirements for PRA samples.

## Figures and Tables

**Table 1:** Recoveries of the spiked pesticides from the various PRA samples at pesticide concentrations of 20, 50, and 200 *μ*g/kg and the number of pesticides not detected (ND) at each concentration.

Recoveries ± RSD (%) for pesticides and the number of nondetects at 20, 50, and 200 *μ*g/kg in each PRA							No. of pesticides with 70−120% recovery at 20, 50, and 200 *μ*g/kg in each PRA		
Samples	20 *μ*g/kg	ND^a^	50 *μ*g/kg	ND	200 *μ*g/kg	ND	20 *μ*g/kg	50 *μ*g/kg	200 *μ*g/kg
PRA-ZJ	103 ± 5.70	6	106 ± 4.06	0	101 ± 2.72	0	110	110	122
PRA-BZ	103 ± 5.04	8	111 ± 3.84	4	99.5 ± 2.73	0	108	107	121
PRA-SD	100 ± 4.87	13	107 ± 3.33	5	100 ± 2.93	0	109	104	120
PRA-HN	104 ± 4.15	6	108 ± 4.12	0	106 ± 3.09	0	106	107	121
PRA-HB	107 ± 4.43	17	98.0 ± 3.76	4	107 ± 2.88	0	97	117	116
Mean ± RSD	103 ± 4.83		106 ± 3.82		103 ± 2.87				
Minimum	100 ± 4.87		98.0 ± 3.76		99.5 ± 2.73				
Maximum	107 ± 4.43		111 ± 3.84		107 ± 2.88				

**Table 2:** Comparison of method performance with the reported method for analysis of *Paeoniae Radix Alba*.

Methods	Analytes	Amounts of samples (g)	Volume of organics (mL)	Purification materials	Mean recovery (%)	References
SPE-GC-MS/MS	99 pesticides	4	20	SPE column	66.7–128	[11]
dSPE-HPLC-MS	23 pesticides	5	20	PSA	70.0–120	[13]
SPE-UFLC	5 triazine herbicides	1	5	Molecularly imprinted polymers	92.4–107	[28]
SPE-GC-ECD	20 pesticides	5	40	Silica gel column	74.4–115	[29]
dSPE-LC-MS/MS	123 pesticides	2	8	Nano-MgO, PSA, C18	98.0–111	This work

## Data Availability

The data used to support the findings of this study are included within the supplementary information files owing to the equal contribution

## Abbreviations

C18: Octadecylsilane
dSPE: Dispersive solid-phase extraction
HAc: Acetic acid
HPLC: High-performance liquid chromatography
LC-MS/MS: Liquid chromatography-tandem mass spectrometry
MRM: Multiple reaction monitoring
MgSO_4_: Anhydrous magnesium sulfate
NaCl: Sodium chloride
PSA: Primary secondary amine
PRA: *Paeoniae Radix Alba*
RSD: Relative standard deviation.

## Supplementary Materials

Supplementary Materials The influence of the volume of the extraction solvent is shown in Figure S1. The gradient elution program is shown in Table S1. The optimal collision energy and MRM precursor ion, product ion, and declustering potential for all target analytes are shown in Table S2. The results of the matrix effect are shown in Tables S3–S7. The detailed results of the recovery rates and the relative standard deviation (RSD) for all pesticides in five different PRA samples at three concentration levels are shown in Tables S8–S12.
