# Supplementary material for: Removal of Matrix Interferences by Nano-MgO and Co-Adsorbents for Accurate Multi-Pesticide Residue Analysis in the Chinese Medicinal Herb, Paeoniae Radix Alba
Source: J Anal Methods Chem. 2021 Feb 4;2021:6626257. doi: 10.1155/2021/6626257 (PMC7880715; doi:10.1155/2021/6626257)
Supplement: Supplementary Materials — The influence of the volume of the extraction solvent is shown in Figure S1. The gradient elution program is shown in Table S1. The optimal collision energy and MRM precursor ion, product ion, and declustering potential for all target analytes are shown in Table S2. The results of the matrix effect are shown in Tables S3–S7. The detailed results of the recovery rates and the relative standard deviation (RSD) for all pesticides in five different PRA samples at three concentration levels are shown in Tables S8–S12. [file 6626257.f1.doc]

**Supplementary information**

**Removal of matrix interferences by nano MgO and co-adsorbents for accurate multi-pesticide residue analysis in the Chinese medicinal herb,** ***Paeoniae Radix Alba***

Chunyu Wang^a^, Xinquan Wang^b,c,d,1^, Jiao Wang^b,c,d^, Shanshan Di^b,c,d^, Zhiwei Wang^b,c,d^, Hao Xu^b,c,d^, Huiyu Zhao^b,c,d^, Changshan Zhao^a^,*, Peipei Qi ^b,c,d^*

^a^ College of Agriculture, Northeast Agricultural University, Harbin, 150030, P. R. China

^b^ State Key Laboratory for Managing Biotic and Chemical Threats to the Quality and Safety of Agro-products, Institute of Quality and Standard of Agro-products, Zhejiang Academy of Agricultural Sciences, Hangzhou 310021, P. R. China

^c^ Agricultural Ministry Key Laboratory for Pesticide Residue Detection, Hangzhou 310021, P. R. China

^d^ Key Laboratory of Detection for Pesticide Residue and Control of Zhejiang, Hangzhou 310021, P. R. China

***Corresponding author**

**Professor Changshan Zhao**

Postal address: No. 600 Changjiang Road, Harbin 150030, P. R. China

Tel: +86 451 55191775; Email address: csz-hlj@sohu.com

**Dr. Peipei Qi**

Postal address: No. 198 Shiqiao Road, Hangzhou 310021, P. R China

Tel: +86 571 86419051; E-mail address: qipeipei@zaas.ac.cn

^1^Xinquan Wang is the co-first author owing to the equal contribution.

**
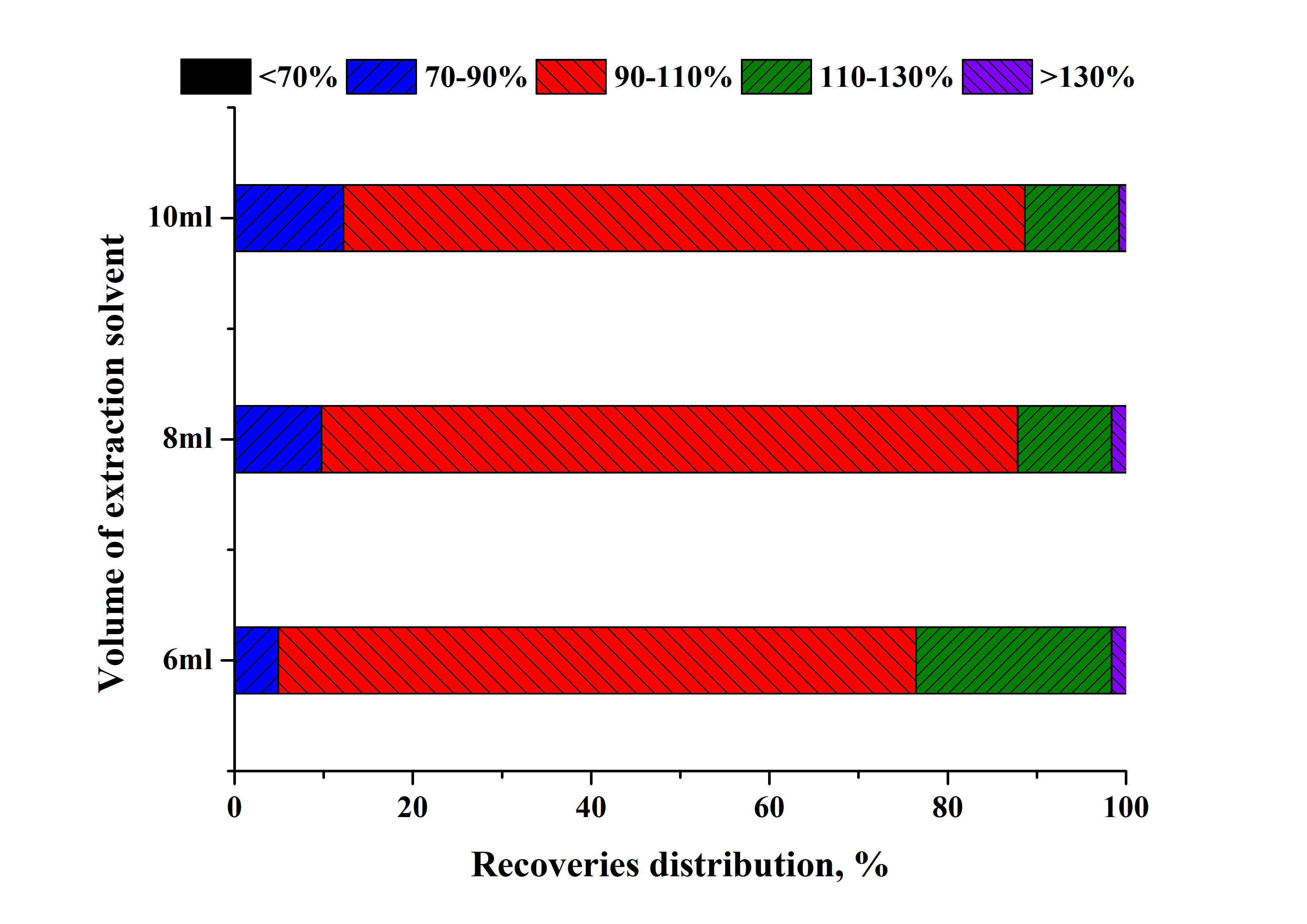
**

**Fig. S1** Influence of the extraction solvent volumes on the recovery distributions

Table S1 The gradient elution program of method, phase A: 5 mmol/L ammonium formate aqueous solution and phase B: methanol.

| time range (min) | the ratio of phase A (%) | the ratio of phase B (%) |
| --- | --- | --- |
| 0-1 | 95 | 5 |
| 1-3 | 60 | 40 |
| 3-5 | 20 | 80 |
| 5-8 | 5 | 95 |
| 8-10 | 95 | 5 |

Table S2 MS/MS parameters for the tested 123 pesticide.

| Pesticides | Parent ion (m/z) | Product ion | Q1 prerod | Collision energy (eV) | Q3 prerod |
| --- | --- | --- | --- | --- | --- |
|  |  | (m/z) | (V) |  | (V) |
| 3-Hydroxy-carbofuran | 238.1 | 163.1；181.2 | -27；-27 | -14；-10 | -17；-19 |
| Abamectin | 895.5 | 449.3；751.5 | -32；-32 | -46；-46 | -32；-22 |
| Acephate | 184.2 | 143；95 | -20；-20 | -8；-23 | -15；-16 |
| Acetamiprid | 223.1 | 126.05；56.1 | -30；-30 | -22；-15 | -30；-23 |
| Alachlor | 270.1 | 238.05；162.15 | -30；-30 | -10；-19 | -26；-30 |
| Atrazine | 216.1 | 174.05；96.05 | -30；-30 | -17；-25 | -18；-17 |
| Azaconazole | 300 | 159；231 | -15；-15 | -27；-17 | -29；-23 |
| Azamethiphos | 325 | 183.1；112.1 | -16；-16 | -16；-38 | -19；-20 |
| Azoxystrobin | 404.1 | 372.05；329 | -30；-30 | -14；-31 | -26；-23 |
| Benalaxyl | 326.2 | 148.2；294.1 | -16；-16 | -21；-11 | -15；-20 |
| Bendiocarb | 224.1 | 167.05；109.05 | -25；-25 | -9；-19 | -18；-20 |
| Bitertanol | 338.2 | 269.15；99.1 | -17；-17 | -9；-15 | -29；-18 |
| Boscalid | 343 | 307.1；271.1 | -12；-12 | -18；-30 | -30；-26 |
| Bromuconazole | 377.9 | 158.9；70 | -19；-19 | -28；-23 | -30；-30 |
| Buprofezin | 306.1 | 201.1；116.1 | -30；-30 | -11；-16 | -22；-12 |
| Butachlor | 312.2 | 238.1；147.2 | -15；-15 | -12；-36 | -26；-28 |
| Cadusafos | 271.1 | 159；97 | -30；-30 | -14；-37 | -29；-18 |
| Carbendazim | 192.05 | 160.05；132.05 | -30；-30 | -17；-30 | -30；-24 |
| Carbofuran | 222.1 | 165.1；123.1 | -25；-25 | -11；-21 | -17；-22 |
| Chlorantraniliprole | 484 | 452.9；285.85 | -24；-24 | -19；-16 | -30；-30 |
| Chlorpyrifos | 351.9 | 199.9；96.95 | -27；-27 | -18；-33 | -21；-18 |
| Chlortoluron | 213.1 | 72；140.1 | -23；-24 | -21；-23 | -28；-24 |
| Clothianidin | 250 | 169.1；132 | -29；-29 | -12；-14 | -17；-24 |
| Coumaphos | 363 | 227；307.1 | -18；-18 | -26；-18 | -23；-21 |
| Cyanophenphos | 304.1 | 157.25；77.25 | -15；-20 | -23；-60 | -12；-16 |
| Cyazofamid` | 325 | 108.05；261.1 | -23；-23 | -12；-10 | -21；-28 |
| Cyproconazole | 292.1 | 70.05；125.05 | -30；-30 | -20；-30 | -27；-22 |
| Cyprodinil | 226.1 | 93.1；108.1 | -30；-30 | -34；-27 | -16；-19 |
| Dicofol | 391.1 | 164.3；163.3 | -26；-19 | -22；-23 | -12；-18 |
| Dicrotophos | 238 | 112.1；193 | -12；-12 | -12；-9 | -11；-20 |
| Difenoconazole | 406.1 | 251；337.05 | -30；-30 | -25；-17 | -27；-24 |
| Dimethoate | 230 | 198.95；125 | -26；-26 | -9；-22 | -21；-22 |
| Dimethomorph | 388.1 | 301；165.05 | -19；-19 | -20；-34 | -21；-30 |
| dimethyl phthalate | 195.1 | 163.3；77.25 | -13；-13 | -10；-31 | -19；-15 |
| Diniconazole | 326.1 | 70；159 | -16；-16 | -25；-30 | -28；-30 |
| Dinotefuran | 203.1 | 129.1；113.1 | -22；-22 | -12；-10 | -22；-12 |
| Emamectin B1 Benzoate | 886.4 | 158.3；82.25 | -26；-26 | -40；-70 | -12；-17 |
| Epoxiconazole | 330.1 | 121.2；141.1 | -17；-17 | -21；-18 | -22；-25 |
| Ethiofencarb | 226.1 | 107.05；164.05 | -26；-26 | -15；-8 | -19；-30 |
| Etofenprox | 394 | 177.1；106.95 | -19；-19 | -17；-40 | -20；-19 |
| Etoxazole | 360 | 141；113 | -15；-15 | -30；-35 | -15；-15 |
| Fenamiphos | 304.1 | 217.1；202 | -15；-15 | -22；-36 | -23；-21 |
| Fenamiphos sulfoxide | 319.8 | 233；292.05 | -30；-30 | -23；-16 | -26；-21 |
| Fenbuconazole | 336.9 | 125.05；70 | -26；-26 | -27；-20 | -25；-28 |
| Fenpropathrin | 350.3 | 125.2；97.2 | -24；-25 | -18；-31 | -12；-17 |
| Fipronil | 435 | 330；250 | 10；10 | 16；28 | 21；24 |
| Fipronil-sulfone | 450.9 | 415；282.25 | 22；22 | 16；27 | 10；12 |
| Fludioxonil | 247.1 | 180.2；126.15 | 17；17 | 29；32 | 30；22 |
| Fluopyram | 397.1 | 173.1；208.1 | -28；-15 | -28；-22 | -24；-21 |
| Fluoxastrobin | 458.9 | 427.2；188.3 | -17；-22 | -20；-36 | -17；-14 |
| Flusilazole | 316.1 | 247.1；165.1 | -30；-30 | -18；-29 | -27；-30 |
| Flutolanil | 324.1 | 262.1；242 | -16；-16 | -18；-26 | -27；-25 |
| Flutriafol | 302.1 | 123；109 | -15；-15 | -28；-31 | -22；-19 |
| Forchlorfenuron | 248.1 | 129.1；93.1 | -30；-30 | -17；-34 | -23；-17 |
| Hexaconazole | 314.1 | 70.2；159.15 | -15；-15 | -21；-29 | -28；-30 |
| Hexaflumuron | 458.9 | 427.1；188.2 | -13；-22 | -18；-35 | -17；-14 |
| Hexazinone | 253.15 | 171.1；85.1 | -30；-30 | -15；-31 | -18；-15 |
| Imazalil | 297 | 159；200 | -15；-15 | -23；-19 | -15；-15 |
| Indoxacarb | 528.1 | 249.1；292.95 | -26；-26 | -17；-15 | -27；-21 |
| Ipconazole | 334 | 70.3；125.2 | -23；-23 | -23；-47 | -14；-14 |
| Isoprocarb | 194.1 | 95；137.1 | -21；-22 | -14；-10 | -17；-14 |
| Kresoxim-methyl | 314 | 222.3；267.3 | -15；-21 | -17；-8 | -12；-14 |
| Lufenuron | 509 | 326；339 | 24；24 | 19；12 | 15；22 |
| Malaoxon | 314.9 | 98.95；127 | -15；-15 | -24；-13 | -19；-23 |
| Malathion | 331 | 127.05；99 | -17；-17 | -12；-23 | -13；-18 |
| Mefenoxam | 280.1 | 220.15；248.1 | -30；-30 | -13；-10 | -24；-27 |
| Mepronil | 270.15 | 119.05；228.05 | -30；-30 | -25；-18 | -30；-30 |
| Metalaxyl | 280.1 | 220.2；192.2 | -30；-30 | -13；-18 | -24；-20 |
| Methidathion | 303 | 145；85.1 | -21；-21 | -8；-22 | -15；-30 |
| Methomyl | 163.05 | 88；106.05 | -18；-18 | -8；-10 | -16；-19 |
| Methoprene | 311.1 | 117.3；119.15 | -22；-15 | -52；-31 | -14；-22 |
| Methoxyfenozide | 369.2 | 149.1；313.1 | -18；-18 | -16；-8 | -16；-22 |
| Mevinphos | 225 | 127.05；193.05 | -25；-25 | -17；-8 | -23；-20 |
| Monocrotophos | 224.1 | 127.05；193 | -25；-25 | -15；-8 | -13；-20 |
| Myclobutanil | 289.1 | 70.05；125.05 | -30；-30 | -21；-30 | -28；-22 |
| Omethoate | 214.1 | 183；155 | -23；-23 | -10；-14 | -19；-28 |
| Paclobutrazol | 294.1 | 70.05；125.05 | -15；-15 | -21；-40 | -28；-22 |
| Penconazole | 284.1 | 159；70 | -14；-14 | -27；-17 | -30；-27 |
| Phenthoate | 321 | 79.1；246.95 | -23；-23 | -41；-11 | -30；-17 |
| Phorate | 261 | 75；143 | -29；-29 | -10；-18 | -30；-15 |
| Phorate sulfone | 293 | 171.05；115 | -22；-22 | -9；-24 | -19；-20 |
| Phosalone | 368 | 182.05；111 | -30；-30 | -14；-39 | -19；-20 |
| Phosfolan | 256 | 140；228 | -13；-13 | -24；-12 | -26；-25 |
| Phosmet | 318 | 160；77.1 | -16；-16 | -13；-54 | -17；-30 |
| Phosphamidon | 300 | 174.05；127 | -15；-15 | -12；-29 | -17；-22 |
| Piperonyl butoxide | 356.3 | 177.1；119 | -24；-24 | -13；-37 | -19；-22 |
| Pirimicarb | 239.15 | 72.05；182.15 | -30；-30 | -25；-19 | -30；-30 |
| Pirimiphos-methyl | 306.05 | 108.1；95 | -30；-30 | -31；-29 | -19；-17 |
| Prochloraz | 376 | 308；266 | -19；-19 | -11；-17 | -21；-29 |
| Profenofos | 372.9 | 302.8；345 | -18；-18 | -19；-12 | -30；-24 |
| Propamocarb | 189.2 | 102.05；144.05 | -30；-30 | -20；-12 | -23；-15 |
| Propiconazole | 342.05 | 159.1；205.1 | -17；-17 | -30；-18 | -29；-21 |
| Prothiophos | 344.8 | 240.9；275 | -17；-17 | -18；-13 | -26；-30 |
| Pyraclostrobin | 388 | 163.3；164.35 | -11；-19 | -24；-18 | -18；-12 |
| Pyridaben | 365.1 | 309.05；147.1 | -18；-18 | -12；-25 | -22；-27 |
| Pyridafenthion | 341.1 | 189.1；205.1 | -17；-23 | -22；-22 | -20；-22 |
| Pyrimethanil | 200.1 | 107；168.1 | -30；-30 | -25；-29 | -19；-30 |
| Pyrimitate | 334.1 | 198.05；182.1 | -25；-25 | -22；-22 | -21；-19 |
| Pyriproxyfen | 322 | 96；184 | -15；-15 | -16；-24 | -15；-15 |
| Pyrisoxazole | 289 | 151；120 | -15；-15 | -20；-28 | -15；-15 |
| Quizalofop-p-ethyl | 373.1 | 299.05；91.05 | -19；-19 | -19；-32 | -21；-16 |
| Simeconazole | 294.1 | 70.05；135.05 | -15；-15 | -21；-21 | -28；-24 |
| Spirodiclofen | 411.1 | 71.2；313.05 | -21；-21 | -16；-11 | -28；-22 |
| Sulfotep | 323 | 115；171.1 | -16；-16 | -31；-15 | -20；-18 |
| Sulprofos | 322.9 | 219.05；139.25 | -16；-16 | -16；-30 | -16；-15 |
| Tebuconazole | 308.1 | 70.1；125 | -22；-22 | -22；-38 | -27；-23 |
| Tebufenozide | 353.2 | 133.1；297.1 | -18；-18 | -20；-8 | -24；-15 |
| Tebufenpyrad | 334.2 | 117.05；145 | -16；-16 | -38；-27 | -21；-26 |
| Temephos | 467 | 419；341 | -23；-23 | -19；-32 | -30；-24 |
| Terbufos | 289 | 103.25；57.2 | -14；-20 | -10；-20 | -12；-30 |
| Tetrachlorvinphos | 364.9 | 127.05；203.9 | -27；-27 | -14；-38 | -13；-21 |
| Tetraconazole | 372 | 159.2；70.2 | -25；-25 | -27；-23 | -18；-14 |
| Thiacloprid | 253 | 126.05；99 | -28；-28 | -20；-43 | -22；-17 |
| Thiamethoxam | 292 | 211.1；181.1 | -30；-30 | -11；-23 | -22；-19 |
| Thiophanate-methyl | 343.05 | 151.15；311.05 | -17；-17 | -20；-10 | -16；-22 |
| Tolfenpyrad | 384.1 | 197.1；154.05 | -10；-10 | -25；-41 | -12；-29 |
| Triadimefon | 294.1 | 69.15；197.05 | -21；-21 | -22；-15 | -26；-21 |
| Triadimenol | 296.1 | 70.05；99.15 | -15；-15 | -11；-15 | -29；-17 |
| Tricyclazole | 190 | 163；136 | -21；-21 | -21；-26 | -30；-24 |
| Trifloxystrobin | 409.1 | 186.05；145 | -20；-20 | -18；-44 | -20；-26 |
| Triticonazole | 318.1 | 70.05；125.05 | -16；-16 | -21；-39 | -28；-23 |
| Uniconazole | 292.1 | 70.1；125 | -21；-21 | -24；-28 | -27；-23 |
| Vitavax | 236.05 | 143；124 | -27；-27 | -14；-20 | -15；-22 |

Table S3 The method validation of the linearity, matrix effect and LODs of each pesticide in PRA-ZJ.

| Pesticides | ^1^y=a x+b | R | ^2^Slope ratio | Linear range, | ^3^LOD, |
| --- | --- | --- | --- | --- | --- |
|  |  |  |  | μg/L | μg/kg |
| 3-Hydroxy-carbofuran | ^4^y = 3302.52x - 3082.26 | 0.9986 | 0.15 | 0.5-250 | 4.68 |
|  | ^5^y = 22478.1x - 8901.30 | 0.9997 |  | 0.5-250 | 0.28 |
| Abamectin | y = 6404.04x - 14175.5 | 0.9952 | 1.83 | 2-250 | 2.74 |
|  | y = 3495.24x + 1957.13 | 0.9999 |  | 1-250 | 0.25 |
| Acephate | y = 84426.1x + 28437.7 | 0.9999 | 0.87 | 0.5-250 | 4.27 |
|  | y = 96800.2x - 3819.54 | 0.9996 |  | 0.5-250 | 1.59 |
| Acetamiprid | y = 30012.2x + 51588.4 | 0.9994 | 0.13 | 0.5-250 | 3.16 |
|  | y = 223248x + 47946 | 0.9996 |  | 0.5-250 | 1.81 |
| Alachlor | y = 723805x + 4426990 | 0.9897 | 0.91 | 0.5-250 | 4.23 |
|  | y = 792318x + 4126980 | 0.9984 |  | 0.5-250 | 1.76 |
| Atrazine | y = 162386x - 78599.7 | 0.9989 | 0.80 | 0.5-250 | 2.48 |
|  | y = 202545x + 326109 | 0.9992 |  | 0.5-250 | 0.21 |
| Azaconazole | y = 205922x - 115757 | 0.9977 | 0.77 | 0.5-250 | 1.22 |
|  | y = 268372x + 133688 | 0.9995 |  | 0.5-250 | 1.94 |
| Azamethiphos | y = 44904.4x - 46035.7 | 0.9968 | 0.46 | 0.5-250 | 0.16 |
|  | y = 98024.9x - 20835.8 | 0.9999 |  | 0.5-250 | 1.76 |
| Azoxystrobin | y = 557610x + 7497.24 | 0.9999 | 0.75 | 0.5-100 | 4.45 |
|  | y = 743784x + 71347.5 | 0.9997 |  | 0.5-100 | 0.51 |
| Benalaxyl | y = 169506x + 39831.1 | 0.9959 | 1.01 | 0.5-250 | 0.30 |
|  | y = 167645x - 185188 | 0.9997 |  | 0.5-250 | 1.46 |
| Bendiocarb | y = 9705.74x + 1977.36 | 0.9995 | 0.10 | 0.5-250 | 1.35 |
|  | y = 96648.3x + 211203 | 0.9991 |  | 0.5-250 | 0.84 |
| Bitertanol | y = 6046.23x + 14038.4 | 0.9977 | 1.16 | 1-250 | 2.64 |
|  | y = 5224.81x + 10194.4 | 0.9993 |  | 0.5-250 | 1.59 |
| Boscalid | y = 101574x - 5702.06 | 0.9970 | 1.07 | 0.5-200 | 4.67 |
|  | y = 95363.2x + 84231.6 | 0.9994 |  | 0.5-250 | 0.91 |
| Bromuconazole | y = 31400.1x + 21463.7 | 0.9979 | 0.93 | 0.5-250 | 2.00 |
|  | y = 33592.1x + 11743.8 | 0.9997 |  | 0.5-250 | 0.74 |
| Buprofezin | y = 290283x + 315494 | 0.9982 | 0.96 | 0.5-250 | 0.44 |
|  | y = 301316x + 498285 | 0.9993 |  | 0.5-250 | 0.85 |
| Butachlor | y = 164648x + 831062 | 0.9956 | 1.03 | 0.5-250 | 3.11 |
|  | y = 160435x + 619420 | 0.9981 |  | 0.5-250 | 0.79 |
| Cadusafos | y = 319422x + 380141 | 0.9986 | 1.01 | 0.5-250 | 4.59 |
|  | y = 315842x + 527213 | 0.9994 |  | 0.5-250 | 0.62 |
| Carbendazim | y = 103378x + 69803.7 | 0.9980 | 0.31 | 0.5-250 | 4.60 |
|  | y = 334448x + 280153 | 0.9993 |  | 0.5-250 | 1.21 |
| Carbofuran | y = 35014.5x - 18424 | 0.9995 | 0.12 | 0.5-250 | 2.11 |
|  | y = 288008x + 235300 | 0.9992 |  | 0.5-250 | 0.64 |
| Chlorantraniliprole | y = 50253.7x + 163945 | 0.9996 | 0.86 | 0.5-250 | 0.40 |
|  | y = 58737.3x + 167462 | 0.9993 |  | 0.5-250 | 0.57 |
| Chlorpyrifos | y = 732469x + 1162650 | 0.9977 | 0.95 | 0.5-100 | 1.15 |
|  | y = 771286x + 1044540 | 0.9988 |  | 0.5-100 | 1.25 |
| Chlortoluron | y = 120215x + 91037.8 | 0.9974 | 0.45 | 0.5-250 | 3.09 |
|  | y = 265439x + 701160 | 0.9989 |  | 0.5-250 | 1.68 |
| Clothianidin | y = 3799x + 5644.90 | 0.9985 | 0.06 | 2-250 | 2.96 |
|  | y = 63358x + 45132.3 | 0.9998 |  | 0.5-250 | 1.08 |
| Coumaphos | y = 205071x + 383167 | 0.9970 | 0.98 | 0.5-250 | 4.50 |
|  | y = 209361x + 639593 | 0.9992 |  | 0.5-250 | 0.86 |
| Cyanophenphos | y = 36183.6x + 119645 | 0.9995 | 0.98 | 0.5-250 | 4.86 |
|  | y = 36866.1x + 100916 | 0.9992 |  | 0.5-250 | 0.84 |
| Cyazofamid` | y = 57852.5x - 22089.1 | 0.9992 | 0.73 | 0.5-200 | 3.05 |
|  | y = 79298.7x + 188035 | 0.9991 |  | 0.5-250 | 0.52 |
| Cyproconazole | y = 84764.2x + 223322 | 0.9995 | 0.86 | 0.5-250 | 0.83 |
|  | y = 98745.4x + 278702 | 0.9993 |  | 0.5-250 | 1.23 |
| Cyprodinil | y = 128117x - 226037 | 0.9966 | 0.94 | 0.5-250 | 3.74 |
|  | y = 135850x + 86987.3 | 0.9995 |  | 0.5-250 | 1.37 |
| Dicofol | y = 8393.97x + 11174 | 0.9989 | 0.99 | 0.5-100 | 0.63 |
|  | y = 8500.96x - 2216.31 | 0.9980 |  | 0.5-100 | 1.61 |
| Dicrotophos | y = 4914.45x - 3773.08 | 0.9992 | 0.09 | 0.5-250 | 1.10 |
|  | y = 53568.7x - 4127.35 | 0.9998 |  | 0.5-250 | 0.43 |
| Difenoconazole | y = 156726x - 61458.9 | 0.9983 | 1.14 | 0.5-250 | 3.59 |
|  | y = 137817x - 48437.3 | 0.9998 |  | 0.5-250 | 2.00 |
| Dimethoate | y = 50854.5x - 21182.9 | 0.9989 | 0.24 | 0.5-250 | 0.88 |
|  | y = 213398x - 125164 | 0.9999 |  | 0.5-250 | 1.49 |
| Dimethomorph | y = 197962x + 276768 | 0.9990 | 1.69 | 0.5-250 | 0.25 |
|  | y = 116953x + 178724 | 0.9993 |  | 0.5-250 | 1.05 |
| dimethyl phthalate | y = 81744.7x + 1390850 | 0.9970 | 0.70 | 0.5-250 | 4.46 |
|  | y = 117004x + 1609990 | 0.9995 |  | 0.5-250 | 0.42 |
| Diniconazole | y = 46363.2x + 1368.96 | 0.9979 | 1.02 | 0.5-250 | 4.89 |
|  | y = 45491.1x + 94764.9 | 0.9990 |  | 0.5-250 | 0.89 |
| Dinotefuran | y = 40020.9x + 24098.5 | 0.9998 | 0.67 | 0.5-250 | 1.51 |
|  | y = 59625.9x + 44005.2 | 0.9997 |  | 0.5-250 | 1.02 |
| Emamectin B1 Benzoate | y = 343901x - 263881 | 0.9991 | 1.04 | 0.5-250 | 3.89 |
|  | y = 331424x - 70644.4 | 0.9992 |  | 0.5-250 | 0.38 |
| Epoxiconazole | y = 83608.8x + 13089 | 0.9985 | 0.67 | 0.5-250 | 0.82 |
|  | y = 124881x - 43441 | 0.9992 |  | 0.5-250 | 0.24 |
| Ethiofencarb | y = 370730x + 16550.4 | 0.9995 | 0.26 | 0.5-50 | 1.02 |
|  | y = 1422230x + 597334 | 0.9961 |  | 0.5-50 | 1.36 |
| Etofenprox | y = 266935x - 268970 | 0.9987 | 1.16 | 0.5-250 | 1.90 |
|  | y = 229507x + 101162 | 0.9995 |  | 0.5-250 | 0.31 |
| Etoxazole | y = 742602x + 553548 | 0.9997 | 1.15 | 0.5-100 | 0.29 |
|  | y = 643096x + 534898 | 0.9997 |  | 0.5-100 | 0.58 |
| Fenamiphos | y = 360443x + 1812850 | 0.9927 | 0.94 | 0.5-250 | 2.52 |
|  | y = 381984x + 1564290 | 0.9990 |  | 0.5-250 | 1.51 |
| Fenamiphos sulfoxide | y = 42343.2x - 35453.4 | 0.9981 | 0.30 | 0.5-250 | 2.10 |
|  | y = 142107x + 36275.4 | 0.9999 |  | 0.5-250 | 1.56 |
| Fenbuconazole | y = 60642.2x + 74777.1 | 0.9989 | 0.78 | 0.5-250 | 1.94 |
|  | y = 77275.9x - 195151 | 0.9990 |  | 0.5-250 | 1.25 |
| Fenpropathrin | y = 11077.2x + 9074.67 | 0.9983 | 1.13 | 0.5-100 | 1.33 |
|  | y = 9836.11x + 22907.2 | 0.9966 |  | 0.5-100 | 1.24 |
| Fipronil | y = 8066.59x + 8889.87 | 0.9987 | 0.75 | 0.5-250 | 3.48 |
|  | y = 10769.2x - 17592.3 | 0.9991 |  | 0.5-250 | 0.62 |
| Fipronil-sulfone | y = 138882x - 56464.3 | 0.9988 | 0.75 | 0.5-250 | 4.22 |
|  | y = 183973x - 50212.4 | 0.9994 |  | 0.5-250 | 1.64 |
| Fludioxonil | y = 27074.1x + 158488 | 0.9991 | 0.88 | 0.5-250 | 1.07 |
|  | y = 30914.8x + 86835 | 0.9992 |  | 0.5-250 | 0.19 |
| Fluopyram | y = 196141x - 49557.1 | 0.9985 | 0.91 | 0.5-250 | 3.27 |
|  | y = 216694x - 397443 | 0.9993 |  | 0.5-250 | 0.44 |
| Fluoxastrobin | y = 326150x + 949835 | 0.9975 | 1.00 | 0.5-250 | 4.36 |
|  | y = 326343x + 835206 | 0.9994 |  | 0.5-250 | 2.00 |
| Flusilazole | y = 141573x + 84847.5 | 0.9932 | 0.90 | 0.5-250 | 2.32 |
|  | y = 157601x - 219056 | 0.9993 |  | 0.5-250 | 1.09 |
| Flutolanil | y = 403616x + 454141 | 0.9993 | 0.67 | 0.5-100 | 3.90 |
|  | y = 604339x - 162788 | 0.9997 |  | 0.5-100 | 1.70 |
| Flutriafol | y = 23428.9x + 9128.77 | 0.9976 | 0.51 | 1-250 | 4.22 |
|  | y = 45577x - 12136.6 | 0.9995 |  | 0.5-250 | 1.93 |
| Forchlorfenuron | y = 78054.1x + 18396.6 | 0.9993 | 0.89 | 0.5-250 | 3.11 |
|  | y = 87807.5x + 280399 | 0.9992 |  | 0.5-250 | 1.86 |
| Hexaconazole | y = 70878.2x - 35592.2 | 0.9982 | 1.06 | 0.5-250 | 3.30 |
|  | y = 66860.6x + 53485.5 | 0.9995 |  | 0.5-250 | 1.26 |
| Hexaflumuron | y = 362268x + 2102490 | 0.9984 | 1.04 | 0.5-250 | 3.39 |
|  | y = 349339x + 1178920 | 0.9995 |  | 0.5-250 | 1.33 |
| Hexazinone | y = 271536x - 79220.1 | 0.9999 | 0.38 | 0.5-100 | 3.08 |
|  | y = 717261x + 309539 | 0.9998 |  | 0.5-100 | 0.97 |
| Imazalil | y = 92646.8x + 24754.3 | 0.9991 | 0.91 | 0.5-250 | 4.85 |
|  | y = 101466x + 89024.5 | 0.9996 |  | 0.5-250 | 1.26 |
| Indoxacarb | y = 22053.5x + 9673.58 | 0.9996 | 1.14 | 0.5-200 | 3.83 |
|  | y = 19396x + 47574.7 | 0.9991 |  | 0.5-250 | 1.90 |
| Ipconazole | y = 130829x + 106976 | 0.9995 | 0.76 | 0.5-250 | 1.91 |
|  | y = 173211x + 311393 | 0.9992 |  | 0.5-250 | 0.90 |
| Isoprocarb | y = 112974x + 81884.8 | 0.9997 | 0.53 | 0.5-250 | 4.86 |
|  | y = 213432x - 324303 | 0.9991 |  | 0.5-250 | 0.62 |
| Kresoxim-methyl | y = 48766x + 3777.24 | 0.9988 | 0.80 | 0.5-100 | 3.82 |
|  | y = 60608.7x - 32216 | 0.9997 |  | 0.5-100 | 0.90 |
| Lufenuron | y = 13450.3x + 79497.3 | 0.9902 | 1.02 | 0.5-200 | 4.55 |
|  | y = 13251.2x + 78340.9 | 0.9980 |  | 0.5-250 | 0.76 |
| Malaoxon | y = 43209.6x - 10351.7 | 0.9996 | 0.05 | 0.5-100 | 3.00 |
|  | y = 839041x + 501694 | 0.9998 |  | 0.5-100 | 0.79 |
| Malathion | y = 115977x + 130014 | 0.9987 | 0.88 | 0.5-250 | 0.81 |
|  | y = 132104x + 396108 | 0.9994 |  | 0.5-250 | 1.36 |
| Mefenoxam | y = 535008x - 33425.7 | 0.9989 | 0.83 | 0.5-100 | 1.49 |
|  | y = 646361x + 696020 | 0.9995 |  | 0.5-100 | 1.24 |
| Mepronil | y = 1829080x + 4063210 | 0.9988 | 0.58 | 0.5-50 | 2.97 |
|  | y = 3175610x + 459022 | 0.9997 |  | 0.5-50 | 0.67 |
| Metalaxyl | y = 520642x - 43462.9 | 0.9999 | 0.73 | 0.5-100 | 1.36 |
|  | y = 718117x + 142354 | 0.9999 |  | 0.5-100 | 1.77 |
| Methidathion | y = 30902.3x + 9751.63 | 0.9996 | 0.80 | 0.5-250 | 3.53 |
|  | y = 38779.7x + 8462.38 | 0.9997 |  | 0.5-250 | 0.61 |
| Methomyl | y = 37618.9x - 18907.3 | 0.9998 | 0.53 | 0.5-250 | 3.81 |
|  | y = 70897.1x - 100837 | 0.9996 |  | 0.5-250 | 0.83 |
| Methoprene | y = 24285x + 13726.3 | 0.9994 | 1.02 | 0.5-250 | 0.23 |
|  | y = 23855.3x + 1560.05 | 0.9994 |  | 0.5-250 | 1.17 |
| Methoxyfenozide | y = 70220.1x + 268363 | 0.9991 | 0.95 | 0.5-250 | 3.78 |
|  | y = 74074.9x + 70085.8 | 0.9992 |  | 0.5-250 | 1.52 |
| Mevinphos | y = 19920.4x - 16010.4 | 0.9996 | 0.30 | 0.5-250 | 0.31 |
|  | y = 66496x - 64412.2 | 0.9995 |  | 0.5-250 | 1.88 |
| Monocrotophos | y = 4367.03x - 5767.3 | 0.9995 | 0.13 | 0.5-250 | 0.73 |
|  | y = 34513.8x + 11875.1 | 0.9997 |  | 0.5-250 | 0.19 |
| Myclobutanil | y = 69781.4x + 118497 | 0.9950 | 1.04 | 0.5-200 | 0.41 |
|  | y = 67118.4x + 142114 | 0.9997 |  | 0.5-250 | 1.92 |
| Omethoate | y = 69357.9x - 30617.4 | 0.9993 | 0.77 | 0.5-250 | 4.91 |
|  | y = 89900.3x - 116146 | 0.9998 |  | 0.5-250 | 1.37 |
| Paclobutrazol | y = 141377x + 431865 | 0.9986 | 0.81 | 0.5-250 | 1.68 |
|  | y = 174702x + 90474.6 | 0.9990 |  | 0.5-250 | 0.68 |
| Penconazole | y = 103751x + 96129.6 | 0.9979 | 1.13 | 0.5-200 | 2.90 |
|  | y = 91994.6x + 140739 | 0.9991 |  | 0.5-250 | 0.51 |
| Phenthoate | y = 150519x + 176095 | 0.9969 | 1.11 | 0.5-200 | 2.71 |
|  | y = 135636x + 493223 | 0.9994 |  | 0.5-250 | 0.52 |
| Phorate | y = 34283.8x - 13671.3 | 0.9944 | 0.94 | 0.5-250 | 3.19 |
|  | y = 36585.5x + 41822.3 | 0.9990 |  | 0.5-250 | 0.78 |
| Phorate sulfone | y = 24515.4x + 22000.3 | 0.9987 | 0.44 | 0.5-100 | 2.12 |
|  | y = 55229x + 44723.2 | 0.9995 |  | 0.5-100 | 1.97 |
| Phosalone | y = 1344280x + 499794 | 0.9981 | 1.05 | 0.5-50 | 4.58 |
|  | y = 1281220x + 826217 | 0.9988 |  | 0.5-50 | 0.16 |
| Phosfolan | y = 161270x - 15228.9 | 0.9975 | 0.46 | 0.5-250 | 2.83 |
|  | y = 351640x + 793462 | 0.9993 |  | 0.5-250 | 1.99 |
| Phosmet | y = 146241x - 77796.9 | 0.9923 | 0.78 | 0.5-250 | 3.79 |
|  | y = 187056x + 250378 | 0.9999 |  | 0.5-250 | 1.43 |
| Phosphamidon | y = 90604.5x - 17640 | 0.9982 | 0.70 | 0.5-250 | 1.49 |
|  | y = 128857x - 145106 | 0.9998 |  | 0.5-250 | 1.88 |
| Piperonyl butoxide | y = 339688x + 786950 | 0.9974 | 1.03 | 0.5-250 | 3.79 |
|  | y = 328481x + 511020 | 0.9992 |  | 0.5-250 | 1.87 |
| Pirimicarb | y = 221551x - 7734.98 | 0.9992 | 0.66 | 0.5-250 | 3.88 |
|  | y = 335094x + 1196200 | 0.9990 |  | 0.5-250 | 0.18 |
| Pirimiphos-methyl | y = 320053x + 434766 | 0.9986 | 0.99 | 0.5-250 | 1.01 |
|  | y = 322697x + 282327 | 0.9994 |  | 0.5-250 | 0.22 |
| Prochloraz | y = 171649x + 365749 | 0.9990 | 0.93 | 0.5-250 | 4.69 |
|  | y = 184363x - 38432 | 0.9993 |  | 0.5-250 | 0.81 |
| Profenofos | y = 89950.9x + 291703 | 0.9965 | 0.80 | 0.5-250 | 3.79 |
|  | y = 112112x + 241932 | 0.9991 |  | 0.5-250 | 1.33 |
| Propamocarb | y = 194272x - 75091.2 | 0.9992 | 0.74 | 0.5-250 | 4.75 |
|  | y = 264294x - 325221 | 0.9996 |  | 0.5-250 | 1.38 |
| Propiconazole | y = 95367.3x + 230703 | 0.9996 | 0.93 | 0.5-250 | 4.63 |
|  | y = 102586x + 216541 | 0.9994 |  | 0.5-250 | 0.66 |
| Prothiophos | y = 50573.8x - 157597 | 0.9969 | 1.04 | 1-250 | 2.16 |
|  | y = 48650.7x - 3180.76 | 0.9980 |  | 1-250 | 1.97 |
| Pyraclostrobine | y = 172818x + 400179 | 0.9996 | 0.93 | 0.5-250 | 3.54 |
|  | y = 186375x + 307622 | 0.9992 |  | 0.5-250 | 1.53 |
| Pyridaben | y = 266491x - 80041.8 | 0.9993 | 1.02 | 0.5-250 | 3.28 |
|  | y = 260273x + 50248.3 | 0.9998 |  | 0.5-250 | 0.53 |
| Pyridafenthion | y = 198197x + 1091860 | 0.9995 | 0.97 | 0.5-250 | 3.83 |
|  | y = 204024x + 191747 | 0.9994 |  | 0.5-250 | 0.63 |
| Pyrimethanil | y = 58301.8x + 85136.1 | 0.9969 | 0.89 | 0.5-250 | 2.27 |
|  | y = 65358.3x + 120705 | 0.9994 |  | 0.5-250 | 1.69 |
| Pyrimitate | y = 842196x + 1238750 | 0.9973 | 1.01 | 0.5-100 | 3.09 |
|  | y = 835352x + 732844 | 0.9995 |  | 0.5-100 | 1.12 |
| Pyriproxyfen | y = 363072x + 1202260 | 0.9971 | 1.07 | 0.5-250 | 1.96 |
|  | y = 339296x + 923521 | 0.9990 |  | 0.5-250 | 1.56 |
| Pyrisoxazole | y = 10939x + 54284.2 | 0.9966 | 1.05 | 1-250 | 3.31 |
|  | y = 10389.2x + 31131 | 0.9991 |  | 0.5-250 | 0.30 |
| Quizalofop-p-ethyl | y = 333350x - 257210 | 0.9982 | 1.00 | 0.5-250 | 0.16 |
|  | y = 331894x + 248432 | 0.9993 |  | 0.5-250 | 1.54 |
| Simeconazole | y = 140222x + 45221.7 | 0.9997 | 0.71 | 0.5-250 | 4.57 |
|  | y = 196491x - 29091.1 | 0.9995 |  | 0.5-250 | 1.96 |
| Spirodiclofen | y = 42572x + 23492.6 | 0.9992 | 0.92 | 0.5-250 | 3.67 |
|  | y = 46401.8x + 13914.9 | 0.9998 |  | 0.5-250 | 0.49 |
| Sulfotep | y = 199351x + 198833 | 0.9988 | 0.88 | 0.5-250 | 2.96 |
|  | y = 225425x + 566533 | 0.9994 |  | 0.5-250 | 1.27 |
| Sulprofos | y = 135890x - 264343 | 0.9962 | 0.98 | 0.5-250 | 2.29 |
|  | y = 138275x - 150856 | 0.9992 |  | 0.5-250 | 1.84 |
| Tebuconazole | y = 154232x + 127002 | 0.9989 | 0.96 | 0.5-250 | 4.32 |
|  | y = 160048x + 13926.9 | 0.9998 |  | 0.5-250 | 1.26 |
| Tebufenozide | y = 57827.9x + 75359.9 | 0.9975 | 0.98 | 0.5-250 | 3.89 |
|  | y = 59088.7x + 32806.8 | 0.9999 |  | 0.5-250 | 1.02 |
| Tebufenpyrad | y = 101553x + 16590.6 | 0.9987 | 0.96 | 0.5-250 | 2.12 |
|  | y = 106275x - 191860 | 0.9991 |  | 0.5-250 | 1.57 |
| Temephos | y = 345557x + 1110850 | 0.9956 | 0.99 | 0.5-250 | 1.98 |
|  | y = 347483x + 1162690 | 0.9994 |  | 0.5-250 | 0.71 |
| Terbufos | y = 8763.28x + 32155 | 0.9970 | 1.03 | 0.5-250 | 2.41 |
|  | y = 8518.68x + 33819.1 | 0.9992 |  | 0.5-250 | 1.92 |
| Tetrachlorvinphos | y = 34853.3x + 171712 | 0.9991 | 0.93 | 0.5-250 | 4.27 |
|  | y = 37520.1x + 71579.9 | 0.9998 |  | 0.5-250 | 0.68 |
| Tetraconazole | y = 155157x + 115193 | 0.9993 | 0.90 | 0.5-100 | 3.53 |
|  | y = 172206x + 18684.8 | 0.9996 |  | 0.5-100 | 1.01 |
| Thiacloprid | y = 223298x - 128730 | 0.9998 | 0.54 | 0.5-250 | 2.32 |
|  | y = 416872x + 1377520 | 0.9993 |  | 0.5-250 | 2.00 |
| Thiamethoxam | y = 30676.3x - 25019.3 | 0.9977 | 0.22 | 0.5-250 | 0.23 |
|  | y = 139911x + 4966.57 | 0.9999 |  | 0.5-250 | 1.81 |
| Thiophanate-methyl | y = 67353.8x - 285885 | 0.9885 | 0.20 | 0.5-250 | 4.21 |
|  | y = 338599x + 515079 | 0.9995 |  | 0.5-250 | 1.40 |
| Tolfenpyrad | y = 96813.9x + 259251 | 0.9982 | 1.00 | 0.5-250 | 4.12 |
|  | y = 96485.1x + 162015 | 0.9994 |  | 0.5-250 | 0.99 |
| Triadimefon | y = 45302x + 126042 | 0.9972 | 0.79 | 0.5-250 | 3.20 |
|  | y = 57301.5x + 90066.9 | 0.9994 |  | 0.5-250 | 1.33 |
| Triadimenol | y = 26107.4x + 5564.72 | 0.9993 | 0.94 | 0.5-250 | 3.76 |
|  | y = 27795.9x + 4696.08 | 0.9999 |  | 0.5-250 | 1.77 |
| Tricyclazole | y = 82844.8x - 40560.7 | 0.9986 | 0.35 | 0.5-250 | 3.81 |
|  | y = 234231x + 317911 | 0.9995 |  | 0.5-250 | 1.84 |
| Trifloxystrobin | y = 428439x + 2513340 | 0.9914 | 1.19 | 0.5-250 | 1.42 |
|  | y = 361172x + 1671560 | 0.9984 |  | 0.5-250 | 0.38 |
| Triticonazole | y = 66890.5x - 50047.2 | 0.9991 | 0.66 | 0.5-250 | 3.30 |
|  | y = 100738x - 76418.6 | 0.9994 |  | 0.5-250 | 1.78 |
| Uniconazole | y = 92772.4x + 257855 | 0.9995 | 0.88 | 0.5-250 | 2.80 |
|  | y = 106013x + 353989 | 0.9992 |  | 0.5-250 | 1.52 |
| Vitavax | y = 1205580x + 303798 | 0.9977 | 0.36 | 0.5-50 | 1.36 |
|  | y = 3312810x - 605463 | 0.9996 |  | 0.5-50 | 1.81 |

^1^ Calibration curves are expressed as regression lines (y = a x + b), where y is the peak area and x is the concentration (μg/L), a is the slope, b is the intercept and R is the correlation coefficient.

^2^ The value of slope ratio is calculated by the slope of the matrix-matched calibration curve to the slope of the standard calibration curve in solvent.

^3^ The LOD was the three times of the singal to noise ratio.

^4^ The first calibration curve for each pesticide represents the matrix-matched calibration curve.

^5^ The second calibration curve for each pesticide represents the standard calibration curve in solvent.

Table S4 The method validation of the linearity, matrix effect and LODs of each pesticide in PRA-AH.

| Pesticides | ^1^y=a x+b | R | ^2^Sloperatio | Linear range, | ^3^LOD, |
| --- | --- | --- | --- | --- | --- |
|  |  |  |  | μg/L | μg/kg |
| 3-Hydroxy-carbofuran | ^4^y = 3624.97x - 11753.5 | 0.9973 | 0.16 | 1-250 | 1.50 |
|  | ^5^y = 22478.1x - 8901.3 | 0.9997 |  | 0.5-250 | 0.28 |
| Abamectin | y = 3914.44x + 5931.39 | 0.9982 | 1.12 | 1-200 | 2.68 |
|  | y = 3495.24x + 1957.13 | 0.9999 |  | 1-250 | 0.25 |
| Acephate | y = 85922.3x - 92403.9 | 0.9997 | 0.89 | 0.5-250 | 2.92 |
|  | y = 96800.2x - 3819.54 | 0.9996 |  | 0.5-250 | 1.59 |
| Acetamiprid | y = 38250x - 59618.6 | 0.9990 | 0.17 | 0.5-250 | 0.51 |
|  | y = 223248x + 47946 | 0.9996 |  | 0.5-250 | 1.81 |
| Alachlor | y = 769495x + 4679370 | 0.9966 | 0.97 | 0.5-250 | 0.95 |
|  | y = 792318x + 4126980 | 0.9984 |  | 0.5-250 | 1.76 |
| Atrazine | y = 159865x - 47618.2 | 0.9997 | 0.79 | 0.5-250 | 2.02 |
|  | y = 202545x + 326109 | 0.9992 |  | 0.5-250 | 0.21 |
| Azaconazole | y = 199729x - 265057 | 0.9984 | 0.74 | 0.5-250 | 2.46 |
|  | y = 268372x + 133688 | 0.9995 |  | 0.5-250 | 1.94 |
| Azamethiphos | y = 48573.9x - 105269 | 0.9989 | 0.50 | 0.5-250 | 1.60 |
|  | y = 98024.9x - 20835.8 | 0.9999 |  | 0.5-250 | 1.76 |
| Azoxystrobin | y = 572204x - 228272 | 0.9996 | 0.77 | 0.5-100 | 2.97 |
|  | y = 743784x + 71347.5 | 0.9997 |  | 0.5-100 | 0.51 |
| Benalaxyl | y = 182570x - 428237 | 0.9972 | 1.09 | 0.5-250 | 2.98 |
|  | y = 167645x - 185188 | 0.9997 |  | 0.5-250 | 1.46 |
| Bendiocarb | y = 8804.12x + 12829.8 | 0.9991 | 0.09 | 0.5-250 | 2.81 |
|  | y = 96648.3x + 211203 | 0.9991 |  | 0.5-250 | 0.84 |
| Bitertanol | y = 5700.48x + 13572.7 | 0.9986 | 1.09 | 2-250 | 1.41 |
|  | y = 5224.81x + 10194.4 | 0.9993 |  | 0.5-250 | 1.59 |
| Boscalid | y = 86100.6x + 37543.6 | 0.9993 | 0.90 | 0.5-250 | 2.00 |
|  | y = 95363.2x + 84231.6 | 0.9994 |  | 0.5-250 | 0.91 |
| Bromuconazole | y = 30733.1x - 38271 | 0.9990 | 0.91 | 0.5-250 | 0.29 |
|  | y = 33592.1x + 11743.8 | 0.9997 |  | 0.5-250 | 0.74 |
| Buprofezin | y = 287755x + 549777 | 0.9993 | 0.95 | 0.5-250 | 0.45 |
|  | y = 301316x + 498285 | 0.9993 |  | 0.5-250 | 0.85 |
| Butachlor | y = 164873x + 873302 | 0.9969 | 1.03 | 0.5-250 | 3.46 |
|  | y = 160435x + 619420 | 0.9981 |  | 0.5-250 | 0.79 |
| Cadusafos | y = 292388x + 301627 | 0.9998 | 0.93 | 0.5-250 | 1.58 |
|  | y = 315842x + 527213 | 0.9994 |  | 0.5-250 | 0.62 |
| Carbendazim | y = 92461.1x + 39420.2 | 0.9999 | 0.28 | 0.5-250 | 0.21 |
|  | y = 334448x + 280153 | 0.9993 |  | 0.5-250 | 1.21 |
| Carbofuran | y = 31461.7x - 15017.3 | 0.9996 | 0.11 | 0.5-250 | 4.87 |
|  | y = 288008x + 235300 | 0.9992 |  | 0.5-250 | 0.64 |
| Chlorantraniliprole | y = 55777.1x - 58263.1 | 0.9992 | 0.95 | 0.5-250 | 3.09 |
|  | y = 58737.3x + 167462 | 0.9993 |  | 0.5-250 | 0.57 |
| Chlorpyrifos | y = 694265x + 1093800 | 0.9989 | 0.90 | 0.5-100 | 1.79 |
|  | y = 771286x + 1044540 | 0.9988 |  | 0.5-100 | 1.25 |
| Chlortoluron | y = 129252x + 56149.9 | 0.9991 | 0.49 | 0.5-250 | 4.86 |
|  | y = 265439x + 701160 | 0.9989 |  | 0.5-250 | 1.68 |
| Clothianidin | y = 4191.3x - 7464.83 | 0.9994 | 0.07 | 0.5-250 | 1.89 |
|  | y = 63358x + 45132.3 | 0.9998 |  | 0.5-250 | 1.08 |
| Coumaphos | y = 204407x + 82472.9 | 0.9995 | 0.98 | 0.5-250 | 2.26 |
|  | y = 209361x + 639593 | 0.9992 |  | 0.5-250 | 0.86 |
| Cyanophenphos | y = 37550.2x + 21263.3 | 0.9995 | 1.02 | 0.5-250 | 3.69 |
|  | y = 36866.1x + 100916 | 0.9992 |  | 0.5-250 | 0.84 |
| Cyazofamid` | y = 52971.5x - 19926.8 | 0.9972 | 0.67 | 0.5-250 | 0.14 |
|  | y = 79298.7x + 188035 | 0.9991 |  | 0.5-250 | 0.52 |
| Cyproconazole | y = 80799.6x - 12030.8 | 0.9990 | 0.82 | 0.5-250 | 2.86 |
|  | y = 98745.4x + 278702 | 0.9993 |  | 0.5-250 | 1.23 |
| Cyprodinil | y = 135107x - 147109 | 0.9992 | 0.99 | 0.5-250 | 4.37 |
|  | y = 135850x + 86987.3 | 0.9995 |  | 0.5-250 | 1.37 |
| Dicofol | y = 8470.28x + 3363.17 | 0.9990 | 1.00 | 0.5-100 | 0.44 |
|  | y = 8500.96x - 2216.31 | 0.9980 |  | 0.5-100 | 1.61 |
| Dicrotophos | y = 6052.9x - 7044.4 | 0.9994 | 0.11 | 0.5-250 | 2.56 |
|  | y = 53568.7x - 4127.35 | 0.9998 |  | 0.5-250 | 0.43 |
| Difenoconazole | y = 151404x - 160333 | 0.9993 | 1.10 | 0.5-250 | 4.96 |
|  | y = 137817x - 48437.3 | 0.9998 |  | 0.5-250 | 2.00 |
| Dimethoate | y = 55561.9x - 76019.6 | 0.9997 | 0.26 | 0.5-250 | 4.06 |
|  | y = 213398x - 125164 | 0.9999 |  | 0.5-250 | 1.49 |
| Dimethomorph | y = 198049x + 66222.5 | 0.9996 | 1.69 | 0.5-250 | 1.33 |
|  | y = 116953x + 178724 | 0.9993 |  | 0.5-250 | 1.05 |
| dimethyl phthalate | y = 80844.9x + 1542090 | 0.9987 | 0.69 | 0.5-250 | 1.25 |
|  | y = 117004x + 1609990 | 0.9995 |  | 0.5-250 | 0.42 |
| Diniconazole | y = 44818.6x + 16724.2 | 0.9996 | 0.99 | 0.5-250 | 1.24 |
|  | y = 45491.1x + 94764.9 | 0.9990 |  | 0.5-250 | 0.89 |
| Dinotefuran | y = 36411.1x + 9771.39 | 0.9999 | 0.61 | 0.5-250 | 1.96 |
|  | y = 59625.9x + 44005.2 | 0.9997 |  | 0.5-250 | 1.02 |
| Emamectin B1 Benzoate | y = 329491x - 326278 | 0.9997 | 0.99 | 0.5-250 | 1.36 |
|  | y = 331424x - 70644.4 | 0.9992 |  | 0.5-250 | 0.38 |
| Epoxiconazole | y = 88277.2x - 173319 | 0.9990 | 0.71 | 0.5-250 | 1.62 |
|  | y = 124881x - 43441 | 0.9992 |  | 0.5-250 | 0.24 |
| Ethiofencarb | y = 437184x - 168645 | 0.9998 | 0.31 | 0.5-50 | 3.80 |
|  | y = 1422230x + 597334 | 0.9961 |  | 0.5-50 | 1.36 |
| Etofenprox | y = 238688x + 154177 | 0.9999 | 1.04 | 0.5-250 | 4.02 |
|  | y = 229507x + 101162 | 0.9995 |  | 0.5-250 | 0.31 |
| Etoxazole | y = 721889x + 417628 | 0.9996 | 1.12 | 0.5-100 | 3.01 |
|  | y = 643096x + 534898 | 0.9997 |  | 0.5-100 | 0.58 |
| Fenamiphos | y = 398036x + 863010 | 0.9986 | 1.04 | 0.5-200 | 1.60 |
|  | y = 381984x + 1564290 | 0.9990 |  | 0.5-250 | 1.51 |
| Fenamiphos sulfoxide | y = 49047.9x - 115696 | 0.9990 | 0.35 | 0.5-250 | 2.03 |
|  | y = 142107x + 36275.4 | 0.9999 |  | 0.5-250 | 1.56 |
| Fenbuconazole | y = 63950.8x - 131427 | 0.9981 | 0.83 | 0.5-250 | 1.57 |
|  | y = 77275.9x - 195151 | 0.9990 |  | 0.5-250 | 1.25 |
| Fenpropathrin | y = 14492.2x + 467.617 | 0.9986 | 1.47 | 0.5-100 | 1.66 |
|  | y = 9836.11x + 22907.2 | 0.9966 |  | 0.5-100 | 1.24 |
| Fipronil | y = 8287.45x + 17507.4 | 0.9992 | 0.77 | 0.5-250 | 3.64 |
|  | y = 10769.2x - 17592.3 | 0.9991 |  | 0.5-250 | 0.62 |
| Fipronil-sulfone | y = 145647x + 60404.4 | 0.9996 | 0.79 | 0.5-250 | 3.36 |
|  | y = 183973x - 50212.4 | 0.9994 |  | 0.5-250 | 1.64 |
| Fludioxonil | y = 28185.8x + 115509 | 0.9990 | 0.91 | 0.5-250 | 1.27 |
|  | y = 30914.8x + 86835 | 0.9992 |  | 0.5-250 | 0.19 |
| Fluopyram | y = 185550x + 72511.1 | 0.9996 | 0.86 | 0.5-250 | 1.35 |
|  | y = 216694x - 397443 | 0.9993 |  | 0.5-250 | 0.44 |
| Fluoxastrobin | y = 324227x + 622224 | 0.9995 | 0.99 | 0.5-250 | 2.25 |
|  | y = 326343x + 835206 | 0.9994 |  | 0.5-250 | 2.00 |
| Flusilazole | y = 147297x - 184194 | 0.9987 | 0.93 | 0.5-250 | 2.61 |
|  | y = 157601x - 219056 | 0.9993 |  | 0.5-250 | 1.09 |
| Flutolanil | y = 417711x + 437139 | 0.9987 | 0.69 | 0.5-100 | 2.23 |
|  | y = 604339x - 162788 | 0.9997 |  | 0.5-100 | 1.70 |
| Flutriafol | y = 25395.1x - 15816.5 | 0.9998 | 0.56 | 0.5-250 | 4.68 |
|  | y = 45577x - 12136.6 | 0.9995 |  | 0.5-250 | 1.93 |
| Forchlorfenuron | y = 77610.7x - 29501.3 | 0.9996 | 0.88 | 0.5-250 | 1.61 |
|  | y = 87807.5x + 280399 | 0.9992 |  | 0.5-250 | 1.86 |
| Hexaconazole | y = 72510.7x - 60395.7 | 0.9988 | 1.08 | 0.5-250 | 1.81 |
|  | y = 66860.6x + 53485.5 | 0.9995 |  | 0.5-250 | 1.26 |
| Hexaflumuron | y = 348020x + 1866980 | 0.9966 | 1.00 | 0.5-250 | 3.63 |
|  | y = 349339x + 1178900 | 0.9992 |  | 0.5-250 | 1.33 |
| Hexazinone | y = 257178x + 27587.2 | 0.9999 | 0.36 | 0.5-100 | 2.79 |
|  | y = 717261x + 309539 | 0.9998 |  | 0.5-100 | 0.97 |
| Imazalil | y = 92635x - 18131.9 | 0.9997 | 0.91 | 0.5-250 | 3.33 |
|  | y = 101466x + 89024.5 | 0.9996 |  | 0.5-250 | 1.26 |
| Indoxacarb | y = 18750.8x + 58747.2 | 0.9995 | 0.97 | 0.5-250 | 2.66 |
|  | y = 19396x + 47574.7 | 0.9991 |  | 0.5-250 | 1.90 |
| Ipconazole | y = 143192x + 256315 | 0.9996 | 0.83 | 0.5-250 | 3.74 |
|  | y = 173211x + 311393 | 0.9992 |  | 0.5-250 | 0.90 |
| Isoprocarb | y = 115998x + 8403.88 | 0.9996 | 0.54 | 0.5-250 | 1.32 |
|  | y = 213432x - 324303 | 0.9991 |  | 0.5-250 | 0.62 |
| Kresoxim-methyl | y = 51812.3x + 23509.4 | 0.9996 | 0.85 | 0.5-100 | 2.60 |
|  | y = 60608.7x - 32216 | 0.9997 |  | 0.5-100 | 0.90 |
| Lufenuron | y = 10839.8x + 115840 | 0.9909 | 0.82 | 0.5-250 | 4.06 |
|  | y = 13251.2x + 78340.9 | 0.9980 |  | 0.5-250 | 0.76 |
| Malaoxon | y = 162481x - 52955.9 | 0.9998 | 0.19 | 0.5-100 | 4.45 |
|  | y = 839041x + 501694 | 0.9998 |  | 0.5-100 | 0.79 |
| Malathion | y = 116940x + 318363 | 0.9989 | 0.89 | 0.5-250 | 4.06 |
|  | y = 132104x + 396108 | 0.9994 |  | 0.5-250 | 1.36 |
| Mefenoxam | y = 496639x + 161067 | 0.9999 | 0.77 | 0.5-100 | 0.99 |
|  | y = 646361x + 696020 | 0.9995 |  | 0.5-100 | 1.24 |
| Mepronil | y = 2581250x + 554322 | 0.9980 | 0.81 | 0.5-50 | 1.35 |
|  | y = 3175610x + 459022 | 0.9997 |  | 0.5-50 | 0.67 |
| Metalaxyl | y = 480990x + 331895 | 0.9996 | 0.67 | 0.5-100 | 0.85 |
|  | y = 718117x + 142354 | 0.9999 |  | 0.5-100 | 1.77 |
| Methidathion | y = 29884.5x + 21529.7 | 0.9997 | 0.77 | 0.5-250 | 2.01 |
|  | y = 38779.7x + 8462.38 | 0.9997 |  | 0.5-250 | 0.61 |
| Methomyl | y = 36840.1x - 15660.8 | 0.9999 | 0.52 | 0.5-250 | 3.22 |
|  | y = 70897.1x - 100837 | 0.9996 |  | 0.5-250 | 0.83 |
| Methoprene | y = 23813.6x + 5273.44 | 0.9998 | 1.00 | 0.5-250 | 3.24 |
|  | y = 23855.3x + 1560.05 | 0.9994 |  | 0.5-250 | 1.17 |
| Methoxyfenozide | y = 74037.9x + 50391 | 0.9996 | 1.00 | 0.5-250 | 0.92 |
|  | y = 74074.9x + 70085.8 | 0.9992 |  | 0.5-250 | 1.52 |
| Mevinphos | y = 18168.3x - 8800.92 | 0.9997 | 0.27 | 0.5-250 | 1.12 |
|  | y = 66496.0x - 64412.2 | 0.9995 |  | 0.5-250 | 1.88 |
| Monocrotophos | y = 4887.02x - 11196.5 | 0.9993 | 0.14 | 0.5-250 | 0.54 |
|  | y = 34513.8x + 11875.1 | 0.9997 |  | 0.5-250 | 0.19 |
| Myclobutanil | y = 62644.9x + 121915 | 0.9991 | 0.93 | 0.5-250 | 4.44 |
|  | y = 67118.4x + 142114 | 0.9997 |  | 0.5-250 | 1.92 |
| Omethoate | y = 69087.5x - 45022.1 | 0.9995 | 0.77 | 0.5-250 | 2.94 |
|  | y = 89900.3x - 116146 | 0.9998 |  | 0.5-250 | 1.37 |
| Paclobutrazol | y = 148119x + 347080 | 0.9991 | 0.85 | 0.5-250 | 1.77 |
|  | y = 174702x + 90474.6 | 0.9990 |  | 0.5-250 | 0.68 |
| Penconazole | y = 94382x + 94564.1 | 0.9997 | 1.03 | 0.5-250 | 4.39 |
|  | y = 91994.6x + 140739 | 0.9991 |  | 0.5-250 | 0.51 |
| Phenthoate | y = 122915x + 445727 | 0.9993 | 0.91 | 0.5-250 | 0.28 |
|  | y = 135636x + 493223 | 0.9994 |  | 0.5-250 | 0.52 |
| Phorate | y = 35824.2x - 65287.8 | 0.9960 | 0.98 | 0.5-250 | 1.52 |
|  | y = 36585.5x + 41822.3 | 0.9990 |  | 0.5-250 | 0.78 |
| Phorate sulfone | y = 24175.5x + 16649 | 0.9997 | 0.44 | 0.5-100 | 3.19 |
|  | y = 55229x + 44723.2 | 0.9995 |  | 0.5-100 | 1.97 |
| Phosalone | y = 1261810x + 863112 | 0.9991 | 0.98 | 0.5-50 | 4.80 |
|  | y = 1281220x + 826217 | 0.9988 |  | 0.5-50 | 0.16 |
| Phosfolan | y = 160587x - 74121 | 0.9993 | 0.46 | 0.5-250 | 0.95 |
|  | y = 351640x + 793462 | 0.9993 |  | 0.5-250 | 1.99 |
| Phosmet | y = 144162x - 113845 | 0.9989 | 0.77 | 0.5-250 | 0.85 |
|  | y = 187056x + 250378 | 0.9999 |  | 0.5-250 | 1.43 |
| Phosphamidon | y = 94304.2x - 66730.5 | 0.9994 | 0.73 | 0.5-250 | 4.09 |
|  | y = 128857x - 145106 | 0.9998 |  | 0.5-250 | 1.88 |
| Piperonyl butoxide | y = 329344x + 360335 | 0.9990 | 1.00 | 0.5-250 | 2.83 |
|  | y = 328481x + 511020 | 0.9992 |  | 0.5-250 | 1.87 |
| Pirimicarb | y = 245504x - 693462 | 0.9967 | 0.73 | 0.5-250 | 3.71 |
|  | y = 335094x + 1196200 | 0.9990 |  | 0.5-250 | 0.18 |
| Pirimiphos-methyl | y = 317437x - 75658.4 | 0.9992 | 0.98 | 0.5-250 | 0.30 |
|  | y = 322697x + 282327 | 0.9994 |  | 0.5-250 | 0.22 |
| Prochloraz | y = 159927x + 253425 | 0.9998 | 0.87 | 0.5-250 | 4.29 |
|  | y = 184363x - 38432 | 0.9993 |  | 0.5-250 | 0.81 |
| Profenofos | y = 99916.9x + 166941 | 0.9988 | 0.89 | 0.5-250 | 2.49 |
|  | y = 112112x + 241932 | 0.9991 |  | 0.5-250 | 1.33 |
| Propamocarb | y = 206886x - 424204 | 0.9991 | 0.78 | 0.5-250 | 3.81 |
|  | y = 264294x - 325221 | 0.9996 |  | 0.5-250 | 1.38 |
| Propiconazole | y = 93899.9x + 223078 | 0.9995 | 0.92 | 0.5-250 | 1.50 |
|  | y = 102586x + 216541 | 0.9994 |  | 0.5-250 | 0.66 |
| Prothiophos | y = 38485.3x - 15997.6 | 0.9970 | 0.79 | 0.5-250 | 3.41 |
|  | y = 48650.7x - 3180.76 | 0.9980 |  | 1-250 | 1.97 |
| Pyraclostrobine | y = 179702x + 28684.5 | 0.9997 | 0.96 | 0.5-250 | 0.66 |
|  | y = 186375x + 307622 | 0.9992 |  | 0.5-250 | 1.53 |
| Pyridaben | y = 258857x - 213951 | 0.9996 | 0.99 | 0.5-250 | 4.50 |
|  | y = 260273x + 50248.3 | 0.9998 |  | 0.5-250 | 0.53 |
| Pyridafenthion | y = 205334x + 315191 | 0.9994 | 1.01 | 0.5-250 | 4.22 |
|  | y = 204024x + 191747 | 0.9994 |  | 0.5-250 | 0.63 |
| Pyrimethanil | y = 59044x + 84816.6 | 0.9998 | 0.90 | 0.5-250 | 0.90 |
|  | y = 65358.3x + 120705 | 0.9994 |  | 0.5-250 | 1.69 |
| Pyrimitate | y = 946392x - 114738 | 0.9997 | 1.13 | 0.5-100 | 4.93 |
|  | y = 835352x + 732844 | 0.9995 |  | 0.5-100 | 1.12 |
| Pyriproxyfen | y = 345652x + 1235490 | 0.9979 | 1.02 | 0.5-250 | 1.79 |
|  | y = 339296x + 923521 | 0.9990 |  | 0.5-250 | 1.56 |
| Pyrisoxazole | y = 11475.4x + 31609.4 | 0.9990 | 1.10 | 0.5-250 | 0.21 |
|  | y = 10389.2x + 31131 | 0.9991 |  | 0.5-250 | 0.30 |
| Quizalofop-p-ethyl | y = 318146x - 109711 | 0.9985 | 0.96 | 0.5-250 | 1.14 |
|  | y = 331894x + 248432 | 0.9993 |  | 0.5-250 | 1.54 |
| Simeconazole | y = 146256x - 122632 | 0.9987 | 0.74 | 0.5-250 | 4.99 |
|  | y = 196491x - 29091.1 | 0.9995 |  | 0.5-250 | 1.96 |
| Spirodiclofen | y = 41589.2x + 11723.2 | 0.9995 | 0.90 | 0.5-250 | 1.13 |
|  | y = 46401.8x + 13914.9 | 0.9998 |  | 0.5-250 | 0.49 |
| Sulfotep | y = 200924x + 281872 | 0.9993 | 0.89 | 0.5-250 | 2.00 |
|  | y = 225425x + 566533 | 0.9994 |  | 0.5-250 | 1.27 |
| Sulprofos | y = 132646x - 309135 | 0.9951 | 0.96 | 0.5-250 | 4.43 |
|  | y = 138275x - 150856 | 0.9992 |  | 0.5-250 | 1.84 |
| Tebuconazole | y = 160454x + 69803.5 | 0.9997 | 1.00 | 0.5-250 | 4.26 |
|  | y = 160048x + 13926.9 | 0.9998 |  | 0.5-250 | 1.26 |
| Tebufenozide | y = 52657.9x + 143155 | 0.9990 | 0.89 | 0.5-250 | 2.00 |
|  | y = 59088.7x + 32806.8 | 0.9999 |  | 0.5-250 | 1.02 |
| Tebufenpyrad | y = 103300x - 66111.9 | 0.9995 | 0.97 | 0.5-250 | 1.21 |
|  | y = 106275x - 191860 | 0.9991 |  | 0.5-250 | 1.57 |
| Temephos | y = 346334x + 1488440 | 0.9968 | 1.00 | 0.5-250 | 4.64 |
|  | y = 347483x + 1162690 | 0.9994 |  | 0.5-250 | 0.71 |
| Terbufos | y = 8883.82x + 32390.8 | 0.9988 | 1.04 | 0.5-250 | 3.40 |
|  | y = 8518.68x + 33819.1 | 0.9992 |  | 0.5-250 | 1.92 |
| Tetrachlorvinphos | y = 35298.1x + 131313 | 0.9990 | 0.94 | 0.5-250 | 4.90 |
|  | y = 37520.1x + 71579.9 | 0.9998 |  | 0.5-250 | 0.68 |
| Tetraconazole | y = 137028x + 172736 | 0.9982 | 0.80 | 0.5-100 | 4.17 |
|  | y = 172206x + 18684.8 | 0.9996 |  | 0.5-100 | 1.01 |
| Thiacloprid | y = 233945x - 260588 | 0.9996 | 0.56 | 0.5-250 | 4.65 |
|  | y = 416872x + 1377520 | 0.9993 |  | 0.5-250 | 2.00 |
| Thiamethoxam | y = 30381.4x - 2341.3 | 0.9993 | 0.22 | 0.5-250 | 2.39 |
|  | y = 139911x + 4966.57 | 0.9999 |  | 0.5-250 | 1.81 |
| Thiophanate-methyl | y = 91404.6x - 408162 | 0.9941 | 0.27 | 0.5-200 | 0.33 |
|  | y = 338599x + 515079 | 0.9995 |  | 0.5-250 | 1.40 |
| Tolfenpyrad | y = 98048.7x + 234257 | 0.9990 | 1.02 | 0.5-250 | 1.00 |
|  | y = 96485.1x + 162015 | 0.9994 |  | 0.5-250 | 0.99 |
| Triadimefon | y = 46903.4x + 94704.5 | 0.9993 | 0.82 | 0.5-250 | 0.30 |
|  | y = 57301.5x + 90066.9 | 0.9994 |  | 0.5-250 | 1.33 |
| Triadimenol | y = 28068.9x - 29871.8 | 0.9994 | 1.01 | 0.5-250 | 0.40 |
|  | y = 27795.9x + 4696.08 | 0.9999 |  | 0.5-250 | 1.77 |
| Tricyclazole | y = 76085.3x + 63210.7 | 0.9994 | 0.32 | 0.5-250 | 1.89 |
|  | y = 234231x + 317911 | 0.9995 |  | 0.5-250 | 1.84 |
| Trifloxystrobin | y = 446759x + 1713820 | 0.9923 | 1.24 | 0.5-200 | 4.31 |
|  | y = 361172x + 1671560 | 0.9984 |  | 0.5-250 | 0.38 |
| Triticonazole | y = 73657.5x - 135635 | 0.9992 | 0.73 | 0.5-250 | 3.29 |
|  | y = 100738x - 76418.6 | 0.9994 |  | 0.5-250 | 1.78 |
| Uniconazole | y = 91471.1x - 43202.9 | 0.9993 | 0.86 | 0.5-250 | 1.33 |
|  | y = 106013x + 353989 | 0.9992 |  | 0.5-250 | 1.52 |
| Vitavax | y = 1313380x - 153222 | 0.9993 | 0.40 | 0.5-50 | 3.76 |
|  | y = 3312810x - 605463 | 0.9996 |  | 0.5-50 | 1.81 |

^1^ Calibration curves are expressed as regression lines (y = a x + b), where y is the peak area and x is the concentration (μg/L), a is the slope, b is the intercept and R is the correlation coefficient.

^2^ The value of slope ratio is calculated by the slope of the matrix-matched calibration curve to the slope of the standard calibration curve in solvent.

^3^ The LOD was the three times of the singal to noise ratio.

^4^ The first calibration curve for each pesticide represents the matrix-matched calibration curve.

^5^ The second calibration curve for each pesticide represents the standard calibration curve in solvent.

Table S5 The method validation of the linearity, matrix effect and LODs of each pesticide in PRA-SD.

| Pesticides | ^1^y=a x+b | R | ^2^Sloperatio | Linear range, | ^3^LOD, |
| --- | --- | --- | --- | --- | --- |
|  |  |  |  | μg/L | μg/kg |
| 3-Hydroxy-carbofuran | ^4^y = 3297.34x - 8335.14 | 0.9983 | 0.15 | 1-250 | 1.04 |
|  | ^5^y = 22478.1x - 8901.3 | 0.9997 |  | 0.5-250 | 0.28 |
| Abamectin | y = 4909.15x - 7450.63 | 0.9952 | 1.40 | 1-200 | 1.80 |
|  | y = 3495.24x + 1957.13 | 0.9999 |  | 1-250 | 0.25 |
| Acephate | y = 88053.8x - 68877.9 | 0.9998 | 0.91 | 0.5-250 | 4.19 |
|  | y = 96800.2x - 3819.54 | 0.9996 |  | 0.5-250 | 1.59 |
| Acetamiprid | y = 36884.7x - 13556.4 | 0.9974 | 0.17 | 0.5-250 | 4.49 |
|  | y = 223248x + 47946 | 0.9996 |  | 0.5-250 | 1.81 |
| Alachlor | y = 755891x + 1964990 | 0.9989 | 0.95 | 0.5-250 | 4.09 |
|  | y = 792318x + 4126980 | 0.9984 |  | 0.5-250 | 1.76 |
| Atrazine | y = 147952x + 50045.5 | 0.9999 | 0.73 | 0.5-250 | 3.58 |
|  | y = 202545x + 326109 | 0.9992 |  | 0.5-250 | 0.21 |
| Azaconazole | y = 194316x - 80347.9 | 0.9995 | 0.72 | 0.5-250 | 4.60 |
|  | y = 268372x + 133688 | 0.9995 |  | 0.5-250 | 1.94 |
| Azamethiphos | y = 39255x + 43553.3 | 0.9993 | 0.40 | 0.5-250 | 1.08 |
|  | y = 98024.9x - 20835.8 | 0.9999 |  | 0.5-250 | 1.76 |
| Azoxystrobin | y = 544321x - 102813 | 0.9999 | 0.73 | 0.5-100 | 2.87 |
|  | y = 743784x + 71347.5 | 0.9997 |  | 0.5-100 | 0.51 |
| Benalaxyl | y = 169756x - 135011 | 0.9994 | 1.01 | 0.5-250 | 0.96 |
|  | y = 167645x - 185188 | 0.9997 |  | 0.5-250 | 1.46 |
| Bendiocarb | y = 8717.22x - 11002.3 | 0.9998 | 0.09 | 1-250 | 2.95 |
|  | y = 96648.3x + 211203 | 0.9991 |  | 0.5-250 | 0.84 |
| Bitertanol | y = 6826.55x - 9416.96 | 0.9995 | 1.31 | 1-250 | 1.51 |
|  | y = 5224.81x + 10194.4 | 0.9993 |  | 0.5-250 | 1.59 |
| Boscalid | y = 87895.7x + 169696 | 0.9997 | 0.92 | 0.5-250 | 2.27 |
|  | y = 95363.2x + 84231.6 | 0.9994 |  | 0.5-250 | 0.91 |
| Bromuconazole | y = 35968.5x - 108364 | 0.9979 | 1.07 | 0.5-250 | 0.56 |
|  | y = 33592.1x + 11743.8 | 0.9997 |  | 0.5-250 | 0.74 |
| Buprofezin | y = 294476x + 524644 | 0.9989 | 0.98 | 0.5-250 | 1.00 |
|  | y = 301316x + 498285 | 0.9993 |  | 0.5-250 | 0.85 |
| Butachlor | y = 168323x + 904832 | 0.9964 | 1.05 | 0.5-250 | 3.36 |
|  | y = 160435x + 619420 | 0.9981 |  | 0.5-250 | 0.79 |
| Cadusafos | y = 306565x + 53757 | 0.9997 | 0.97 | 0.5-250 | 0.63 |
|  | y = 315842x + 527213 | 0.9994 |  | 0.5-250 | 0.62 |
| Carbendazim | y = 107877x - 118751 | 0.9993 | 0.32 | 0.5-250 | 3.18 |
|  | y = 334448x + 280153 | 0.9993 |  | 0.5-250 | 1.21 |
| Carbofuran | y = 30450.2x + 3085.31 | 0.9994 | 0.11 | 0.5-250 | 2.91 |
|  | y = 288008x + 235300 | 0.9992 |  | 0.5-250 | 0.64 |
| Chlorantraniliprole | y = 56789.9x + 30346.2 | 0.9998 | 0.97 | 0.5-250 | 0.22 |
|  | y = 58737.3x + 167462 | 0.9993 |  | 0.5-250 | 0.57 |
| Chlorpyrifos | y = 694137x + 1425470 | 0.9970 | 0.90 | 0.5-100 | 2.61 |
|  | y = 771286x + 1044540 | 0.9988 |  | 0.5-100 | 1.25 |
| Chlortoluron | y = 116347x + 6634.01 | 0.9998 | 0.44 | 0.5-250 | 4.03 |
|  | y = 265439x + 701160 | 0.9989 |  | 0.5-250 | 1.68 |
| Clothianidin | y = 3704.74x + 297.281 | 0.9996 | 0.06 | 0.5-250 | 3.25 |
|  | y = 63358x + 45132.3 | 0.9998 |  | 0.5-250 | 1.08 |
| Coumaphos | y = 235073x - 335062 | 0.9988 | 1.12 | 0.5-250 | 3.02 |
|  | y = 209361x + 639593 | 0.9992 |  | 0.5-250 | 0.86 |
| Cyanophenphos | y = 37153.1x + 24638.8 | 0.9996 | 1.01 | 0.5-250 | 2.81 |
|  | y = 36866.1x + 100916 | 0.9992 |  | 0.5-250 | 0.84 |
| Cyazofamid` | y = 49345.2x + 81170 | 0.9993 | 0.62 | 0.5-250 | 0.14 |
|  | y = 79298.7x + 188035 | 0.9991 |  | 0.5-250 | 0.52 |
| Cyproconazole | y = 82928.9x + 127673 | 0.9992 | 0.84 | 0.5-250 | 3.18 |
|  | y = 98745.4x + 278702 | 0.9993 |  | 0.5-250 | 1.23 |
| Cyprodinil | y = 128356x - 183657 | 0.9989 | 0.94 | 0.5-250 | 2.13 |
|  | y = 135850x + 86987.3 | 0.9995 |  | 0.5-250 | 1.37 |
| Dicofol | y = 8036.92x + 10497.6 | 0.9977 | 0.95 | 0.5-100 | 0.80 |
|  | y = 8500.96x - 2216.31 | 0.9980 |  | 0.5-100 | 1.61 |
| Dicrotophos | y = 4803.77x + 7531.36 | 0.9995 | 0.09 | 01-250 | 1.02 |
|  | y = 53568.7x - 4127.35 | 0.9998 |  | 0.5-250 | 0.43 |
| Difenoconazole | y = 156544x - 151875 | 0.9989 | 1.14 | 0.5-250 | 4.14 |
|  | y = 137817x - 48437.3 | 0.9998 |  | 0.5-250 | 2.00 |
| Dimethoate | y = 46445.4x - 19364.5 | 0.9998 | 0.22 | 0.5-250 | 1.27 |
|  | y = 213398x - 125164 | 0.9999 |  | 0.5-250 | 1.49 |
| Dimethomorph | y = 200022x + 45484.4 | 0.9997 | 1.71 | 0.5-250 | 0.63 |
|  | y = 116953x + 178724 | 0.9993 |  | 0.5-250 | 1.05 |
| dimethyl phthalate | y = 66688.1x + 1944560 | 0.9975 | 0.57 | 0.5-250 | 4.90 |
|  | y = 117004x + 1609990 | 0.9995 |  | 0.5-250 | 0.42 |
| Diniconazole | y = 44933.7x + 29084.1 | 0.9996 | 0.99 | 0.5-250 | 2.57 |
|  | y = 45491.1x + 94764.9 | 0.9990 |  | 0.5-250 | 0.89 |
| Dinotefuran | y = 37893.5x - 9763.21 | 0.9999 | 0.64 | 0.5-250 | 1.49 |
|  | y = 59625.9x + 44005.2 | 0.9997 |  | 0.5-250 | 1.02 |
| Emamectin B1 Benzoate | y = 328040x + 68457.1 | 0.9993 | 0.99 | 0.5-250 | 2.53 |
|  | y = 331424x - 70644.4 | 0.9992 |  | 0.5-250 | 0.38 |
| Epoxiconazole | y = 85742.6x + 76086.1 | 0.9996 | 0.69 | 0.5-250 | 2.30 |
|  | y = 124881x - 43441 | 0.9992 |  | 0.5-250 | 0.24 |
| Ethiofencarb | y = 325973x - 52221.1 | 0.9997 | 0.23 | 0.5-50 | 2.57 |
|  | y = 1422230x + 597334 | 0.9961 |  | 0.5-50 | 1.36 |
| Etofenprox | y = 184305x + 333989 | 0.9956 | 0.80 | 0.5-250 | 2.55 |
|  | y = 229507x + 101162 | 0.9995 |  | 0.5-250 | 0.31 |
| Etoxazole | y = 729623x + 506602 | 0.9979 | 1.13 | 0.5-100 | 0.99 |
|  | y = 643096x + 534898 | 0.9997 |  | 0.5-100 | 0.58 |
| Fenamiphos | y = 405993x + 489941 | 0.9986 | 1.06 | 0.5-250 | 1.86 |
|  | y = 381984x + 1564290 | 0.9990 |  | 0.5-250 | 1.51 |
| Fenamiphos sulfoxide | y = 44727x - 37235.8 | 0.9990 | 0.31 | 0.5-250 | 4.00 |
|  | y = 142107x + 36275.4 | 0.9999 |  | 0.5-250 | 1.56 |
| Fenbuconazole | y = 59041.7x + 68455.6 | 0.9984 | 0.76 | 0.5-250 | 2.28 |
|  | y = 77275.9x - 195151 | 0.9990 |  | 0.5-250 | 1.25 |
| Fenpropathrin | y = 12427.6x + 13630.5 | 0.9972 | 1.26 | 0.5-100 | 3.43 |
|  | y = 9836.11x + 22907.2 | 0.9966 |  | 0.5-100 | 1.24 |
| Fipronil | y = 7484.28x + 15644 | 0.9992 | 0.69 | 0.5-250 | 3.11 |
|  | y = 10769.2x - 17592.3 | 0.9991 |  | 0.5-250 | 0.62 |
| Fipronil-sulfone | y = 137357x + 120495 | 0.9992 | 0.75 | 0.5-250 | 4.38 |
|  | y = 183973x - 50212.4 | 0.9994 |  | 0.5-250 | 1.64 |
| Fludioxonil | y = 30028.3x + 79197.4 | 0.9993 | 0.97 | 0.5-250 | 2.84 |
|  | y = 30914.8x + 86835 | 0.9992 |  | 0.5-250 | 0.19 |
| Fluopyram | y = 182253x + 67831.1 | 0.9996 | 0.84 | 0.5-250 | 3.52 |
|  | y = 216694x - 397443 | 0.9993 |  | 0.5-250 | 0.44 |
| Fluoxastrobin | y = 323622x + 912112 | 0.9983 | 0.99 | 0.5-250 | 0.57 |
|  | y = 326343x + 835206 | 0.9994 |  | 0.5-250 | 2.00 |
| Flusilazole | y = 140747x + 179134 | 0.9978 | 0.89 | 0.5-250 | 4.35 |
|  | y = 157601x - 219056 | 0.9993 |  | 0.5-250 | 1.09 |
| Flutolanil | y = 395373x + 386028 | 0.9987 | 0.65 | 0.5-100 | 1.28 |
|  | y = 604339x - 162788 | 0.9997 |  | 0.5-100 | 1.70 |
| Flutriafol | y = 26738x - 32494.2 | 0.9994 | 0.59 | 0.5-250 | 2.75 |
|  | y = 45577x - 12136.6 | 0.9995 |  | 0.5-250 | 1.93 |
| Forchlorfenuron | y = 68788.7x + 54506.3 | 0.9992 | 0.78 | 0.5-250 | 2.86 |
|  | y = 87807.5x + 280399 | 0.9992 |  | 0.5-250 | 1.86 |
| Hexaconazole | y = 72821.4x - 25880.7 | 0.9988 | 1.09 | 0.5-250 | 4.98 |
|  | y = 66860.6x + 53485.5 | 0.9995 |  | 0.5-250 | 1.26 |
| Hexaflumuron | y = 353840x + 1747680 | 0.9959 | 1.01 | 0.5-250 | 3.65 |
|  | y = 349339x + 1178920 | 0.9992 |  | 0.5-250 | 1.33 |
| Hexazinone | y = 252632x - 71434.3 | 0.9998 | 0.35 | 0.5-100 | 4.73 |
|  | y = 717261x + 309539 | 0.9998 |  | 0.5-100 | 0.97 |
| Imazalil | y = 96181.1x - 59196.3 | 0.9996 | 0.95 | 0.5-250 | 1.58 |
|  | y = 101466x + 89024.5 | 0.9996 |  | 0.5-250 | 1.26 |
| Indoxacarb | y = 19948.7x + 11249.2 | 0.9996 | 1.03 | 0.5-250 | 3.31 |
|  | y = 19396x + 47574.7 | 0.9991 |  | 0.5-250 | 1.90 |
| Ipconazole | y = 143034x - 71598.9 | 0.9997 | 0.83 | 0.5-250 | 0.85 |
|  | y = 173211x + 311393 | 0.9992 |  | 0.5-250 | 0.90 |
| Isoprocarb | y = 109475x + 99848.1 | 0.9998 | 0.51 | 0.5-250 | 0.66 |
|  | y = 213432x - 324303 | 0.9991 |  | 0.5-250 | 0.62 |
| Kresoxim-methyl | y = 49818.9x + 27114.5 | 0.9988 | 0.82 | 0.5-100 | 2.20 |
|  | y = 60608.7x - 32216 | 0.9997 |  | 0.5-100 | 0.90 |
| Lufenuron | y = 11432.6x + 101169 | 0.9944 | 0.86 | 0.5-250 | 3.32 |
|  | y = 13251.2x + 78340.9 | 0.9980 |  | 0.5-250 | 0.76 |
| Malaoxon | y = 125091x - 30051.9 | 0.9999 | 0.15 | 0.5-100 | 2.37 |
|  | y = 839041x + 501694 | 0.9998 |  | 0.5-100 | 0.79 |
| Malathion | y = 109004x + 236896 | 0.9993 | 0.83 | 0.5-250 | 1.51 |
|  | y = 132104x + 396108 | 0.9994 |  | 0.5-250 | 1.36 |
| Mefenoxam | y = 505997x + 23714.8 | 0.9994 | 0.78 | 0.5-100 | 4.31 |
|  | y = 646361x + 696020 | 0.9995 |  | 0.5-100 | 1.24 |
| Mepronil | y = 2513610x + 135308 | 0.9998 | 0.79 | 0.5-50 | 4.67 |
|  | y = 3175610x + 459022 | 0.9997 |  | 0.5-50 | 0.67 |
| Metalaxyl | y = 494211x - 137987 | 0.9996 | 0.69 | 0.5-100 | 4.37 |
|  | y = 718117x + 142354 | 0.9999 |  | 0.5-100 | 1.77 |
| Methidathion | y = 29856.7x + 23421.9 | 0.9989 | 0.77 | 0.5-250 | 3.63 |
|  | y = 38779.7x + 8462.38 | 0.9997 |  | 0.5-250 | 0.61 |
| Methomyl | y = 36426x - 20175 | 0.9994 | 0.51 | 0.5-250 | 0.46 |
|  | y = 70897.1x - 100837 | 0.9996 |  | 0.5-250 | 0.83 |
| Methoprene | y = 20660.8x + 38587.9 | 0.9979 | 0.87 | 0.5-250 | 2.69 |
|  | y = 23855.3x + 1560.05 | 0.9994 |  | 0.5-250 | 1.17 |
| Methoxyfenozide | y = 70789.6x + 166941 | 0.9986 | 0.96 | 0.5-250 | 4.99 |
|  | y = 74074.9x + 70085.8 | 0.9992 |  | 0.5-250 | 1.52 |
| Mevinphos | y = 17953x - 12639.5 | 0.9993 | 0.27 | 0.5-250 | 2.53 |
|  | y = 66496x - 64412.2 | 0.9995 |  | 0.5-250 | 1.88 |
| Monocrotophos | y = 4353.83x - 8375.27 | 0.9983 | 0.13 | 0.5-250 | 1.42 |
|  | y = 34513.8x + 11875.1 | 0.9997 |  | 0.5-250 | 0.19 |
| Myclobutanil | y = 61130.8x + 170477 | 0.9992 | 0.91 | 0.5-250 | 1.60 |
|  | y = 67118.4x + 142114 | 0.9997 |  | 0.5-250 | 1.92 |
| Omethoate | y = 68099x - 56349 | 0.9996 | 0.76 | 0.5-250 | 1.94 |
|  | y = 89900.3x - 116146 | 0.9998 |  | 0.5-250 | 1.37 |
| Paclobutrazol | y = 140492x + 559591 | 0.9984 | 0.80 | 0.5-250 | 1.35 |
|  | y = 174702x + 90474.6 | 0.9990 |  | 0.5-250 | 0.68 |
| Penconazole | y = 93965.6x + 207469 | 0.9993 | 1.02 | 0.5-250 | 2.39 |
|  | y = 91994.6x + 140739 | 0.9991 |  | 0.5-250 | 0.51 |
| Phenthoate | y = 127125x + 356950 | 0.9989 | 0.94 | 0.5-250 | 0.62 |
|  | y = 135636x + 493223 | 0.9994 |  | 0.5-250 | 0.52 |
| Phorate | y = 31378.9x + 57043.2 | 0.9974 | 0.86 | 0.5-250 | 0.95 |
|  | y = 36585.5x + 41822.3 | 0.9990 |  | 0.5-250 | 0.78 |
| Phorate sulfone | y = 22602x + 39175.5 | 0.9980 | 0.41 | 0.5-100 | 2.93 |
|  | y = 55229x + 44723.2 | 0.9995 |  | 0.5-100 | 1.97 |
| Phosalone | y = 1270650x + 389778 | 0.9999 | 0.99 | 0.5-50 | 1.60 |
|  | y = 1281220x + 826217 | 0.9988 |  | 0.5-50 | 0.16 |
| Phosfolan | y = 152723x - 14076.6 | 0.9993 | 0.43 | 0.5-250 | 2.60 |
|  | y = 351640x + 793462 | 0.9993 |  | 0.5-250 | 1.99 |
| Phosmet | y = 144675x - 67708.7 | 0.9991 | 0.77 | 0.5-250 | 4.17 |
|  | y = 187056x + 250378 | 0.9999 |  | 0.5-250 | 1.43 |
| Phosphamidon | y = 85202.5x + 22887.6 | 0.9991 | 0.66 | 0.5-250 | 1.25 |
|  | y = 128857x - 145106 | 0.9998 |  | 0.5-250 | 1.88 |
| Piperonyl butoxide | y = 318914x + 722434 | 0.9982 | 0.97 | 0.5-250 | 2.43 |
|  | y = 328481x + 511020 | 0.9992 |  | 0.5-250 | 1.87 |
| Pirimicarb | y = 209312x - 21308.6 | 0.9999 | 0.62 | 0.5-250 | 0.64 |
|  | y = 335094x + 1196200 | 0.9990 |  | 0.5-250 | 0.18 |
| Pirimiphos-methyl | y = 310483x + 111150 | 0.9996 | 0.96 | 0.5-250 | 2.87 |
|  | y = 322697x + 282327 | 0.9994 |  | 0.5-250 | 0.22 |
| Prochloraz | y = 185858x - 6344.3 | 0.9996 | 1.01 | 0.5-250 | 3.31 |
|  | y = 184363x - 38432 | 0.9993 |  | 0.5-250 | 0.81 |
| Profenofos | y = 89385.6x + 430450 | 0.9976 | 0.80 | 0.5-250 | 3.44 |
|  | y = 112112x + 241932 | 0.9991 |  | 0.5-250 | 1.33 |
| Propamocarb | y = 203129x - 393308 | 0.9989 | 0.77 | 0.5-250 | 1.57 |
|  | y = 264294x - 325221 | 0.9996 |  | 0.5-250 | 1.38 |
| Propiconazole | y = 102713x + 72563.6 | 0.9995 | 1.00 | 0.5-250 | 1.82 |
|  | y = 102586x + 216541 | 0.9994 |  | 0.5-250 | 0.66 |
| Prothiophos | y = 43842.6x - 59514.3 | 0.9942 | 0.90 | 1-200 | 3.00 |
|  | y = 48650.7x - 3180.76 | 0.9980 |  | 1-250 | 1.97 |
| Pyraclostrobine | y = 194659x - 234359 | 0.9992 | 1.04 | 0.5-250 | 4.19 |
|  | y = 186375x + 307622 | 0.9992 |  | 0.5-250 | 1.53 |
| Pyridaben | y = 223022x + 445053 | 0.9981 | 0.86 | 0.5-250 | 0.66 |
|  | y = 260273x + 50248.3 | 0.9998 |  | 0.5-250 | 0.53 |
| Pyridafenthion | y = 211864x + 636197 | 0.9992 | 1.04 | 0.5-250 | 4.82 |
|  | y = 204024x + 191747 | 0.9994 |  | 0.5-250 | 0.63 |
| Pyrimethanil | y = 56169.8x + 115145 | 0.9988 | 0.86 | 0.5-250 | 4.50 |
|  | y = 65358.3x + 120705 | 0.9994 |  | 0.5-250 | 1.69 |
| Pyrimitate | y = 807327x + 1431160 | 0.9965 | 0.97 | 0.5-100 | 3.39 |
|  | y = 835352x + 732844 | 0.9995 |  | 0.5-100 | 1.12 |
| Pyriproxyfen | y = 343292x + 1177370 | 0.9965 | 1.01 | 0.5-250 | 4.93 |
|  | y = 339296x + 923521 | 0.9990 |  | 0.5-250 | 1.56 |
| Pyrisoxazole | y = 9361.13x + 79327.0 | 0.9959 | 0.90 | 1-250 | 2.51 |
|  | y = 10389.2x + 31131.0 | 0.9991 |  | 0.5-250 | 0.30 |
| Quizalofop-p-ethyl | y = 330299x - 225897 | 0.9981 | 1.00 | 0.5-250 | 4.19 |
|  | y = 331894x + 248432 | 0.9993 |  | 0.5-250 | 1.54 |
| Simeconazole | y = 138590x + 91333.8 | 0.9996 | 0.71 | 0.5-250 | 1.21 |
|  | y = 196491x - 29091.1 | 0.9995 |  | 0.5-250 | 1.96 |
| Spirodiclofen | y = 40610.7x + 61744.9 | 0.9993 | 0.88 | 0.5-250 | 0.54 |
|  | y = 46401.8x + 13914.9 | 0.9998 |  | 0.5-250 | 0.49 |
| Sulfotep | y = 194173x + 297555 | 0.9995 | 0.86 | 0.5-250 | 4.97 |
|  | y = 225425x + 566533 | 0.9994 |  | 0.5-250 | 1.27 |
| Sulprofos | y = 145213x - 599765 | 0.9935 | 1.05 | 0.5-250 | 4.99 |
|  | y = 138275x - 150856 | 0.9992 |  | 0.5-250 | 1.84 |
| Tebuconazole | y = 152811x + 163036 | 0.9994 | 0.95 | 0.5-250 | 3.55 |
|  | y = 160048x + 13926.9 | 0.9998 |  | 0.5-250 | 1.26 |
| Tebufenozide | y = 57419x + 85228.5 | 0.9995 | 0.97 | 0.5-250 | 3.57 |
|  | y = 59088.7x + 32806.8 | 0.9999 |  | 0.5-250 | 1.02 |
| Tebufenpyrad | y = 94263.5x + 153763 | 0.9997 | 0.89 | 0.5-250 | 1.88 |
|  | y = 106275x - 191860 | 0.9991 |  | 0.5-250 | 1.57 |
| Temephos | y = 342179x + 1415680 | 0.9983 | 0.98 | 0.5-250 | 2.11 |
|  | y = 347483x + 1162690 | 0.9994 |  | 0.5-250 | 0.71 |
| Terbufos | y = 8745.49x + 37049.9 | 0.9985 | 1.03 | 0.5-250 | 1.99 |
|  | y = 8518.68x + 33819.1 | 0.9992 |  | 0.5-250 | 1.92 |
| Tetrachlorvinphos | y = 38035.7x + 141135 | 0.9986 | 1.01 | 0.5-250 | 3.97 |
|  | y = 37520.1x + 71579.9 | 0.9998 |  | 0.5-250 | 0.68 |
| Tetraconazole | y = 144728x + 145793 | 0.9980 | 0.84 | 0.5-100 | 2.61 |
|  | y = 172206x + 18684.8 | 0.9996 |  | 0.5-100 | 1.01 |
| Thiacloprid | y = 211087x - 82604.4 | 0.9993 | 0.51 | 0.5-250 | 1.21 |
|  | y = 416872x + 1377520 | 0.9993 |  | 0.5-250 | 2.00 |
| Thiamethoxam | y = 29584.3x - 30239.7 | 0.9987 | 0.21 | 0.5-250 | 1.76 |
|  | y = 139911x + 4966.57 | 0.9999 |  | 0.5-250 | 1.81 |
| Thiophanate-methyl | y = 66686.2x - 329983 | 0.9953 | 0.20 | 1-250 | 2.84 |
|  | y = 338599x + 515079 | 0.9995 |  | 0.5-250 | 1.40 |
| Tolfenpyrad | y = 100986x + 280167 | 0.9986 | 1.05 | 0.5-250 | 3.80 |
|  | y = 96485.1x + 162015 | 0.9994 |  | 0.5-250 | 0.99 |
| Triadimefon | y = 44932.6x + 152764 | 0.9988 | 0.78 | 0.5-250 | 4.57 |
|  | y = 57301.5x + 90066.9 | 0.9994 |  | 0.5-250 | 1.33 |
| Triadimenol | y = 27573.7x - 2393.97 | 0.9996 | 0.99 | 0.5-250 | 2.23 |
|  | y = 27795.9x + 4696.08 | 0.9999 |  | 0.5-250 | 1.77 |
| Tricyclazole | y = 72550.8x + 5470.68 | 0.9995 | 0.31 | 0.5-250 | 1.56 |
|  | y = 234231x + 317911 | 0.9995 |  | 0.5-250 | 1.84 |
| Trifloxystrobin | y = 475552x + 1239650 | 0.9971 | 1.32 | 0.5-200 | 2.67 |
|  | y = 361172x + 1671560 | 0.9984 |  | 0.5-250 | 0.38 |
| Triticonazole | y = 67122.6x + 38206.4 | 0.9999 | 0.67 | 0.5-250 | 4.37 |
|  | y = 100738x - 76418.6 | 0.9994 |  | 0.5-250 | 1.78 |
| Uniconazole | y = 93274.9x + 94283.5 | 0.9996 | 0.88 | 0.5-250 | 1.15 |
|  | y = 106013x + 353989 | 0.9992 |  | 0.5-250 | 1.52 |
| Vitavax | y = 1011560x + 253641 | 0.9995 | 0.31 | 0.5-50 | 3.57 |
|  | y = 3312810x - 605463 | 0.9996 |  | 0.5-50 | 1.81 |

^1^ Calibration curves are expressed as regression lines (y = a x + b), where y is the peak area and x is the concentration (μg/L), a is the slope, b is the intercept and R is the correlation coefficient.

^2^ The value of slope ratio is calculated by the slope of the matrix-matched calibration curve to the slope of the standard calibration curve in solvent.

^3^ The LOD was the three times of the singal to noise ratio.

^4^ The first calibration curve for each pesticide represents the matrix-matched calibration curve.

^5^ The second calibration curve for each pesticide represents the standard calibration curve in solvent.

Table S6 The method validation of the linearity, matrix effect and LODs of each pesticide in PRA-HN.

| Pesticides | ^1^y=a x+b | R | ^2^Sloperatio | Linear range, | ^3^LOD, |
| --- | --- | --- | --- | --- | --- |
|  |  |  |  | μg/L | μg/kg |
| 3-Hydroxy-carbofuran | ^4^y = 3398.04x - 7188.07 | 0.9987 | 0.15 | 0.5-250 | 3.50 |
|  | ^5^y = 22478.1x - 8901.3 | 0.9997 |  | 0.5-250 | 0.28 |
| Abamectin | y = 6357.41x + 612.129 | 0.9963 | 1.82 | 1-250 | 2.96 |
|  | y = 3495.24x + 1957.13 | 0.9999 |  | 1-250 | 0.25 |
| Acephate | y = 81772.1x - 10148.6 | 0.9996 | 0.84 | 0.5-250 | 2.92 |
|  | y = 96800.2x - 3819.54 | 0.9996 |  | 0.5-250 | 1.59 |
| Acetamiprid | y = 30955x + 8207.68 | 0.9995 | 0.14 | 0.5-250 | 2.85 |
|  | y = 223248x + 47946 | 0.9996 |  | 0.5-250 | 1.81 |
| Alachlor | y = 730473x + 2182760 | 0.9988 | 0.92 | 0.5-250 | 3.01 |
|  | y = 792318x + 4126980 | 0.9984 |  | 0.5-250 | 1.76 |
| Atrazine | y = 157976x + 7008.28 | 0.9992 | 0.78 | 0.5-250 | 3.90 |
|  | y = 202545x + 326109 | 0.9992 |  | 0.5-250 | 0.21 |
| Azaconazole | y = 196581x - 15813 | 0.9997 | 0.73 | 0.5-250 | 4.86 |
|  | y = 268372x + 133688 | 0.9995 |  | 0.5-250 | 1.94 |
| Azamethiphos | y = 47816.1x - 52649.1 | 0.9981 | 0.49 | 0.5-250 | 2.42 |
|  | y = 98024.9x - 20835.8 | 0.9999 |  | 0.5-250 | 1.76 |
| Azoxystrobin | y = 627560x - 903987 | 0.9972 | 0.84 | 0.5-100 | 0.23 |
|  | y = 743784x + 71347.5 | 0.9997 |  | 0.5-100 | 0.51 |
| Benalaxyl | y = 178484x - 293669 | 0.9975 | 1.06 | 0.5-250 | 4.09 |
|  | y = 167645x - 185188 | 0.9997 |  | 0.5-250 | 1.46 |
| Bendiocarb | y = 8886.26x - 17062 | 0.9992 | 0.09 | 0.5-250 | 0.81 |
|  | y = 96648.3x + 211203 | 0.9991 |  | 0.5-250 | 0.84 |
| Bitertanol | y = 6019.34x + 1529.12 | 0.9984 | 1.15 | 1-250 | 1.60 |
|  | y = 5224.81x + 10194.4 | 0.9993 |  | 0.5-250 | 1.59 |
| Boscalid | y = 86324x + 178615 | 0.9984 | 0.91 | 0.5-250 | 3.82 |
|  | y = 95363.2x + 84231.6 | 0.9994 |  | 0.5-250 | 0.91 |
| Bromuconazole | y = 30888.5x - 20613 | 0.9975 | 0.92 | 0.5-250 | 0.85 |
|  | y = 33592.1x + 11743.8 | 0.9997 |  | 0.5-250 | 0.74 |
| Buprofezin | y = 295675x + 542958 | 0.9988 | 0.98 | 0.5-250 | 4.53 |
|  | y = 301316x + 498285 | 0.9993 |  | 0.5-250 | 0.85 |
| Butachlor | y = 158705x + 1108950 | 0.9926 | 0.99 | 0.5-250 | 4.38 |
|  | y = 160435x + 619420 | 0.9981 |  | 0.5-250 | 0.79 |
| Cadusafos | y = 307297x + 299036 | 0.9996 | 0.97 | 0.5-250 | 3.04 |
|  | y = 315842x + 527213 | 0.9994 |  | 0.5-250 | 0.62 |
| Carbendazim | y = 114117x - 14378.7 | 0.9998 | 0.34 | 0.5-250 | 3.38 |
|  | y = 334448x + 280153 | 0.9993 |  | 0.5-250 | 1.21 |
| Carbofuran | y = 29997.2x + 10409.8 | 0.9997 | 0.10 | 0.5-250 | 1.04 |
|  | y = 288008x + 235300 | 0.9992 |  | 0.5-250 | 0.64 |
| Chlorantraniliprole | y = 52214.6x + 120308 | 0.9993 | 0.89 | 0.5-250 | 1.45 |
|  | y = 58737.3x + 167462 | 0.9993 |  | 0.5-250 | 0.57 |
| Chlorpyrifos | y = 706947x + 1156960 | 0.9981 | 0.92 | 0.5-100 | 2.91 |
|  | y = 771286x + 1044540 | 0.9988 |  | 0.5-100 | 1.25 |
| Chlortoluron | y = 125147x + 109145 | 0.9993 | 0.47 | 0.5-250 | 2.02 |
|  | y = 265439x + 701160 | 0.9989 |  | 0.5-250 | 1.68 |
| Clothianidin | y = 4142.01x - 10893.3 | 0.9996 | 0.07 | 1-250 | 2.42 |
|  | y = 63358x + 45132.3 | 0.9998 |  | 0.5-250 | 1.08 |
| Coumaphos | y = 235774x - 158286 | 0.9987 | 1.13 | 0.5-200 | 2.65 |
|  | y = 209361x + 639593 | 0.9992 |  | 0.5-250 | 0.86 |
| Cyanophenphos | y = 36501.5x + 145973 | 0.9977 | 0.99 | 0.5-250 | 3.34 |
|  | y = 36866.1x + 100916 | 0.9992 |  | 0.5-250 | 0.84 |
| Cyazofamid` | y = 59035.5x - 67063 | 0.9968 | 0.74 | 0.5-250 | 1.30 |
|  | y = 79298.7x + 188035 | 0.9991 |  | 0.5-250 | 0.52 |
| Cyproconazole | y = 91372.3x - 36380.4 | 0.9989 | 0.93 | 0.5-250 | 0.87 |
|  | y = 98745.4x + 278702 | 0.9993 |  | 0.5-250 | 1.23 |
| Cyprodinil | y = 125763x + 4304.53 | 0.9994 | 0.93 | 0.5-250 | 0.26 |
|  | y = 135850x + 86987.3 | 0.9995 |  | 0.5-250 | 1.37 |
| Dicofol | y = 8553.76x + 1280.62 | 0.9992 | 1.01 | 0.5-100 | 3.54 |
|  | y = 8500.96x - 2216.31 | 0.9980 |  | 0.5-100 | 1.61 |
| Dicrotophos | y = 5715.76x - 9595.42 | 0.9997 | 0.11 | 0.5-250 | 4.90 |
|  | y = 53568.7x - 4127.35 | 0.9998 |  | 0.5-250 | 0.43 |
| Difenoconazole | y = 151030x - 94608.9 | 0.9995 | 1.10 | 0.5-250 | 4.52 |
|  | y = 137817x - 48437.3 | 0.9998 |  | 0.5-250 | 2.00 |
| Dimethoate | y = 50409.5x - 572.922 | 0.9996 | 0.24 | 0.5-250 | 2.50 |
|  | y = 213398x - 125164 | 0.9999 |  | 0.5-250 | 1.49 |
| Dimethomorph | y = 110896x + 193642 | 0.9996 | 0.95 | 0.5-250 | 0.20 |
|  | y = 116953x + 178724 | 0.9993 |  | 0.5-250 | 1.05 |
| dimethyl phthalate | y = 81663x + 1545160 | 0.9985 | 0.70 | 0.5-250 | 4.98 |
|  | y = 117004x + 1609990 | 0.9995 |  | 0.5-250 | 0.42 |
| Diniconazole | y = 45883.3x + 60175.7 | 0.9993 | 1.01 | 0.5-250 | 3.17 |
|  | y = 45491.1x + 94764.9 | 0.9990 |  | 0.5-250 | 0.89 |
| Dinotefuran | y = 38687.6x + 63464.5 | 0.9993 | 0.65 | 0.5-250 | 2.91 |
|  | y = 59625.9x + 44005.2 | 0.9997 |  | 0.5-250 | 1.02 |
| Emamectin B1 Benzoate | y = 331471x + 71852.9 | 0.9997 | 1.00 | 0.5-250 | 2.80 |
|  | y = 331424x - 70644.4 | 0.9992 |  | 0.5-250 | 0.38 |
| Epoxiconazole | y = 82965.2x - 863.679 | 0.9991 | 0.66 | 0.5-250 | 3.23 |
|  | y = 124881x - 43441 | 0.9992 |  | 0.5-250 | 0.24 |
| Ethiofencarb | y = 420904x - 143783 | 0.9998 | 0.30 | 0.5-50 | 4.79 |
|  | y = 1422230x + 597334 | 0.9961 |  | 0.5-50 | 1.36 |
| Etofenprox | y = 254787x - 46141.5 | 0.9996 | 1.11 | 0.5-250 | 1.18 |
|  | y = 229507x + 101162 | 0.9995 |  | 0.5-250 | 0.31 |
| Etoxazole | y = 734959x + 419583 | 0.9980 | 1.14 | 0.5-100 | 1.20 |
|  | y = 643096x + 534898 | 0.9997 |  | 0.5-100 | 0.58 |
| Fenamiphos | y = 406783x + 1081690 | 0.9967 | 1.06 | 0.5-200 | 3.14 |
|  | y = 381984x + 1564290 | 0.9990 |  | 0.5-250 | 1.51 |
| Fenamiphos sulfoxide | y = 44931.5x - 1362.95 | 0.9995 | 0.32 | 0.5-250 | 2.57 |
|  | y = 142107x + 36275.4 | 0.9999 |  | 0.5-250 | 1.56 |
| Fenbuconazole | y = 71552.2x - 236107 | 0.9960 | 0.93 | 0.5-250 | 2.08 |
|  | y = 77275.9x - 195151 | 0.9990 |  | 0.5-250 | 1.25 |
| Fenpropathrin | y = 10187.8x + 14547.3 | 0.9988 | 1.04 | 0.5-100 | 4.14 |
|  | y = 9836.11x + 22907.2 | 0.9966 |  | 0.5-100 | 1.24 |
| Fipronil | y = 8066.50x + 16184.8 | 0.9976 | 0.75 | 0.5-250 | 4.23 |
|  | y = 10769.2x - 17592.3 | 0.9991 |  | 0.5-250 | 0.62 |
| Fipronil-sulfone | y = 138054x - 79614.1 | 0.9973 | 0.75 | 0.5-250 | 2.10 |
|  | y = 183973x - 50212.4 | 0.9994 |  | 0.5-250 | 1.64 |
| Fludioxonil | y = 28703.1x + 141448 | 0.9966 | 0.93 | 0.5-200 | 0.22 |
|  | y = 30914.8x + 86835 | 0.9992 |  | 0.5-250 | 0.19 |
| Fluopyram | y = 189845x - 90104.9 | 0.9981 | 0.88 | 0.5-250 | 3.88 |
|  | y = 216694x - 397443 | 0.9993 |  | 0.5-250 | 0.44 |
| Fluoxastrobin | y = 312293x + 1207510 | 0.9977 | 0.96 | 0.5-250 | 4.09 |
|  | y = 326343x + 835206 | 0.9994 |  | 0.5-250 | 2.00 |
| Flusilazole | y = 152300x - 246112 | 0.9973 | 0.97 | 0.5-250 | 1.92 |
|  | y = 157601x - 219056 | 0.9993 |  | 0.5-250 | 1.09 |
| Flutolanil | y = 461042x - 87800 | 0.9996 | 0.76 | 0.5-100 | 1.17 |
|  | y = 604339x - 162788 | 0.9997 |  | 0.5-100 | 1.70 |
| Flutriafol | y = 24400.2x - 5105.53 | 0.9989 | 0.54 | 0.5-250 | 1.96 |
|  | y = 45577x - 12136.6 | 0.9995 |  | 0.5-250 | 1.93 |
| Forchlorfenuron | y = 74252.4x + 119528 | 0.9990 | 0.85 | 0.5-250 | 2.05 |
|  | y = 87807.5x + 280399 | 0.9992 |  | 0.5-250 | 1.86 |
| Hexaconazole | y = 69597.7x + 30220.5 | 0.9992 | 1.04 | 0.5-250 | 4.27 |
|  | y = 66860.6x + 53485.5 | 0.9995 |  | 0.5-250 | 1.26 |
| Hexaflumuron | y = 350664x + 2040050 | 0.9955 | 1.00 | 0.5-250 | 3.75 |
|  | y = 349339x + 1178920 | 0.9992 |  | 0.5-250 | 1.33 |
| Hexazinone | y = 253650x - 40038.8 | 0.9997 | 0.35 | 0.5-100 | 1.59 |
|  | y = 717261x + 309539 | 0.9998 |  | 0.5-100 | 0.97 |
| Imazalil | y = 91751.2x + 125181 | 0.9993 | 0.90 | 0.5-250 | 3.41 |
|  | y = 101466x + 89024.5 | 0.9996 |  | 0.5-250 | 1.26 |
| Indoxacarb | y = 18644.9x + 70732.1 | 0.9986 | 0.96 | 0.5-250 | 2.57 |
|  | y = 19396x + 47574.7 | 0.9991 |  | 0.5-250 | 1.90 |
| Ipconazole | y = 141979x - 20409.4 | 0.9997 | 0.82 | 0.5-250 | 3.19 |
|  | y = 173211x + 311393 | 0.9992 |  | 0.5-250 | 0.90 |
| Isoprocarb | y = 118325x - 6070.57 | 0.9968 | 0.55 | 0.5-250 | 0.76 |
|  | y = 213432x - 324303 | 0.9991 |  | 0.5-250 | 0.62 |
| Kresoxim-methyl | y = 53005.6x - 25546.6 | 0.9998 | 0.87 | 0.5-100 | 2.78 |
|  | y = 60608.7x - 32216 | 0.9997 |  | 0.5-100 | 0.90 |
| Lufenuron | y = 12654.5x + 94238.4 | 0.9909 | 0.95 | 0.5-200 | 2.30 |
|  | y = 13251.2x + 78340.9 | 0.9980 |  | 0.5-250 | 0.76 |
| Malaoxon | y = 164927x - 198016 | 0.9991 | 0.20 | 0.5-100 | 0.65 |
|  | y = 839041x + 501694 | 0.9998 |  | 0.5-100 | 0.79 |
| Malathion | y = 119732x + 248272 | 0.9994 | 0.91 | 0.5-250 | 1.94 |
|  | y = 132104x + 396108 | 0.9994 |  | 0.5-250 | 1.36 |
| Mefenoxam | y = 541908x - 102444 | 0.9996 | 0.84 | 0.5-100 | 0.23 |
|  | y = 646361x + 696020 | 0.9995 |  | 0.5-100 | 1.24 |
| Mepronil | y = 2517060x + 365169 | 0.9991 | 0.79 | 0.5-50 | 4.14 |
|  | y = 3175610x + 459022 | 0.9997 |  | 0.5-50 | 0.67 |
| Metalaxyl | y = 576357x - 314034 | 0.9997 | 0.80 | 0.5-100 | 4.48 |
|  | y = 718117x + 142354 | 0.9999 |  | 0.5-100 | 1.77 |
| Methidathion | y = 31007.8x - 13007.7 | 0.9983 | 0.80 | 0.5-250 | 3.01 |
|  | y = 38779.7x + 8462.38 | 0.9997 |  | 0.5-250 | 0.61 |
| Methomyl | y = 37835.5x + 11192.8 | 0.9998 | 0.53 | 0.5-250 | 1.74 |
|  | y = 70897.1x - 100837 | 0.9996 |  | 0.5-250 | 0.83 |
| Methoprene | y = 23349.7x + 32052 | 0.9995 | 0.98 | 0.5-250 | 2.20 |
|  | y = 23855.3x + 1560.05 | 0.9994 |  | 0.5-250 | 1.17 |
| Methoxyfenozide | y = 71774.3x + 152327 | 0.9987 | 0.97 | 0.5-250 | 3.11 |
|  | y = 74074.9x + 70085.8 | 0.9992 |  | 0.5-250 | 1.52 |
| Mevinphos | y = 18969.1x + 13046.1 | 0.9995 | 0.29 | 0.5-250 | 3.51 |
|  | y = 66496x - 64412.2 | 0.9995 |  | 0.5-250 | 1.88 |
| Monocrotophos | y = 4999.63x - 8691.73 | 0.9991 | 0.14 | 1-250 | 0.99 |
|  | y = 34513.8x + 11875.1 | 0.9997 |  | 0.5-250 | 0.19 |
| Myclobutanil | y = 62776.3x + 183020 | 0.9993 | 0.94 | 0.5-250 | 4.62 |
|  | y = 67118.4x + 142114 | 0.9997 |  | 0.5-250 | 1.92 |
| Omethoate | y = 71461.3x - 6734.56 | 0.9999 | 0.79 | 0.5-250 | 0.16 |
|  | y = 89900.3x - 116146 | 0.9998 |  | 0.5-250 | 1.37 |
| Paclobutrazol | y = 148119x + 310698 | 0.9991 | 0.85 | 0.5-250 | 4.07 |
|  | y = 174702x + 90474.6 | 0.9990 |  | 0.5-250 | 0.68 |
| Penconazole | y = 96258.5x + 29303.6 | 0.9986 | 1.05 | 0.5-250 | 4.40 |
|  | y = 91994.6x + 140739 | 0.9991 |  | 0.5-250 | 0.51 |
| Phenthoate | y = 125835x + 620293 | 0.9972 | 0.93 | 0.5-250 | 0.11 |
|  | y = 135636x + 493223 | 0.9994 |  | 0.5-250 | 0.52 |
| Phorate | y = 33865x + 46259.6 | 0.9986 | 0.93 | 0.5-250 | 1.32 |
|  | y = 36585.5x + 41822.3 | 0.9990 |  | 0.5-250 | 0.78 |
| Phorate sulfone | y = 26412.1x - 1650.8 | 0.9999 | 0.48 | 0.5-100 | 4.72 |
|  | y = 55229x + 44723.2 | 0.9995 |  | 0.5-100 | 1.97 |
| Phosalone | y = 1251580x + 976036 | 0.9976 | 0.98 | 0.5-50 | 1.27 |
|  | y = 1281220x + 826217 | 0.9988 |  | 0.5-50 | 0.16 |
| Phosfolan | y = 165600x + 112378 | 0.9995 | 0.47 | 0.5-250 | 0.99 |
|  | y = 351640x + 793462 | 0.9993 |  | 0.5-250 | 1.99 |
| Phosmet | y = 152592x - 132296 | 0.9991 | 0.82 | 0.5-200 | 4.34 |
|  | y = 187056x + 250378 | 0.9999 |  | 0.5-250 | 1.43 |
| Phosphamidon | y = 96307.4x - 27271.6 | 0.9994 | 0.75 | 0.5-250 | 0.27 |
|  | y = 128857x - 145106 | 0.9998 |  | 0.5-250 | 1.88 |
| Piperonyl butoxide | y = 332070x + 1111450 | 0.9967 | 1.01 | 0.5-250 | 1.43 |
|  | y = 328481x + 511020 | 0.9992 |  | 0.5-250 | 1.87 |
| Pirimicarb | y = 219019x + 166357 | 0.9994 | 0.65 | 0.5-250 | 1.40 |
|  | y = 335094x + 1196200 | 0.9990 |  | 0.5-250 | 0.18 |
| Pirimiphos-methyl | y = 308502x + 364702 | 0.9991 | 0.96 | 0.5-250 | 1.24 |
|  | y = 322697x + 282327 | 0.9994 |  | 0.5-250 | 0.22 |
| Prochloraz | y = 179099x + 33991 | 0.9981 | 0.97 | 0.5-250 | 0.79 |
|  | y = 184363x - 38432 | 0.9993 |  | 0.5-250 | 0.81 |
| Profenofos | y = 92198.5x + 265634 | 0.9993 | 0.82 | 0.5-250 | 2.33 |
|  | y = 112112x + 241932 | 0.9991 |  | 0.5-250 | 1.33 |
| Propamocarb | y = 192456x + 14432.1 | 0.9995 | 0.73 | 0.5-250 | 0.90 |
|  | y = 264294x - 325221 | 0.9996 |  | 0.5-250 | 1.38 |
| Propiconazole | y = 99457.5x + 53138.1 | 0.9998 | 0.97 | 0.5-250 | 0.22 |
|  | y = 102586x + 216541 | 0.9994 |  | 0.5-250 | 0.66 |
| Prothiophos | y = 46808.8x - 90779.8 | 0.9992 | 0.96 | 1-250 | 1.05 |
|  | y = 48650.7x - 3180.76 | 0.9980 |  | 1-250 | 1.97 |
| Pyraclostrobine | y = 190937x + 186591 | 0.9982 | 1.02 | 0.5-250 | 3.24 |
|  | y = 186375x + 307622 | 0.9992 |  | 0.5-250 | 1.53 |
| Pyridaben | y = 256636x + 90860.9 | 0.9998 | 0.99 | 0.5-250 | 2.21 |
|  | y = 260273x + 50248.3 | 0.9998 |  | 0.5-250 | 0.53 |
| Pyridafenthion | y = 214223x + 526743 | 0.9979 | 1.05 | 0.5-250 | 4.81 |
|  | y = 204024x + 191747 | 0.9994 |  | 0.5-250 | 0.63 |
| Pyrimethanil | y = 59699.6x + 118722 | 0.9988 | 0.91 | 0.5-250 | 4.46 |
|  | y = 65358.3x + 120705 | 0.9994 |  | 0.5-250 | 1.69 |
| Pyrimitate | y = 808967x + 1547230 | 0.9962 | 0.97 | 0.5-100 | 4.21 |
|  | y = 835352x + 732844 | 0.9995 |  | 0.5-100 | 1.12 |
| Pyriproxyfen | y = 391831x + 691527 | 0.9978 | 1.15 | 0.5-200 | 2.33 |
|  | y = 339296x + 923521 | 0.9990 |  | 0.5-250 | 1.56 |
| Pyrisoxazole | y = 11766x + 37605.8 | 0.9974 | 1.13 | 0.5-250 | 3.48 |
|  | y = 10389.2x + 31131 | 0.9991 |  | 0.5-250 | 0.30 |
| Quizalofop-p-ethyl | y = 306311x + 93398.4 | 0.9999 | 0.92 | 0.5-250 | 0.49 |
|  | y = 331894x + 248432 | 0.9993 |  | 0.5-250 | 1.54 |
| Simeconazole | y = 155393x - 228166 | 0.9972 | 0.79 | 0.5-250 | 1.33 |
|  | y = 196491x - 29091.1 | 0.9995 |  | 0.5-250 | 1.96 |
| Spirodiclofen | y = 41067.7x + 49755.8 | 0.9993 | 0.89 | 0.5-250 | 1.08 |
|  | y = 46401.8x + 13914.9 | 0.9998 |  | 0.5-250 | 0.49 |
| Sulfotep | y = 191302x + 659171 | 0.9964 | 0.85 | 0.5-250 | 3.28 |
|  | y = 225425x + 566533 | 0.9994 |  | 0.5-250 | 1.27 |
| Sulprofos | y = 132744x - 203941 | 0.9981 | 0.96 | 0.5-250 | 4.55 |
|  | y = 138275x - 150856 | 0.9992 |  | 0.5-250 | 1.84 |
| Tebuconazole | y = 151461x + 110879 | 0.9992 | 0.95 | 0.5-250 | 2.34 |
|  | y = 160048x + 13926.9 | 0.9998 |  | 0.5-250 | 1.26 |
| Tebufenozide | y = 52712.3x + 78308 | 0.9991 | 0.89 | 0.5-250 | 0.45 |
|  | y = 59088.7x + 32806.8 | 0.9999 |  | 0.5-250 | 1.02 |
| Tebufenpyrad | y = 101418x - 109411 | 0.9991 | 0.95 | 0.5-250 | 0.85 |
|  | y = 106275x - 191860 | 0.9991 |  | 0.5-250 | 1.57 |
| Temephos | y = 343261x + 1296560 | 0.9979 | 0.99 | 0.5-250 | 3.46 |
|  | y = 347483x + 1162690 | 0.9994 |  | 0.5-250 | 0.71 |
| Terbufos | y = 8837.27x + 40871.5 | 0.9974 | 1.04 | 0.5-250 | 2.71 |
|  | y = 8518.68x + 33819.1 | 0.9992 |  | 0.5-250 | 1.92 |
| Tetrachlorvinphos | y = 35056.7x + 111161 | 0.9981 | 0.93 | 0.5-250 | 0.89 |
|  | y = 37520.1x + 71579.9 | 0.9998 |  | 0.5-250 | 0.68 |
| Tetraconazole | y = 140223x + 188014 | 0.9987 | 0.81 | 0.5-100 | 2.43 |
|  | y = 172206x + 18684.8 | 0.9996 |  | 0.5-100 | 1.01 |
| Thiacloprid | y = 236003x - 194811 | 0.9995 | 0.57 | 0.5-250 | 3.40 |
|  | y = 416872x + 1377520 | 0.9993 |  | 0.5-250 | 2.00 |
| Thiamethoxam | y = 32935.8x + 7014.99 | 0.9997 | 0.24 | 0.5-250 | 3.01 |
|  | y = 139911x + 4966.57 | 0.9999 |  | 0.5-250 | 1.81 |
| Thiophanate-methyl | y = 75169.5x - 369619 | 0.9931 | 0.22 | 0.5-200 | 0.36 |
|  | y = 338599x + 515079 | 0.9995 |  | 0.5-250 | 1.40 |
| Tolfenpyrad | y = 96756x + 250670 | 0.9993 | 1.00 | 0.5-250 | 1.07 |
|  | y = 96485.1x + 162015 | 0.9994 |  | 0.5-250 | 0.99 |
| Triadimefon | y = 43708.4x + 135567 | 0.9979 | 0.76 | 0.5-250 | 2.51 |
|  | y = 57301.5x + 90066.9 | 0.9994 |  | 0.5-250 | 1.33 |
| Triadimenol | y = 29103.2x - 27512.3 | 0.9997 | 1.05 | 0.5-250 | 1.07 |
|  | y = 27795.9x + 4696.08 | 0.9999 |  | 0.5-250 | 1.77 |
| Tricyclazole | y = 79135.5x + 25849.3 | 0.9997 | 0.34 | 0.5-250 | 4.68 |
|  | y = 234231x + 317911 | 0.9995 |  | 0.5-250 | 1.84 |
| Trifloxystrobin | y = 432010x + 1568620 | 0.9967 | 1.20 | 0.5-200 | 2.20 |
|  | y = 361172x + 1671560 | 0.9984 |  | 0.5-250 | 0.38 |
| Triticonazole | y = 66837.1x + 12642.7 | 0.9997 | 0.66 | 0.5-250 | 2.35 |
|  | y = 100738x - 76418.6 | 0.9994 |  | 0.5-250 | 1.78 |
| Uniconazole | y = 99043.7x + 58050.7 | 0.9988 | 0.93 | 0.5-250 | 4.35 |
|  | y = 106013x + 353989 | 0.9992 |  | 0.5-250 | 1.52 |
| Vitavax | y = 1133710x - 208539 | 0.9989 | 0.34 | 0.5-50 | 0.92 |
|  | y = 3312810x - 605463 | 0.9996 |  | 0.5-50 | 1.81 |

^1^ Calibration curves are expressed as regression lines (y = a x + b), where y is the peak area and x is the concentration (μg/L), a is the slope, b is the intercept and R is the correlation coefficient.

^2^ The value of slope ratio is calculated by the slope of the matrix-matched calibration curve to the slope of the standard calibration curve in solvent.

^3^ The LOD was the three times of the singal to noise ratio.

^4^ The first calibration curve for each pesticide represents the matrix-matched calibration curve.

^5^ The second calibration curve for each pesticide represents the standard calibration curve in solvent.

Table S7 The method validation of the linearity, matrix effect and LODs of each pesticide in PRA-HB.

| Pesticides | ^1^y=a x+b | R | ^2^Sloperatio | Linear range, | ^3^LOD, |
| --- | --- | --- | --- | --- | --- |
|  |  |  |  | μg/L | μg/kg |
| 3-Hydroxy-carbofuran | ^4^y = 3302.52x - 3082.26 | 0.9989 | 0.15 | 0.5-250 | 1.55 |
|  | ^5^y = 22478.1x - 8901.3 | 0.9997 |  | 0.5-250 | 0.28 |
| Abamectin | y = 6404.04x - 14175.5 | 0.9921 | 1.83 | 2-250 | 1.35 |
|  | y = 3495.24x + 1957.13 | 0.9999 |  | 1-250 | 0.25 |
| Acephate | y = 84426.1x + 28437.7 | 0.9995 | 0.87 | 0.5-250 | 0.16 |
|  | y = 96800.2x - 3819.54 | 0.9996 |  | 0.5-250 | 1.59 |
| Acetamiprid | y = 30012.2x + 51588.4 | 0.9996 | 0.13 | 0.5-250 | 0.94 |
|  | y = 223248x + 47946 | 0.9996 |  | 0.5-250 | 1.81 |
| Alachlor | y = 723805x + 4426990 | 0.9958 | 0.91 | 0.5-250 | 1.03 |
|  | y = 792318x + 4126980 | 0.9984 |  | 0.5-250 | 1.76 |
| Atrazine | y = 162386x - 78599.7 | 0.9998 | 0.80 | 0.5-250 | 0.17 |
|  | y = 202545x + 326109 | 0.9992 |  | 0.5-250 | 0.21 |
| Azaconazole | y = 205922x - 115757 | 0.9978 | 0.77 | 0.5-250 | 1.27 |
|  | y = 268372x + 133688 | 0.9995 |  | 0.5-250 | 1.94 |
| Azamethiphos | y = 44904.4x - 46035.7 | 0.9993 | 0.46 | 0.5-250 | 0.56 |
|  | y = 98024.9x - 20835.8 | 0.9999 |  | 0.5-250 | 1.76 |
| Azoxystrobin | y = 557610x + 7497.24 | 0.9999 | 0.75 | 0.5-100 | 3.93 |
|  | y = 743784x + 71347.5 | 0.9997 |  | 0.5-100 | 0.51 |
| Benalaxyl | y = 169506x + 39831.1 | 0.9994 | 1.01 | 0.5-250 | 1.93 |
|  | y = 167645x - 185188 | 0.9997 |  | 0.5-250 | 1.46 |
| Bendiocarb | y = 9705.74x + 1977.36 | 0.9994 | 0.10 | 0.5-250 | 3.95 |
|  | y = 96648.3x + 211203 | 0.9991 |  | 0.5-250 | 0.84 |
| Bitertanol | y = 6046.23x + 14038.4 | 0.9989 | 1.16 | 1-250 | 0.88 |
|  | y = 5224.81x + 10194.4 | 0.9993 |  | 0.5-250 | 1.59 |
| Boscalid | y = 101574x - 5702.06 | 0.9997 | 1.07 | 0.5-200 | 1.59 |
|  | y = 95363.2x + 84231.6 | 0.9994 |  | 0.5-250 | 0.91 |
| Bromuconazole | y = 31400.1x + 21463.7 | 0.9996 | 0.93 | 0.5-250 | 2.16 |
|  | y = 33592.1x + 11743.8 | 0.9997 |  | 0.5-250 | 0.74 |
| Buprofezin | y = 290283x + 315494 | 0.9997 | 0.96 | 0.5-250 | 2.22 |
|  | y = 301316x + 498285 | 0.9993 |  | 0.5-250 | 0.85 |
| Butachlor | y = 164648x + 831062 | 0.9972 | 1.03 | 0.5-250 | 0.82 |
|  | y = 160435x + 619420 | 0.9981 |  | 0.5-250 | 0.79 |
| Cadusafos | y = 319422x + 380141 | 0.9991 | 1.01 | 0.5-250 | 4.31 |
|  | y = 315842x + 527213 | 0.9994 |  | 0.5-250 | 0.62 |
| Carbendazim | y = 103378x + 69803.7 | 0.9996 | 0.31 | 0.5-250 | 1.50 |
|  | y = 334448x + 280153 | 0.9993 |  | 0.5-250 | 1.21 |
| Carbofuran | y = 35014.5x - 18424 | 0.9991 | 0.12 | 0.5-250 | 3.47 |
|  | y = 288008x + 235300 | 0.9992 |  | 0.5-250 | 0.64 |
| Chlorantraniliprole | y = 50253.7x + 163945 | 0.9984 | 0.86 | 0.5-250 | 2.11 |
|  | y = 58737.3x + 167462 | 0.9993 |  | 0.5-250 | 0.57 |
| Chlorpyrifos | y = 732469x + 1162650 | 0.9970 | 0.95 | 0.5-100 | 2.32 |
|  | y = 771286x + 1044540 | 0.9988 |  | 0.5-100 | 1.25 |
| Chlortoluron | y = 120215x + 91037.8 | 0.9998 | 0.45 | 0.5-250 | 0.20 |
|  | y = 265439x + 701160 | 0.9989 |  | 0.5-250 | 1.68 |
| Clothianidin | y = 3799x + 5644.90 | 0.9996 | 0.06 | 2-250 | 2.41 |
|  | y = 63358x + 45132.3 | 0.9998 |  | 0.5-250 | 1.08 |
| Coumaphos | y = 205071x + 383167 | 0.9995 | 0.98 | 0.5-250 | 0.66 |
|  | y = 209361x + 639593 | 0.9992 |  | 0.5-250 | 0.86 |
| Cyanophenphos | y = 36183.6x + 119645 | 0.9987 | 0.98 | 0.5-250 | 4.77 |
|  | y = 36866.1x + 100916 | 0.9992 |  | 0.5-250 | 0.84 |
| Cyazofamid` | y = 57852.5x - 22089.1 | 0.9988 | 0.73 | 0.5-200 | 2.28 |
|  | y = 79298.7x + 188035 | 0.9991 |  | 0.5-250 | 0.52 |
| Cyproconazole | y = 84764.2x + 223322 | 0.9970 | 0.86 | 0.5-250 | 3.04 |
|  | y = 98745.4x + 278702 | 0.9993 |  | 0.5-250 | 1.23 |
| Cyprodinil | y = 128117x - 226037 | 0.9985 | 0.94 | 0.5-250 | 3.43 |
|  | y = 135850x + 86987.3 | 0.9995 |  | 0.5-250 | 1.37 |
| Dicofol | y = 8393.97x + 11174 | 0.9968 | 0.99 | 0.5-100 | 3.41 |
|  | y = 8500.96x - 2216.31 | 0.9980 |  | 0.5-100 | 1.61 |
| Dicrotophos | y = 4914.45x - 3773.08 | 0.9998 | 0.09 | 0.5-250 | 4.05 |
|  | y = 53568.7x - 4127.35 | 0.9998 |  | 0.5-250 | 0.43 |
| Difenoconazole | y = 156726x - 61458.9 | 0.9998 | 1.14 | 0.5-250 | 0.35 |
|  | y = 137817x - 48437.3 | 0.9998 |  | 0.5-250 | 2.00 |
| Dimethoate | y = 50854.5x - 21182.9 | 0.9998 | 0.24 | 0.5-250 | 0.98 |
|  | y = 213398x - 125164 | 0.9999 |  | 0.5-250 | 1.49 |
| Dimethomorph | y = 197962x + 276768 | 0.9995 | 1.69 | 0.5-250 | 2.38 |
|  | y = 116953x + 178724 | 0.9993 |  | 0.5-250 | 1.05 |
| dimethyl phthalate | y = 81744.7x + 1390850 | 0.9987 | 0.70 | 0.5-250 | 2.72 |
|  | y = 117004x + 1609990 | 0.9995 |  | 0.5-250 | 0.42 |
| Diniconazole | y = 46363.2x + 1368.96 | 0.9993 | 1.02 | 0.5-250 | 1.43 |
|  | y = 45491.1x + 94764.9 | 0.9990 |  | 0.5-250 | 0.89 |
| Dinotefuran | y = 40020.9x + 24098.5 | 0.9999 | 0.67 | 0.5-250 | 1.75 |
|  | y = 59625.9x + 44005.2 | 0.9997 |  | 0.5-250 | 1.02 |
| Emamectin B1 Benzoate | y = 343901x - 263881 | 0.9995 | 1.04 | 0.5-250 | 3.16 |
|  | y = 331424x - 70644.4 | 0.9992 |  | 0.5-250 | 0.38 |
| Epoxiconazole | y = 83608.8x + 13089 | 0.9997 | 0.67 | 0.5-250 | 4.19 |
|  | y = 124881x - 43441 | 0.9992 |  | 0.5-250 | 0.24 |
| Ethiofencarb | y = 370730x + 16550.4 | 0.9996 | 0.26 | 0.5-50 | 3.57 |
|  | y = 1422230x + 597334 | 0.9961 |  | 0.5-50 | 1.36 |
| Etofenprox | y = 266935x - 268970 | 0.9996 | 1.16 | 0.5-250 | 1.92 |
|  | y = 229507x + 101162 | 0.9995 |  | 0.5-250 | 0.31 |
| Etoxazole | y = 742602x + 553548 | 0.9981 | 1.15 | 0.5-100 | 1.45 |
|  | y = 643096x + 534898 | 0.9997 |  | 0.5-100 | 0.58 |
| Fenamiphos | y = 360443x + 1812850 | 0.9966 | 0.94 | 0.5-250 | 3.90 |
|  | y = 381984x + 1564290 | 0.9990 |  | 0.5-250 | 1.51 |
| Fenamiphos sulfoxide | y = 42343.2x - 35453.4 | 0.9998 | 0.30 | 0.5-250 | 4.71 |
|  | y = 142107x + 36275.4 | 0.9999 |  | 0.5-250 | 1.56 |
| Fenbuconazole | y = 60642.2x + 74777.1 | 0.9962 | 0.78 | 0.5-250 | 3.80 |
|  | y = 77275.9x - 195151 | 0.9990 |  | 0.5-250 | 1.25 |
| Fenpropathrin | y = 11077.2x + 9074.67 | 0.9976 | 1.13 | 0.5-100 | 2.35 |
|  | y = 9836.11x + 22907.2 | 0.9966 |  | 0.5-100 | 1.24 |
| Fipronil | y = 8066.59x + 8889.87 | 0.9997 | 0.75 | 0.5-250 | 1.81 |
|  | y = 10769.2x - 17592.3 | 0.9991 |  | 0.5-250 | 0.62 |
| Fipronil-sulfone | y = 138882x - 56464.3 | 0.9993 | 0.75 | 0.5-250 | 4.06 |
|  | y = 183973x - 50212.4 | 0.9994 |  | 0.5-250 | 1.64 |
| Fludioxonil | y = 27074.1x + 158488 | 0.9974 | 0.88 | 0.5-250 | 0.60 |
|  | y = 30914.8x + 86835 | 0.9992 |  | 0.5-250 | 0.19 |
| Fluopyram | y = 196141x - 49557.1 | 0.9998 | 0.91 | 0.5-250 | 2.81 |
|  | y = 216694x - 397443 | 0.9993 |  | 0.5-250 | 0.44 |
| Fluoxastrobin | y = 326150x + 949835 | 0.9978 | 1.00 | 0.5-250 | 4.17 |
|  | y = 326343x + 835206 | 0.9994 |  | 0.5-250 | 2.00 |
| Flusilazole | y = 141573x + 84847.5 | 0.9972 | 0.90 | 0.5-250 | 4.96 |
|  | y = 157601x - 219056 | 0.9993 |  | 0.5-250 | 1.09 |
| Flutolanil | y = 403616x + 454141 | 0.9990 | 0.67 | 0.5-100 | 3.76 |
|  | y = 604339x - 162788 | 0.9997 |  | 0.5-100 | 1.70 |
| Flutriafol | y = 23428.9x + 9128.77 | 0.9996 | 0.51 | 1-250 | 4.64 |
|  | y = 45577x - 12136.6 | 0.9995 |  | 0.5-250 | 1.93 |
| Forchlorfenuron | y = 78054.1x + 18396.6 | 0.9998 | 0.89 | 0.5-250 | 2.76 |
|  | y = 87807.5x + 280399 | 0.9992 |  | 0.5-250 | 1.86 |
| Hexaconazole | y = 70878.2x - 35592.2 | 0.9991 | 1.06 | 0.5-250 | 1.71 |
|  | y = 66860.6x + 53485.5 | 0.9995 |  | 0.5-250 | 1.26 |
| Hexaflumuron | y = 362268x + 2102490 | 0.9938 | 1.04 | 0.5-250 | 2.16 |
|  | y = 349339x + 1178920 | 0.9992 |  | 0.5-250 | 1.33 |
| Hexazinone | y = 271536x - 79220.1 | 0.9999 | 0.38 | 0.5-100 | 4.98 |
|  | y = 717261x + 309539 | 0.9998 |  | 0.5-100 | 0.97 |
| Imazalil | y = 92646.8x + 24754.3 | 0.9989 | 0.91 | 0.5-250 | 1.84 |
|  | y = 101466x + 89024.5 | 0.9996 |  | 0.5-250 | 1.26 |
| Indoxacarb | y = 22053.5x + 9673.58 | 0.9976 | 1.14 | 0.5-200 | 2.86 |
|  | y = 19396x + 47574.7 | 0.9991 |  | 0.5-250 | 1.90 |
| Ipconazole | y = 130829x + 106976 | 0.9996 | 0.76 | 0.5-250 | 3.60 |
|  | y = 173211x + 311393 | 0.9992 |  | 0.5-250 | 0.90 |
| Isoprocarb | y = 112974x + 81884.8 | 0.9996 | 0.53 | 0.5-250 | 0.80 |
|  | y = 213432x - 324303 | 0.9991 |  | 0.5-250 | 0.62 |
| Kresoxim-methyl | y = 48766x + 3777.24 | 0.9998 | 0.80 | 0.5-100 | 2.14 |
|  | y = 60608.7x - 32216 | 0.9997 |  | 0.5-100 | 0.90 |
| Lufenuron | y = 13450.3x + 79497.3 | 0.9947 | 1.02 | 0.5-200 | 2.88 |
|  | y = 13251.2x + 78340.9 | 0.9980 |  | 0.5-250 | 0.76 |
| Malaoxon | y = 43209.6x - 10351.7 | 0.9989 | 0.05 | 0.5-100 | 1.00 |
|  | y = 839041x + 501694 | 0.9998 |  | 0.5-100 | 0.79 |
| Malathion | y = 115977x + 130014 | 0.9971 | 0.88 | 0.5-250 | 0.68 |
|  | y = 132104x + 396108 | 0.9994 |  | 0.5-250 | 1.36 |
| Mefenoxam | y = 535008x - 33425.7 | 0.9996 | 0.83 | 0.5-100 | 0.50 |
|  | y = 646361x + 696020 | 0.9995 |  | 0.5-100 | 1.24 |
| Mepronil | y = 1829080x + 4063210 | 0.9914 | 0.58 | 0.5-50 | 0.55 |
|  | y = 3175610x + 459022 | 0.9997 |  | 0.5-50 | 0.67 |
| Metalaxyl | y = 520642x - 43462.9 | 0.9996 | 0.73 | 0.5-100 | 3.23 |
|  | y = 718117x + 142354 | 0.9999 |  | 0.5-100 | 1.77 |
| Methidathion | y = 30902.3x + 9751.63 | 0.9984 | 0.80 | 0.5-250 | 0.18 |
|  | y = 38779.7x + 8462.38 | 0.9997 |  | 0.5-250 | 0.61 |
| Methomyl | y = 37618.9x - 18907.3 | 0.9992 | 0.53 | 0.5-250 | 1.18 |
|  | y = 70897.1x - 100837 | 0.9996 |  | 0.5-250 | 0.83 |
| Methoprene | y = 24285x + 13726.3 | 0.9997 | 1.02 | 0.5-250 | 3.67 |
|  | y = 23855.3x + 1560.05 | 0.9994 |  | 0.5-250 | 1.17 |
| Methoxyfenozide | y = 70220.1x + 268363 | 0.9977 | 0.95 | 0.5-250 | 2.90 |
|  | y = 74074.9x + 70085.8 | 0.9992 |  | 0.5-250 | 1.52 |
| Mevinphos | y = 19920.4x - 16010.4 | 0.9997 | 0.30 | 0.5-250 | 2.56 |
|  | y = 66496x - 64412.2 | 0.9995 |  | 0.5-250 | 1.88 |
| Monocrotophos | y = 4367.03x - 5767.3 | 0.9996 | 0.13 | 0.5-250 | 3.31 |
|  | y = 34513.8x + 11875.1 | 0.9997 |  | 0.5-250 | 0.19 |
| Myclobutanil | y = 69781.4x + 118497 | 0.9993 | 1.04 | 0.5-200 | 1.91 |
|  | y = 67118.4x + 142114 | 0.9997 |  | 0.5-250 | 1.92 |
| Omethoate | y = 69357.9x - 30617.4 | 0.9996 | 0.77 | 0.5-250 | 2.01 |
|  | y = 89900.3x - 116146 | 0.9998 |  | 0.5-250 | 1.37 |
| Paclobutrazol | y = 141377x + 431865 | 0.9989 | 0.81 | 0.5-250 | 2.68 |
|  | y = 174702x + 90474.6 | 0.9990 |  | 0.5-250 | 0.68 |
| Penconazole | y = 103751x + 96129.6 | 0.9998 | 1.13 | 0.5-200 | 4.73 |
|  | y = 91994.6x + 140739 | 0.9991 |  | 0.5-250 | 0.51 |
| Phenthoate | y = 150519x + 176095 | 0.9990 | 1.11 | 0.5-200 | 3.57 |
|  | y = 135636x + 493223 | 0.9994 |  | 0.5-250 | 0.52 |
| Phorate | y = 34283.8x - 13671.3 | 0.9992 | 0.94 | 0.5-250 | 0.69 |
|  | y = 36585.5x + 41822.3 | 0.9990 |  | 0.5-250 | 0.78 |
| Phorate sulfone | y = 24515.4x + 22000.3 | 0.9991 | 0.44 | 0.5-100 | 1.19 |
|  | y = 55229x + 44723.2 | 0.9995 |  | 0.5-100 | 1.97 |
| Phosalone | y = 1344280x + 499794 | 0.9999 | 1.05 | 0.5-50 | 3.04 |
|  | y = 1281220x + 826217 | 0.9988 |  | 0.5-50 | 0.16 |
| Phosfolan | y = 161270x - 15228.9 | 0.9997 | 0.46 | 0.5-250 | 1.85 |
|  | y = 351640x + 793462 | 0.9993 |  | 0.5-250 | 1.99 |
| Phosmet | y = 146241x - 77796.9 | 0.9989 | 0.78 | 0.5-250 | 4.91 |
|  | y = 187056x + 250378 | 0.9999 |  | 0.5-250 | 1.43 |
| Phosphamidon | y = 90604.5x - 17640 | 0.9995 | 0.70 | 0.5-250 | 1.44 |
|  | y = 128857x - 145106 | 0.9998 |  | 0.5-250 | 1.88 |
| Piperonyl butoxide | y = 339688x + 786950 | 0.9986 | 1.03 | 0.5-250 | 0.21 |
|  | y = 328481x + 511020 | 0.9992 |  | 0.5-250 | 1.87 |
| Pirimicarb | y = 221551x - 7734.98 | 0.9996 | 0.66 | 0.5-250 | 4.72 |
|  | y = 335094x + 1196200 | 0.9990 |  | 0.5-250 | 0.18 |
| Pirimiphos-methyl | y = 320053x + 434766 | 0.9991 | 0.99 | 0.5-250 | 0.25 |
|  | y = 322697x + 282327 | 0.9994 |  | 0.5-250 | 0.22 |
| Prochloraz | y = 171649x + 365749 | 0.9995 | 0.93 | 0.5-250 | 4.18 |
|  | y = 184363x - 38432 | 0.9993 |  | 0.5-250 | 0.81 |
| Profenofos | y = 89950.9x + 291703 | 0.9994 | 0.80 | 0.5-250 | 3.56 |
|  | y = 112112x + 241932 | 0.9991 |  | 0.5-250 | 1.33 |
| Propamocarb | y = 194272x - 75091.2 | 0.9999 | 0.74 | 0.5-250 | 2.31 |
|  | y = 264294x - 325221 | 0.9996 |  | 0.5-250 | 1.38 |
| Propiconazole | y = 95367.3x + 230703 | 0.9996 | 0.93 | 0.5-250 | 1.96 |
|  | y = 102586x + 216541 | 0.9994 |  | 0.5-250 | 0.66 |
| Prothiophos | y = 50573.8x - 157597 | 0.9924 | 1.04 | 1-250 | 1.51 |
|  | y = 48650.7x - 3180.76 | 0.9980 |  | 1-250 | 1.97 |
| Pyraclostrobine | y = 172818x + 400179 | 0.9991 | 0.93 | 0.5-250 | 3.74 |
|  | y = 186375x + 307622 | 0.9992 |  | 0.5-250 | 1.53 |
| Pyridaben | y = 266491x - 80041.8 | 0.9999 | 1.02 | 0.5-250 | 4.87 |
|  | y = 260273x + 50248.3 | 0.9998 |  | 0.5-250 | 0.53 |
| Pyridafenthion | y = 198197x + 1091860 | 0.9955 | 0.97 | 0.5-250 | 4.80 |
|  | y = 204024x + 191747 | 0.9994 |  | 0.5-250 | 0.63 |
| Pyrimethanil | y = 58301.8x + 85136.1 | 0.9996 | 0.89 | 0.5-250 | 0.24 |
|  | y = 65358.3x + 120705 | 0.9994 |  | 0.5-250 | 1.69 |
| Pyrimitate | y = 842196x + 1238750 | 0.9970 | 1.01 | 0.5-100 | 3.40 |
|  | y = 835352x + 732844 | 0.9995 |  | 0.5-100 | 1.12 |
| Pyriproxyfen | y = 363072x + 1202260 | 0.9972 | 1.07 | 0.5-250 | 4.13 |
|  | y = 339296x + 923521 | 0.9990 |  | 0.5-250 | 1.56 |
| Pyrisoxazole | y = 10939x + 54284.2 | 0.9976 | 1.05 | 1-250 | 0.97 |
|  | y = 10389.2x + 31131 | 0.9991 |  | 0.5-250 | 0.30 |
| Quizalofop-p-ethyl | y = 333350x - 257210 | 0.9996 | 1.00 | 0.5-250 | 0.63 |
|  | y = 331894x + 248432 | 0.9993 |  | 0.5-250 | 1.54 |
| Simeconazole | y = 140222x + 45221.7 | 0.9998 | 0.71 | 0.5-250 | 0.91 |
|  | y = 196491x - 29091.1 | 0.9995 |  | 0.5-250 | 1.96 |
| Spirodiclofen | y = 42572x + 23492.6 | 0.9997 | 0.92 | 0.5-250 | 3.45 |
|  | y = 46401.8x + 13914.9 | 0.9998 |  | 0.5-250 | 0.49 |
| Sulfotep | y = 199351x + 198833 | 0.9996 | 0.88 | 0.5-250 | 0.24 |
|  | y = 225425x + 566533 | 0.9994 |  | 0.5-250 | 1.27 |
| Sulprofos | y = 135890x - 264343 | 0.9970 | 0.98 | 0.5-250 | 1.68 |
|  | y = 138275x - 150856 | 0.9992 |  | 0.5-250 | 1.84 |
| Tebuconazole | y = 154232x + 127002 | 0.9993 | 0.96 | 0.5-250 | 1.11 |
|  | y = 160048x + 13926.9 | 0.9998 |  | 0.5-250 | 1.26 |
| Tebufenozide | y = 57827.9x + 75359.9 | 0.9997 | 0.98 | 0.5-250 | 3.43 |
|  | y = 59088.7x + 32806.8 | 0.9999 |  | 0.5-250 | 1.02 |
| Tebufenpyrad | y = 101553x + 16590.6 | 0.9996 | 0.96 | 0.5-250 | 3.63 |
|  | y = 106275x - 191860 | 0.9991 |  | 0.5-250 | 1.57 |
| Temephos | y = 345557x + 1110850 | 0.9979 | 0.99 | 0.5-250 | 3.03 |
|  | y = 347483x + 1162690 | 0.9994 |  | 0.5-250 | 0.71 |
| Terbufos | y = 8763.28x + 32155 | 0.9992 | 1.03 | 0.5-250 | 4.94 |
|  | y = 8518.68x + 33819.1 | 0.9992 |  | 0.5-250 | 1.92 |
| Tetrachlorvinphos | y = 34853.3x + 171712 | 0.9976 | 0.93 | 0.5-250 | 0.93 |
|  | y = 37520.1x + 71579.9 | 0.9998 |  | 0.5-250 | 0.68 |
| Tetraconazole | y = 155157x + 115193 | 0.9981 | 0.90 | 0.5-100 | 3.01 |
|  | y = 172206x + 18684.8 | 0.9996 |  | 0.5-100 | 1.01 |
| Thiacloprid | y = 223298x - 128730 | 0.9996 | 0.54 | 0.5-250 | 1.25 |
|  | y = 416872x + 1377520 | 0.9993 |  | 0.5-250 | 2.00 |
| Thiamethoxam | y = 30676.3x - 25019.3 | 0.9996 | 0.22 | 0.5-250 | 3.67 |
|  | y = 139911x + 4966.57 | 0.9999 |  | 0.5-250 | 1.81 |
| Thiophanate-methyl | y = 67353.8x - 285885 | 0.9948 | 0.20 | 0.5-250 | 0.68 |
|  | y = 338599x + 515079 | 0.9995 |  | 0.5-250 | 1.40 |
| Tolfenpyrad | y = 96813.9x + 259251 | 0.9997 | 1.00 | 0.5-250 | 3.84 |
|  | y = 96485.1x + 162015 | 0.9994 |  | 0.5-250 | 0.99 |
| Triadimefon | y = 45302x + 126042 | 0.9994 | 0.79 | 0.5-250 | 0.43 |
|  | y = 57301.5x + 90066.9 | 0.9994 |  | 0.5-250 | 1.33 |
| Triadimenol | y = 26107.4x + 5564.72 | 0.9997 | 0.94 | 0.5-250 | 2.79 |
|  | y = 27795.9x + 4696.08 | 0.9999 |  | 0.5-250 | 1.77 |
| Tricyclazole | y = 82844.8x - 40560.7 | 0.9999 | 0.35 | 0.5-250 | 4.95 |
|  | y = 234231x + 317911 | 0.9995 |  | 0.5-250 | 1.84 |
| Trifloxystrobin | y = 428439x + 2513340 | 0.9934 | 1.19 | 0.5-250 | 0.19 |
|  | y = 361172x + 1671560 | 0.9984 |  | 0.5-250 | 0.38 |
| Triticonazole | y = 66890.5x - 50047.2 | 0.9997 | 0.66 | 0.5-250 | 1.58 |
|  | y = 100738x - 76418.6 | 0.9994 |  | 0.5-250 | 1.78 |
| Uniconazole | y = 92772.4x + 257855 | 0.9980 | 0.88 | 0.5-250 | 2.16 |
|  | y = 106013x + 353989 | 0.9992 |  | 0.5-250 | 1.52 |
| Vitavax | y = 1205580x + 303798 | 0.9994 | 0.36 | 0.5-50 | 2.11 |
|  | y = 3312810x - 605463 | 0.9996 |  | 0.5-50 | 1.81 |

^1^ Calibration curves are expressed as regression lines (y = a x + b), where y is the peak area and x is the concentration (μg/L), a is the slope, b is the intercept and R is the correlation coefficient.

^2^ The value of slope ratio is calculated by the slope of the matrix-matched calibration curve to the slope of the standard calibration curve in solvent.

^3^ The LOD was the three times of the singal to noise ratio.

^4^ The first calibration curve for each pesticide represents the matrix-matched calibration curve.

^5^ The second calibration curve for each pesticide represents the standard calibration curve in solvent.

Table S8 Recoveries of the pesticides in the spiked PRA-ZJ with the different concentrations of 20 μg/kg, 50 μg/kg and 200 μg/kg.

| Spiked concentration | 20 μg/kg | | 50 μg/kg | | 200 μg/kg | |
| --- | --- | --- | --- | --- | --- | --- |
| Pesticides | Recovery (%) | RSD (%) | Recovery (%) | RSD (%) | Recovery (%) | RSD (%) |
| 3-Hydroxy-carbofuran | -- | -- | 108 | 18.30 | 85.2 | 22.80 |
| Abamectin | -- | -- | 85.7 | 20.00 | 83.9 | 0.82 |
| Acephate | 91.9 | 3.26 | 87.4 | 1.19 | 84.4 | 3.06 |
| Acetamiprid | 106 | 5.31 | 99.5 | 2.39 | 100 | 4.74 |
| Alachlor | 101 | 3.58 | 99.8 | 2.55 | 101 | 4.27 |
| Atrazine | 93.1 | 5.83 | 99.5 | 2.33 | 102 | 2.03 |
| Azaconazole | 104 | 3.28 | 108 | 2.76 | 97.5 | 1.14 |
| Azamethiphos | 108 | 7.17 | 130 | 3.11 | 104 | 3.46 |
| Azoxystrobin | 105 | 0.40 | 112 | 2.57 | 104 | 2.61 |
| Benalaxyl | 104 | 3.13 | 104 | 1.74 | 108 | 3.71 |
| Bendiocarb | 102 | 8.25 | 121 | 2.64 | 101 | 4.29 |
| Bitertanol | 123 | 20.40 | 134 | 8.86 | 98.5 | 1.25 |
| Boscalid | 113 | 2.84 | 108 | 1.07 | 105 | 0.56 |
| Bromuconazole | 110 | 16.00 | 126 | 1.18 | 114 | 2.14 |
| Buprofezin | 103 | 2.81 | 107 | 0.27 | 103 | 1.76 |
| Butachlor | 101 | 0.78 | 103 | 2.80 | 100 | 0.67 |
| Cadusafos | 102 | 3.02 | 102 | 0.09 | 99.9 | 4.03 |
| Carbendazim | 102 | 12.80 | 88.0 | 6.89 | 94.0 | 2.98 |
| Carbofuran | 116 | 5.18 | 113 | 5.21 | 105 | 0.15 |
| Chlorantraniliprole | 124 | 1.39 | 105 | 1.91 | 101 | 4.98 |
| Chlorpyrifos | 97.3 | 2.18 | 97.2 | 4.24 | 101 | 1.44 |
| Chlortoluron | 90.6 | 3.16 | 101 | 0.93 | 99.4 | 2.30 |
| Clothianidin | -- | -- | 86.6 | 14.10 | 94.9 | 7.84 |
| Coumaphos | 107 | 1.52 | 109 | 3.73 | 101 | 2.32 |
| Cyanophenphos | 116 | 3.84 | 108 | 5.06 | 105 | 0.48 |
| Cyazofamid` | 105 | 14.60 | 103 | 4.94 | 119 | 1.04 |
| Cyproconazole | 93.2 | 1.75 | 118 | 3.03 | 101 | 2.64 |
| Cyprodinil | 108 | 4.90 | 105 | 3.00 | 100 | 0.45 |
| Dicofol | 72.7 | 18.00 | 118 | 11.80 | 113 | 2.87 |
| Dicrotophos | -- | -- | 89.0 | 14.70 | 88.2 | 8.80 |
| Difenoconazole | 104 | 3.73 | 105 | 3.43 | 105 | 1.57 |
| Dimethoate | 97.5 | 9.73 | 95.6 | 1.22 | 100 | 1.54 |
| Dimethomorph | 109 | 3.62 | 113 | 1.62 | 101 | 2.12 |
| dimethyl phthalate | 106 | 7.93 | 121 | 4.64 | 108 | 4.70 |
| Diniconazole | 103 | 9.56 | 109 | 2.63 | 103 | 2.86 |
| Dinotefuran | 91.8 | 7.19 | 99.7 | 3.42 | 91.4 | 1.63 |
| Emamectin B1 Benzoate | 91.6 | 1.55 | 96.4 | 0.83 | 91.1 | 1.00 |
| Epoxiconazole | 108 | 8.32 | 103 | 2.28 | 115 | 1.04 |
| Ethiofencarb | 93.1 | 6.51 | 98.3 | 1.08 | 96.1 | 0.67 |
| Etofenprox | 97.3 | 2.54 | 94.8 | 1.01 | 97.9 | 5.46 |
| Etoxazole | 108 | 0.19 | 104 | 1.73 | 101 | 2.02 |
| Fenamiphos | 107 | 5.90 | 102 | 3.34 | 109 | 0.38 |
| Fenamiphos sulfoxide | 108 | 13.30 | 116 | 9.24 | 104 | 2.17 |
| Fenbuconazole | 106 | 6.53 | 111 | 6.49 | 118 | 0.88 |
| Fenpropathrin | 96.5 | 8.99 | 123 | 5.65 | 113 | 0.85 |
| Fipronil | 132 | 15.80 | 94.4 | 7.86 | 106 | 0.45 |
| Fipronil-sulfone | 113 | 10.50 | 101 | 4.66 | 108 | 0.71 |
| Fludioxonil | 76.6 | 15.70 | 102 | 3.42 | 104 | 1.37 |
| Fluopyram | 98.4 | 5.45 | 108 | 3.56 | 104 | 1.64 |
| Fluoxastrobin | 122 | 2.49 | 109 | 0.66 | 109 | 0.57 |
| Flusilazole | 102 | 3.42 | 110 | 2.51 | 112 | 2.01 |
| Flutolanil | 109 | 1.26 | 113 | 1.02 | 104 | 1.97 |
| Flutriafol | 102 | 4.39 | 124 | 7.43 | 94.8 | 1.71 |
| Forchlorfenuron | 97.7 | 3.43 | 115 | 1.40 | 93.3 | 1.18 |
| Hexaconazole | 102 | 1.54 | 109 | 0.22 | 99.2 | 2.61 |
| Hexaflumuron | 109 | 2.12 | 113 | 0.61 | 112 | 1.76 |
| Hexazinone | 99.8 | 4.70 | 108 | 1.95 | 101 | 2.58 |
| Imazalil | 97.4 | 4.23 | 96.0 | 5.52 | 102 | 4.08 |
| Indoxacarb | 113 | 12.10 | 100 | 9.20 | 99.1 | 1.72 |
| Ipconazole | 111 | 6.91 | 104 | 1.90 | 102 | 0.57 |
| Isoprocarb | 87.2 | 2.76 | 112 | 1.47 | 101 | 0.33 |
| Kresoxim-methyl | 114 | 11.60 | 107 | 2.76 | 116 | 3.71 |
| Lufenuron | 78.4 | 10.60 | 118 | 25.10 | 119 | 6.23 |
| Malaoxon | 109 | 4.68 | 109 | 1.67 | 109 | 3.40 |
| Malathion | 107 | 1.24 | 112 | 3.88 | 103 | 1.73 |
| Mefenoxam | 99.9 | 1.19 | 107 | 0.78 | 101 | 2.83 |
| Mepronil | 103 | 3.07 | 112 | 1.27 | 104 | 1.38 |
| Metalaxyl | 105 | 2.60 | 106 | 3.45 | 100 | 4.08 |
| Methidathion | 94.4 | 5.01 | 103 | 4.88 | 102 | 0.85 |
| Methomyl | 97.3 | 13.70 | 85.3 | 16.70 | 92.5 | 3.16 |
| Methoprene | 88.3 | 6.01 | 93.8 | 1.68 | 93.1 | 2.49 |
| Methoxyfenozide | 110 | 4.10 | 111 | 1.80 | 103 | 1.98 |
| Mevinphos | 118 | 8.42 | 123 | 15.30 | 88.5 | 12.20 |
| Monocrotophos | -- | -- | 114 | 20.20 | 104 | 10.20 |
| Myclobutanil | 101 | 1.65 | 113 | 5.23 | 105 | 1.40 |
| Omethoate | 85.6 | 2.06 | 96.2 | 0.77 | 92.1 | 2.02 |
| Paclobutrazol | 112 | 2.07 | 112 | 3.91 | 99.9 | 1.81 |
| Penconazole | 104 | 9.42 | 103 | 1.76 | 104 | 1.89 |
| Phenthoate | 103 | 0.31 | 108 | 3.41 | 102 | 2.77 |
| Phorate | 92.8 | 12.60 | 121 | 8.00 | 98.4 | 1.90 |
| Phorate sulfone | 124 | 9.61 | 98.4 | 4.37 | 103 | 0.94 |
| Phosalone | 108 | 1.66 | 104 | 1.14 | 99.0 | 2.03 |
| Phosfolan | 99.4 | 1.04 | 114 | 1.67 | 99.5 | 2.31 |
| Phosmet | 127 | 4.48 | 145 | 2.65 | 112 | 1.56 |
| Phosphamidon | 97.8 | 10.30 | 93.2 | 1.64 | 100 | 0.23 |
| Piperonyl butoxide | 107 | 2.87 | 104 | 3.00 | 103 | 1.89 |
| Pirimicarb | 103 | 3.33 | 103 | 3.97 | 95.6 | 1.35 |
| Pirimiphos-methyl | 110 | 7.86 | 107 | 1.44 | 100 | 1.41 |
| Prochloraz | 110 | 2.67 | 111 | 2.26 | 105 | 0.16 |
| Profenofos | 99.6 | 5.53 | 121 | 0.79 | 99.0 | 1.41 |
| Propamocarb | 71.5 | 5.28 | 62.7 | 4.40 | 71.1 | 0.51 |
| Propiconazole | 100 | 2.08 | 113 | 3.73 | 99.1 | 2.30 |
| Prothiophos | 102 | 10.60 | 95.6 | 1.83 | 92.9 | 2.26 |
| Pyraclostrobine | 101 | 2.58 | 106 | 1.33 | 101 | 2.40 |
| Pyridaben | 87.7 | 1.00 | 93.9 | 1.78 | 87.5 | 2.15 |
| Pyridafenthion | 119 | 2.27 | 113 | 2.05 | 108 | 3.87 |
| Pyrimethanil | 113 | 3.45 | 112 | 1.90 | 107 | 1.30 |
| Pyrimitate | 101 | 1.76 | 107 | 1.57 | 101 | 1.32 |
| Pyriproxyfen | 93.2 | 1.39 | 100 | 1.49 | 104 | 1.68 |
| Pyrisoxazole | 120 | 21.00 | 126 | 5.55 | 104 | 5.89 |
| Quizalofop-p-ethyl | 105 | 2.22 | 105 | 0.97 | 105 | 1.20 |
| Simeconazole | 110 | 2.09 | 112 | 5.30 | 114 | 1.47 |
| Spirodiclofen | 100 | 12.70 | 106 | 3.76 | 92.1 | 2.89 |
| Sulfotep | 122 | 13.70 | 114 | 1.22 | 131 | 22.20 |
| Sulprofos | 88.7 | 8.97 | 98.7 | 0.82 | 103 | 2.27 |
| Tebuconazole | 99.9 | 2.92 | 102 | 1.31 | 102 | 1.23 |
| Tebufenozide | 96.4 | 5.93 | 108 | 2.33 | 101 | 1.19 |
| Tebufenpyrad | 102 | 5.63 | 99.6 | 0.97 | 101 | 0.92 |
| Temephos | 104 | 1.18 | 106 | 0.81 | 98.7 | 0.65 |
| Terbufos | -- | -- | 88.0 | 4.35 | 105 | 8.59 |
| Tetrachlorvinphos | 99.4 | 13.40 | 104 | 5.28 | 102 | 1.71 |
| Tetraconazole | 97.0 | 7.00 | 114 | 4.25 | 118 | 2.24 |
| Thiacloprid | 91.0 | 6.98 | 103 | 3.71 | 98.2 | 2.44 |
| Thiamethoxam | 96.7 | 3.44 | 93.9 | 8.27 | 97.2 | 3.37 |
| Thiophanate-methyl | 113 | 0.43 | 106 | 9.30 | 103 | 3.33 |
| Tolfenpyrad | 101 | 2.34 | 109 | 2.86 | 96.3 | 4.19 |
| Triadimefon | 97.4 | 1.24 | 109 | 2.25 | 99.0 | 5.08 |
| Triadimenol | 105 | 6.53 | 105 | 2.92 | 108 | 1.09 |
| Tricyclazole | 101 | 4.91 | 96.8 | 1.51 | 91.4 | 1.18 |
| Trifloxystrobin | 107 | 1.53 | 104 | 1.15 | 104 | 1.74 |
| Triticonazole | 105 | 8.20 | 109 | 5.08 | 114 | 3.60 |
| Uniconazole | 109 | 2.56 | 111 | 2.22 | 103 | 0.91 |
| Vitavax | 86.8 | 4.43 | 102 | 2.86 | 97.5 | 2.70 |

Table S9 Recoveries of the pesticides in the spiked PRA-AH with the different concentrations of 20 μg/kg, 50 μg/kg and 200 μg/kg.

| Spiked concentration | 20 μg/kg | | 50 μg/kg | | 200 μg/kg | |
| --- | --- | --- | --- | --- | --- | --- |
| Pesticides | Recovery (%) | RSD (%) | Recovery (%) | RSD (%) | Recovery (%) | RSD (%) |
| 3-Hydroxy-carbofuran | -- | -- | -- | -- | 103 | 1.30 |
| Abamectin | -- | -- | -- | -- | 85.6 | 2.49 |
| Acephate | 83.8 | 0.46 | 95.2 | 5.73 | 83.9 | 1.11 |
| Acetamiprid | 87.8 | 13.80 | 106 | 2.29 | 93.0 | 2.94 |
| Alachlor | 99.9 | 4.03 | 112 | 2.54 | 102 | 1.52 |
| Atrazine | 113 | 2.65 | 116 | 1.45 | 99.5 | 1.21 |
| Azaconazole | 107 | 0.59 | 118 | 3.31 | 99.3 | 3.26 |
| Azamethiphos | 104 | 0.47 | 137 | 4.20 | 106 | 1.38 |
| Azoxystrobin | 108 | 1.92 | 114 | 1.87 | 105 | 1.27 |
| Benalaxyl | 102 | 2.22 | 119 | 2.15 | 102 | 0.88 |
| Bendiocarb | -- | -- | 99.2 | 2.03 | 100 | 1.73 |
| Bitertanol | -- | -- | 100 | 13.70 | 87.1 | 1.79 |
| Boscalid | 115 | 0.51 | 114 | 1.72 | 106 | 2.14 |
| Bromuconazole | 121 | 1.22 | 111 | 7.89 | 106 | 2.24 |
| Buprofezin | 104 | 2.01 | 111 | 1.80 | 102 | 1.60 |
| Butachlor | 99.6 | 2.31 | 112 | 1.56 | 105 | 0.35 |
| Cadusafos | 104 | 1.21 | 117 | 2.78 | 99.2 | 0.55 |
| Carbendazim | 77.5 | 6.69 | 93.1 | 3.76 | 88.0 | 3.36 |
| Carbofuran | 85.8 | 0.61 | 129 | 6.32 | 109 | 2.08 |
| Chlorantraniliprole | 102 | 8.77 | 103 | 2.61 | 101 | 0.55 |
| Chlorpyrifos | 107 | 1.43 | 110 | 0.85 | 102 | 0.43 |
| Chlortoluron | 101 | 4.55 | 107 | 5.24 | 93.5 | 1.35 |
| Clothianidin | -- | -- | 98.3 | 16.90 | 85.4 | 5.49 |
| Coumaphos | 99.0 | 5.62 | 117 | 1.30 | 102 | 1.58 |
| Cyanophenphos | 112 | 8.15 | 116 | 7.38 | 98.1 | 6.54 |
| Cyazofamid` | 105 | 6.12 | 116 | 9.05 | 95.5 | 7.96 |
| Cyproconazole | 103 | 5.80 | 106 | 5.23 | 98.4 | 3.34 |
| Cyprodinil | 106 | 6.48 | 108 | 4.90 | 101 | 2.42 |
| Dicofol | 125 | 6.72 | 93.6 | 17.20 | 102 | 2.64 |
| Dicrotophos | -- | -- | 101 | 6.71 | 89.0 | 7.19 |
| Difenoconazole | 99.1 | 8.78 | 121 | 1.75 | 98.7 | 0.67 |
| Dimethoate | 88.0 | 6.09 | 118 | 0.95 | 98.0 | 1.18 |
| Dimethomorph | 112 | 1.14 | 116 | 1.73 | 103 | 0.83 |
| dimethyl phthalate | 95.9 | 0.40 | 100 | 1.67 | 97.5 | 3.93 |
| Diniconazole | 104 | 4.62 | 106 | 1.34 | 100 | 2.74 |
| Dinotefuran | 90.1 | 6.29 | 103 | 4.12 | 92.1 | 2.37 |
| Emamectin B1 Benzoate | 99.1 | 7.03 | 101 | 1.95 | 93.7 | 2.03 |
| Epoxiconazole | 108 | 4.44 | 121 | 12.20 | 98.8 | 3.52 |
| Ethiofencarb | 91.8 | 0.62 | 100 | 1.44 | 92.7 | 0.62 |
| Etofenprox | 111 | 2.81 | 101 | 0.86 | 102 | 3.45 |
| Etoxazole | 104 | 0.16 | 109 | 1.39 | 98.3 | 0.80 |
| Fenamiphos | 102 | 0.61 | 113 | 7.77 | 97.1 | 4.41 |
| Fenamiphos sulfoxide | 126 | 18.50 | 115 | 5.42 | 106 | 1.62 |
| Fenbuconazole | 105 | 4.20 | 110 | 11.10 | 96.3 | 5.85 |
| Fenpropathrin | 123 | 11.50 | 145 | 1.87 | 101 | 4.24 |
| Fipronil | 101 | 2.07 | 92.7 | 0.48 | 113 | 4.06 |
| Fipronil-sulfone | 109 | 2.72 | 110 | 1.41 | 98.7 | 2.45 |
| Fludioxonil | 100 | 9.23 | 115 | 1.04 | 103 | 1.29 |
| Fluopyram | 107 | 5.44 | 119 | 4.73 | 96.6 | 2.71 |
| Fluoxastrobin | 112 | 4.07 | 112 | 6.97 | 97.2 | 2.42 |
| Flusilazole | 107 | 1.84 | 113 | 1.90 | 95.9 | 3.78 |
| Flutolanil | 101 | 2.65 | 117 | 1.98 | 105 | 1.02 |
| Flutriafol | 97.2 | 1.27 | 106 | 4.35 | 96.0 | 0.96 |
| Forchlorfenuron | 96.0 | 2.13 | 108 | 0.69 | 95.6 | 4.70 |
| Hexaconazole | 95.3 | 3.30 | 113 | 3.03 | 97.5 | 0.86 |
| Hexaflumuron | 101 | 3.53 | 122 | 3.30 | 108 | 0.49 |
| Hexazinone | 114 | 2.39 | 115 | 1.23 | 100 | 1.05 |
| Imazalil | 101 | 0.42 | 122 | 3.86 | 103 | 2.00 |
| Indoxacarb | 111 | 17.70 | 107 | 8.39 | 102 | 3.49 |
| Ipconazole | 102 | 3.69 | 106 | 4.38 | 98.8 | 3.63 |
| Isoprocarb | 89.6 | 3.57 | 118 | 2.52 | 95.5 | 0.74 |
| Kresoxim-methyl | 98.1 | 5.67 | 131 | 3.02 | 101 | 3.91 |
| Lufenuron | 119 | 17.80 | 110 | 1.26 | 107 | 1.07 |
| Malaoxon | 96.5 | 2.72 | 119 | 3.85 | 106 | 0.54 |
| Malathion | 115 | 4.31 | 123 | 4.64 | 101 | 2.15 |
| Mefenoxam | 103 | 0.21 | 119 | 1.32 | 100 | 0.25 |
| Mepronil | 109 | 2.72 | 114 | 1.82 | 103 | 0.76 |
| Metalaxyl | 98.5 | 2.94 | 110 | 3.68 | 98.7 | 0.15 |
| Methidathion | 108 | 2.71 | 103 | 8.71 | 104 | 1.76 |
| Methomyl | 89.5 | 11.60 | 115 | 1.59 | 99.4 | 4.57 |
| Methoprene | 127 | 4.12 | 124 | 3.42 | 101 | 5.19 |
| Methoxyfenozide | 96.2 | 8.33 | 109 | 4.64 | 100 | 0.48 |
| Mevinphos | 111 | 9.88 | 116 | 7.06 | 99.3 | 5.15 |
| Monocrotophos | -- | -- | -- | -- | 96.8 | 4.11 |
| Myclobutanil | 115 | 1.27 | 118 | 4.24 | 105 | 4.15 |
| Omethoate | 92.5 | 1.38 | 100 | 2.36 | 92.5 | 1.47 |
| Paclobutrazol | 101 | 8.37 | 112 | 1.00 | 106 | 4.60 |
| Penconazole | 120 | 10.80 | 111 | 2.72 | 101 | 1.25 |
| Phenthoate | 121 | 0.10 | 118 | 1.89 | 104 | 1.98 |
| Phorate | 98.4 | 1.80 | 121 | 3.90 | 157 | 30.10 |
| Phorate sulfone | 113 | 1.41 | 115 | 1.98 | 104 | 0.88 |
| Phosalone | 108 | 3.13 | 115 | 1.38 | 92.8 | 1.72 |
| Phosfolan | 92.9 | 1.93 | 114 | 1.41 | 95.6 | 0.90 |
| Phosmet | -- | -- | -- | -- | 111 | 0.32 |
| Phosphamidon | 97.7 | 10.90 | 114 | 4.60 | 94.8 | 1.42 |
| Piperonyl butoxide | 104 | 3.64 | 111 | 1.12 | 102 | 2.17 |
| Pirimicarb | 97.4 | 0.69 | 109 | 0.59 | 100 | 1.72 |
| Pirimiphos-methyl | 111 | 17.20 | 111 | 0.78 | 106 | 1.44 |
| Prochloraz | 104 | 8.60 | 103 | 0.63 | 99.5 | 2.20 |
| Profenofos | 102 | 10.80 | 111 | 4.05 | 101 | 0.77 |
| Propamocarb | 73.6 | 3.85 | 70.0 | 4.93 | 68.7 | 1.91 |
| Propiconazole | 108 | 8.07 | 110 | 1.28 | 97.8 | 2.95 |
| Prothiophos | 111 | 5.38 | 112 | 4.42 | 98.4 | 1.67 |
| Pyraclostrobine | 113 | 3.72 | 112 | 0.74 | 104 | 2.38 |
| Pyridaben | 102 | 1.68 | 106 | 4.05 | 96.7 | 1.47 |
| Pyridafenthion | 112 | 3.18 | 119 | 6.78 | 94.2 | 1.98 |
| Pyrimethanil | 115 | 5.69 | 118 | 1.50 | 107 | 0.65 |
| Pyrimitate | 107 | 4.27 | 111 | 0.67 | 101 | 1.19 |
| Pyriproxyfen | 106 | 2.04 | 109 | 1.09 | 98.6 | 0.49 |
| Pyrisoxazole | -- | -- | 120 | 4.25 | 105 | 2.19 |
| Quizalofop-p-ethyl | 107 | 0.55 | 111 | 1.77 | 99.3 | 1.48 |
| Simeconazole | 118 | 2.59 | 112 | 9.51 | 99.2 | 4.42 |
| Spirodiclofen | 103 | 13.30 | 110 | 4.75 | 100 | 7.01 |
| Sulfotep | 116 | 16.40 | 116 | 1.59 | 89.9 | 13.40 |
| Sulprofos | 103 | 2.99 | 111 | 1.05 | 100 | 1.46 |
| Tebuconazole | 104 | 3.89 | 107 | 2.13 | 99.3 | 1.19 |
| Tebufenozide | 107 | 6.82 | 115 | 3.64 | 90.0 | 4.95 |
| Tebufenpyrad | 100 | 5.17 | 106 | 2.64 | 101 | 1.46 |
| Temephos | 103 | 1.85 | 113 | 1.86 | 96.8 | 4.37 |
| Terbufos | 104 | 15.20 | 95.0 | 9.86 | 100 | 5.13 |
| Tetrachlorvinphos | 97.5 | 6.70 | 119 | 16.10 | 98.9 | 1.09 |
| Tetraconazole | 102 | 1.57 | 116 | 6.74 | 102 | 5.52 |
| Thiacloprid | 88.3 | 4.82 | 105 | 3.62 | 98.5 | 1.50 |
| Thiamethoxam | 88.2 | 6.01 | 103 | 5.50 | 89.4 | 2.60 |
| Thiophanate-methyl | 86.0 | 11.20 | 109 | 4.63 | 104 | 1.10 |
| Tolfenpyrad | 95.2 | 5.54 | 104 | 5.25 | 97.5 | 2.44 |
| Triadimefon | 93.0 | 8.34 | 125 | 0.43 | 102 | 1.15 |
| Triadimenol | 110 | 10.80 | 109 | 5.58 | 107 | 3.68 |
| Tricyclazole | 87.1 | 4.51 | 89.5 | 2.41 | 91.0 | 3.65 |
| Trifloxystrobin | 102 | 0.75 | 112 | 0.71 | 102 | 1.76 |
| Triticonazole | 106 | 5.36 | 114 | 5.14 | 95.1 | 7.25 |
| Uniconazole | 98.0 | 5.39 | 109 | 2.54 | 96.2 | 7.77 |
| Vitavax | 90.2 | 4.95 | 106 | 4.20 | 97.1 | 0.73 |

Table S10 Recoveries of the pesticides in the spiked PRA-SD with the different concentrations of 20 μg/kg, 50 μg/kg and 200 μg/kg.

| Spiked concentration | 20 μg/kg | | 50 μg/kg | | 200 μg/kg | |
| --- | --- | --- | --- | --- | --- | --- |
| Pesticides | Recovery (%) | RSD (%) | Recovery (%) | RSD (%) | Recovery (%) | RSD (%) |
| 3-Hydroxy-carbofuran | 119 | 7.28 | 109 | 14.50 | 97.3 | 10.30 |
| Abamectin | -- | -- | -- | -- | 92.1 | 8.48 |
| Acephate | 84.2 | 2.53 | 92.4 | 2.83 | 84.5 | 0.33 |
| Acetamiprid | 87.4 | 4.28 | 106 | 1.82 | 92.9 | 1.22 |
| Alachlor | 99.9 | 3.61 | 107 | 3.12 | 99.5 | 2.17 |
| Atrazine | 93.3 | 4.82 | 102 | 1.74 | 96.9 | 1.57 |
| Azaconazole | 91.1 | 1.85 | 119 | 2.84 | 98.6 | 0.55 |
| Azamethiphos | 111 | 5.93 | 123 | 1.02 | 99.4 | 3.55 |
| Azoxystrobin | 102 | 0.56 | 110 | 1.13 | 94.9 | 1.17 |
| Benalaxyl | 104 | 8.17 | 114 | 1.55 | 102 | 2.56 |
| Bendiocarb | -- | -- | 86.0 | 3.40 | 100 | 2.97 |
| Bitertanol | 122 | 6.07 | 128 | 3.34 | 93.2 | 0.49 |
| Boscalid | 103 | 1.36 | 105 | 3.24 | 97.5 | 0.38 |
| Bromuconazole | 104 | 8.60 | 111 | 0.83 | 113 | 4.92 |
| Buprofezin | 103 | 2.23 | 107 | 1.83 | 101 | 1.63 |
| Butachlor | 107 | 0.89 | 109 | 2.06 | 101 | 1.38 |
| Cadusafos | 101 | 1.10 | 106 | 0.70 | 98.3 | 2.53 |
| Carbendazim | 90.3 | 3.27 | 78.6 | 3.78 | 87.6 | 2.95 |
| Carbofuran | -- | -- | 111 | 7.16 | 104 | 0.15 |
| Chlorantraniliprole | 95.2 | 3.98 | 104 | 1.57 | 95.8 | 4.80 |
| Chlorpyrifos | 100 | 4.48 | 108 | 0.43 | 104 | 0.48 |
| Chlortoluron | 91.0 | 2.72 | 100 | 2.78 | 94.9 | 1.99 |
| Clothianidin | -- | -- | -- | -- | 82.7 | 6.11 |
| Coumaphos | 108 | 3.99 | 108 | 2.29 | 107 | 0.79 |
| Cyanophenphos | 88.0 | 7.57 | 117 | 3.13 | 111 | 6.64 |
| Cyazofamid` | -- | -- | 128 | 0.71 | 132 | 4.96 |
| Cyproconazole | 108 | 2.73 | 113 | 9.00 | 106 | 1.41 |
| Cyprodinil | 101 | 3.04 | 103 | 1.76 | 102 | 1.99 |
| Dicofol | 78.7 | 9.62 | 114 | 9.71 | 92.0 | 5.53 |
| Dicrotophos | -- | -- | -- | -- | 100 | 9.10 |
| Difenoconazole | 92.8 | 5.17 | 98.6 | 1.92 | 96.4 | 2.00 |
| Dimethoate | 94.2 | 11.60 | 102 | 4.54 | 97.8 | 6.08 |
| Dimethomorph | 103 | 2.65 | 106 | 5.07 | 100 | 1.19 |
| dimethyl phthalate | 106 | 0.80 | 103 | 4.62 | 93.2 | 2.55 |
| Diniconazole | 115 | 6.82 | 104 | 0.33 | 96.3 | 1.16 |
| Dinotefuran | 94.2 | 11.20 | 107 | 7.20 | 87.5 | 3.28 |
| Emamectin B1 Benzoate | 96.3 | 4.47 | 94.5 | 1.90 | 92.2 | 3.28 |
| Epoxiconazole | -- | -- | 128 | 5.69 | 117 | 0.65 |
| Ethiofencarb | 92.5 | 3.17 | 105 | 0.98 | 96.6 | 1.48 |
| Etofenprox | 103 | 3.65 | 109 | 1.52 | 95.4 | 1.38 |
| Etoxazole | 104 | 2.32 | 102 | 0.75 | 98.4 | 1.18 |
| Fenamiphos | 100 | 7.90 | 126 | 6.14 | 110 | 2.29 |
| Fenamiphos sulfoxide | -- | -- | 102 | 6.20 | 96.9 | 2.96 |
| Fenbuconazole | 110 | 5.03 | 124 | 4.28 | 118 | 3.96 |
| Fenpropathrin | 93.5 | 4.86 | 87.1 | 5.98 | 84.8 | 7.12 |
| Fipronil | -- | -- | -- | -- | 100 | 3.93 |
| Fipronil-sulfone | 104 | 7.06 | 108 | 5.44 | 98.6 | 6.09 |
| Fludioxonil | -- | -- | 110 | 1.40 | 108 | 1.61 |
| Fluopyram | 109 | 10.10 | 114 | 1.89 | 108 | 2.12 |
| Fluoxastrobin | 104 | 4.04 | 122 | 0.08 | 102 | 6.18 |
| Flusilazole | 105 | 3.30 | 123 | 3.97 | 117 | 3.01 |
| Flutolanil | 108 | 0.85 | 105 | 1.33 | 98.1 | 1.94 |
| Flutriafol | 98.4 | 2.22 | 102 | 5.61 | 101 | 4.93 |
| Forchlorfenuron | 103 | 5.04 | 100 | 2.17 | 96.5 | 1.21 |
| Hexaconazole | 98.4 | 4.46 | 104 | 0.20 | 102 | 3.45 |
| Hexaflumuron | 109 | 4.11 | 114 | 1.68 | 105 | 4.97 |
| Hexazinone | 95.2 | 4.84 | 104 | 5.03 | 97.0 | 4.61 |
| Imazalil | 95.5 | 7.89 | 114 | 6.87 | 98.1 | 0.69 |
| Indoxacarb | 78.0 | 6.97 | 116 | 2.90 | 90.4 | 1.18 |
| Ipconazole | 103 | 5.03 | 104 | 4.29 | 96.2 | 2.03 |
| Isoprocarb | 101 | 7.45 | 100 | 0.61 | 101 | 1.97 |
| Kresoxim-methyl | 115 | 6.26 | 124 | 2.90 | 112 | 1.11 |
| Lufenuron | 110 | 10.90 | 115 | 6.62 | 129 | 7.39 |
| Malaoxon | 104 | 3.46 | 107 | 1.57 | 98.7 | 3.90 |
| Malathion | 108 | 0.36 | 113 | 2.05 | 103 | 3.41 |
| Mefenoxam | 101 | 5.60 | 113 | 2.63 | 96.0 | 1.82 |
| Mepronil | 104 | 4.27 | 111 | 1.75 | 104 | 2.85 |
| Metalaxyl | 104 | 0.71 | 111 | 1.17 | 100 | 1.89 |
| Methidathion | 111 | 2.02 | 114 | 3.85 | 100 | 4.13 |
| Methomyl | 77.2 | 6.25 | 95.5 | 12.60 | 94.2 | 5.38 |
| Methoprene | 93.6 | 3.93 | 98.5 | 3.80 | 94.3 | 1.67 |
| Methoxyfenozide | 107 | 2.90 | 105 | 0.65 | 96.3 | 2.48 |
| Mevinphos | 96.7 | 4.84 | 99.3 | 7.05 | 83.2 | 1.51 |
| Monocrotophos | 105 | 9.40 | 104 | 7.98 | 103 | 1.28 |
| Myclobutanil | 113 | 6.13 | 100 | 3.05 | 101 | 1.11 |
| Omethoate | 87.2 | 2.31 | 93.5 | 3.56 | 96.0 | 3.70 |
| Paclobutrazol | 108 | 0.42 | 105 | 3.53 | 94.0 | 2.68 |
| Penconazole | 107 | 10.20 | 107 | 1.23 | 105 | 0.29 |
| Phenthoate | 105 | 5.88 | 111 | 2.94 | 112 | 0.83 |
| Phorate | -- | -- | 108 | 3.86 | 121 | 11.80 |
| Phorate sulfone | 112 | 7.51 | 110 | 7.34 | 95.0 | 2.17 |
| Phosalone | 105 | 1.98 | 111 | 1.45 | 103 | 3.00 |
| Phosfolan | 95.9 | 3.62 | 106 | 1.75 | 95.5 | 0.53 |
| Phosmet | -- | -- | -- | -- | 112 | 1.93 |
| Phosphamidon | 112 | 4.67 | 120 | 1.66 | 96.8 | 0.49 |
| Piperonyl butoxide | 103 | 3.22 | 109 | 0.28 | 102 | 0.53 |
| Pirimicarb | 95.2 | 0.62 | 105 | 2.65 | 96.8 | 2.44 |
| Pirimiphos-methyl | 71.4 | 5.98 | 110 | 1.92 | 98.5 | 2.17 |
| Prochloraz | 109 | 1.36 | 112 | 4.61 | 101 | 0.62 |
| Profenofos | 97.0 | 9.75 | 105 | 6.15 | 101 | 5.69 |
| Propamocarb | 73.7 | 6.66 | 64.9 | 1.70 | 72.7 | 1.84 |
| Propiconazole | 97.2 | 3.92 | 100 | 3.09 | 100 | 0.30 |
| Prothiophos | 101 | 4.53 | 102 | 1.69 | 93.2 | 1.72 |
| Pyraclostrobine | 112 | 0.12 | 105 | 1.41 | 102 | 3.50 |
| Pyridaben | 99.0 | 2.28 | 103 | 1.87 | 94.4 | 2.28 |
| Pyridafenthion | 103 | 1.43 | 109 | 0.65 | 104 | 2.54 |
| Pyrimethanil | 108 | 4.15 | 95.7 | 5.25 | 98.4 | 3.21 |
| Pyrimitate | 101 | 3.13 | 108 | 0.98 | 105 | 2.99 |
| Pyriproxyfen | 103 | 5.01 | 110 | 2.99 | 106 | 1.36 |
| Pyrisoxazole | 90.0 | 14.20 | 91.2 | 2.44 | 107 | 5.97 |
| Quizalofop-p-ethyl | 101 | 2.31 | 106 | 2.06 | 99.2 | 2.11 |
| Simeconazole | 102 | 5.66 | 122 | 8.18 | 112 | 0.85 |
| Spirodiclofen | 105 | 4.85 | 103 | 4.22 | 94.8 | 2.12 |
| Sulfotep | 77.0 | 7.79 | 128 | 8.40 | 97.4 | 9.02 |
| Sulprofos | 100 | 3.47 | 112 | 3.05 | 94.2 | 3.32 |
| Tebuconazole | 102 | 4.49 | 103 | 0.14 | 106 | 2.87 |
| Tebufenozide | 105 | 3.80 | 117 | 1.42 | 106 | 3.94 |
| Tebufenpyrad | 114 | 1.74 | 104 | 0.71 | 102 | 3.15 |
| Temephos | 107 | 1.28 | 107 | 1.60 | 96.3 | 1.97 |
| Terbufos | 120 | 5.48 | 111 | 1.85 | 99.1 | 6.97 |
| Tetrachlorvinphos | 111 | 15.00 | 110 | 3.25 | 111 | 1.44 |
| Tetraconazole | 99.8 | 10.20 | 122 | 3.56 | 105 | 5.51 |
| Thiacloprid | 84.9 | 6.10 | 97.4 | 0.72 | 93.8 | 1.21 |
| Thiamethoxam | -- | -- | 77.3 | 10.90 | 76.8 | 4.11 |
| Thiophanate-methyl | 84.7 | 2.44 | 146 | 1.10 | 101 | 5.42 |
| Tolfenpyrad | 110 | 9.88 | 99.4 | 2.55 | 99.7 | 1.55 |
| Triadimefon | 107 | 2.03 | 119 | 1.15 | 103 | 3.35 |
| Triadimenol | 103 | 9.14 | 116 | 2.62 | 110 | 3.75 |
| Tricyclazole | 97.4 | 5.80 | 98.8 | 5.18 | 89.2 | 1.39 |
| Trifloxystrobin | 105 | 5.41 | 106 | 0.43 | 101 | 1.11 |
| Triticonazole | 102 | 5.07 | 113 | 10.60 | 116 | 5.44 |
| Uniconazole | 107 | 4.23 | 116 | 6.72 | 107 | 1.28 |
| Vitavax | 90.9 | 5.57 | 106 | 1.30 | 91.1 | 0.92 |

Table S11 Recoveries of the pesticides in the spiked PRA-HN with the different concentrations of 20 μg/kg, 50 μg/kg and 200 μg/kg.

| Spiked concentration | 20 μg/kg | | 50 μg/kg | | 200 μg/kg | |
| --- | --- | --- | --- | --- | --- | --- |
| Pesticides | Recovery (%) | RSD (%) | Recovery (%) | RSD (%) | Recovery (%) | RSD (%) |
| 3-Hydroxy-carbofuran | -- | -- | 92.9 | 10.90 | 115 | 2.35 |
| Abamectin | -- | -- | 120 | 6.58 | 105 | 3.71 |
| Acephate | 87.8 | 1.45 | 83.5 | 1.89 | 84.6 | 3.76 |
| Acetamiprid | 94.7 | 5.15 | 101 | 3.45 | 98.3 | 2.11 |
| Alachlor | 104 | 0.91 | 112 | 1.97 | 113 | 1.21 |
| Atrazine | 105 | 0.99 | 112 | 1.94 | 97.2 | 0.86 |
| Azaconazole | 116 | 0.58 | 102 | 0.92 | 109 | 2.58 |
| Azamethiphos | 105 | 4.77 | 112 | 3.11 | 109 | 1.64 |
| Azoxystrobin | 97.6 | 3.37 | 94.7 | 2.10 | 112 | 5.02 |
| Benalaxyl | 107 | 1.66 | 117 | 3.86 | 115 | 6.28 |
| Bendiocarb | -- | -- | 93.7 | 9.95 | 104 | 1.85 |
| Bitertanol | -- | -- | 98.8 | 14.10 | 106 | 2.79 |
| Boscalid | 109 | 1.83 | 95.1 | 5.22 | 107 | 4.86 |
| Bromuconazole | 109 | 3.29 | 106 | 8.22 | 115 | 4.44 |
| Buprofezin | 107 | 2.50 | 119 | 1.94 | 106 | 1.71 |
| Butachlor | 106 | 2.64 | 118 | 0.44 | 109 | 0.54 |
| Cadusafos | 106 | 2.44 | 104 | 3.78 | 113 | 4.06 |
| Carbendazim | 88.3 | 1.58 | 84.1 | 2.48 | 91.3 | 1.45 |
| Carbofuran | 101 | 4.48 | 105 | 1.89 | 107 | 2.73 |
| Chlorantraniliprole | 85.2 | 2.39 | 95.1 | 6.98 | 112 | 3.01 |
| Chlorpyrifos | 106 | 0.18 | 100 | 4.98 | 110 | 1.35 |
| Chlortoluron | 92.4 | 0.14 | 107 | 1.73 | 99.4 | 2.62 |
| Clothianidin | -- | -- | 122 | 9.76 | 84.8 | 2.57 |
| Coumaphos | 110 | 2.82 | 99.1 | 3.36 | 110 | 1.96 |
| Cyanophenphos | 129 | 7.61 | 112 | 2.29 | 122 | 6.76 |
| Cyazofamid` | 126 | 12.60 | 124 | 3.28 | 115 | 10.60 |
| Cyproconazole | 106 | 3.64 | 103 | 3.73 | 107 | 5.67 |
| Cyprodinil | 92.4 | 9.54 | 108 | 2.19 | 109 | 2.67 |
| Dicofol | 95.9 | 3.84 | 106 | 13.80 | 108 | 6.24 |
| Dicrotophos | 108 | 15.50 | 98.3 | 5.44 | 90.5 | 2.98 |
| Difenoconazole | 111 | 0.15 | 101 | 1.33 | 108 | 0.85 |
| Dimethoate | 101 | 6.29 | 104 | 4.82 | 92.4 | 0.46 |
| Dimethomorph | 108 | 1.85 | 103 | 1.06 | 114 | 4.15 |
| dimethyl phthalate | 97.6 | 4.63 | 95.9 | 0.86 | 97.3 | 0.35 |
| Diniconazole | 102 | 3.73 | 102 | 1.16 | 118 | 4.01 |
| Dinotefuran | 87.5 | 0.71 | 99.8 | 1.05 | 95.9 | 1.56 |
| Emamectin B1 Benzoate | 93.9 | 0.70 | 101 | 1.89 | 94.3 | 2.14 |
| Epoxiconazole | 118 | 7.31 | 120 | 0.85 | 116 | 3.10 |
| Ethiofencarb | 97.1 | 0.29 | 90.4 | 3.85 | 97.3 | 0.28 |
| Etofenprox | 107 | 10.30 | 137 | 5.16 | 97.7 | 0.68 |
| Etoxazole | 108 | 0.95 | 113 | 2.09 | 102 | 1.49 |
| Fenamiphos | 105 | 2.65 | 95.6 | 2.91 | 108 | 4.88 |
| Fenamiphos sulfoxide | 92.0 | 0.60 | 99.4 | 0.59 | 109 | 2.81 |
| Fenbuconazole | 127 | 8.15 | 106 | 4.30 | 118 | 0.50 |
| Fenpropathrin | 85.0 | 13.10 | 101 | 9.95 | 76.9 | 12.00 |
| Fipronil | 120 | 12.00 | 122 | 8.43 | 109 | 12.50 |
| Fipronil-sulfone | 108 | 3.18 | 123 | 5.39 | 117 | 4.11 |
| Fludioxonil | 110 | 3.50 | 92.0 | 4.73 | 110 | 2.02 |
| Fluopyram | 109 | 3.47 | 117 | 2.26 | 107 | 2.26 |
| Fluoxastrobin | 109 | 5.92 | 111 | 0.21 | 103 | 0.78 |
| Flusilazole | 115 | 3.19 | 124 | 8.06 | 108 | 2.04 |
| Flutolanil | 101 | 2.19 | 102 | 11.60 | 114 | 5.49 |
| Flutriafol | 104 | 9.76 | 92.0 | 0.71 | 104 | 1.11 |
| Forchlorfenuron | 106 | 3.68 | 105 | 9.43 | 112 | 3.80 |
| Hexaconazole | 116 | 0.92 | 117 | 3.17 | 110 | 2.94 |
| Hexaflumuron | 103 | 9.12 | 117 | 3.50 | 105 | 2.07 |
| Hexazinone | 95.9 | 1.42 | 116 | 2.85 | 100 | 3.87 |
| Imazalil | 94.2 | 3.96 | 107 | 2.92 | 101 | 5.67 |
| Indoxacarb | -- | -- | 104 | 5.60 | 116 | 3.51 |
| Ipconazole | 104 | 2.06 | 104 | 0.29 | 107 | 1.07 |
| Isoprocarb | 103 | 1.38 | 113 | 2.31 | 95.5 | 1.29 |
| Kresoxim-methyl | 88.5 | 8.41 | 115 | 8.10 | 118 | 2.38 |
| Lufenuron | 107 | 4.99 | 112 | 1.02 | 109 | 1.44 |
| Malaoxon | 97.0 | 0.66 | 146 | 3.60 | 93.8 | 1.82 |
| Malathion | 94.4 | 2.31 | 106 | 4.81 | 116 | 4.68 |
| Mefenoxam | 97.6 | 2.91 | 105 | 2.71 | 111 | 0.42 |
| Mepronil | 98.4 | 1.27 | 105 | 5.39 | 101 | 0.46 |
| Metalaxyl | 98.5 | 1.05 | 101 | 0.33 | 107 | 3.86 |
| Methidathion | 107 | 0.68 | 118 | 4.85 | 109 | 5.36 |
| Methomyl | 92.4 | 3.00 | 91.5 | 3.06 | 99.3 | 3.96 |
| Methoprene | 85.5 | 14.40 | 120 | 4.37 | 98.2 | 0.93 |
| Methoxyfenozide | 122 | 4.04 | 109 | 5.57 | 115 | 3.82 |
| Mevinphos | 98.1 | 15.70 | 105 | 1.01 | 100 | 2.61 |
| Monocrotophos | 123 | 5.24 | 107 | 19.40 | 114 | 0.87 |
| Myclobutanil | 111 | 2.67 | 120 | 0.35 | 105 | 1.23 |
| Omethoate | 88.0 | 4.89 | 88.8 | 2.68 | 90.4 | 1.99 |
| Paclobutrazol | 101 | 1.89 | 105 | 5.43 | 112 | 3.48 |
| Penconazole | 106 | 0.98 | 105 | 5.05 | 112 | 5.46 |
| Phenthoate | 106 | 3.72 | 100 | 4.61 | 103 | 1.73 |
| Phorate | 110 | 5.66 | 115 | 9.36 | 106 | 13.00 |
| Phorate sulfone | 103 | 0.65 | 107 | 3.76 | 113 | 3.80 |
| Phosalone | 104 | 4.69 | 105 | 1.30 | 111 | 0.13 |
| Phosfolan | 99.3 | 2.27 | 105 | 3.54 | 108 | 1.57 |
| Phosmet | 117 | 12.50 | 131 | 5.51 | 112 | 2.78 |
| Phosphamidon | 100 | 10.80 | 104 | 1.97 | 117 | 3.31 |
| Piperonyl butoxide | 107 | 1.48 | 107 | 3.57 | 109 | 4.08 |
| Pirimicarb | 105 | 6.87 | 105 | 0.86 | 95.3 | 1.84 |
| Pirimiphos-methyl | 115 | 4.82 | 113 | 0.99 | 111 | 0.57 |
| Prochloraz | 105 | 6.72 | 107 | 0.51 | 113 | 2.66 |
| Profenofos | 100 | 7.02 | 123 | 4.79 | 111 | 0.97 |
| Propamocarb | 64.0 | 5.33 | 62.5 | 1.05 | 64.0 | 3.17 |
| Propiconazole | 107 | 2.61 | 105 | 0.44 | 110 | 1.57 |
| Prothiophos | 90.4 | 3.21 | 125 | 12.70 | 104 | 9.58 |
| Pyraclostrobine | 110 | 6.09 | 98.5 | 4.41 | 112 | 3.13 |
| Pyridaben | 105 | 2.02 | 128 | 5.46 | 100 | 3.94 |
| Pyridafenthion | 112 | 4.89 | 119 | 8.41 | 110 | 2.39 |
| Pyrimethanil | 91.8 | 1.67 | 103 | 7.72 | 113 | 4.00 |
| Pyrimitate | 108 | 2.12 | 110 | 4.74 | 112 | 2.01 |
| Pyriproxyfen | 102 | 1.61 | 119 | 1.77 | 111 | 4.22 |
| Pyrisoxazole | 103 | 8.44 | 92.9 | 7.61 | 117 | 4.91 |
| Quizalofop-p-ethyl | 115 | 1.00 | 120 | 1.07 | 111 | 5.87 |
| Simeconazole | 109 | 7.30 | 125 | 6.56 | 100 | 9.67 |
| Spirodiclofen | 105 | 7.17 | 107 | 2.58 | 103 | 3.58 |
| Sulfotep | 98.9 | 3.46 | 104 | 11.80 | 104 | 2.82 |
| Sulprofos | 107 | 1.36 | 120 | 6.88 | 105 | 0.72 |
| Tebuconazole | 113 | 2.20 | 112 | 6.75 | 109 | 2.38 |
| Tebufenozide | 118 | 1.09 | 108 | 0.66 | 112 | 3.77 |
| Tebufenpyrad | 121 | 1.71 | 113 | 2.34 | 114 | 1.71 |
| Temephos | 102 | 1.74 | 116 | 1.67 | 115 | 0.52 |
| Terbufos | 116 | 2.13 | 125 | 3.23 | 95.5 | 1.52 |
| Tetrachlorvinphos | 94.3 | 3.72 | 114 | 3.58 | 113 | 2.79 |
| Tetraconazole | 124 | 2.74 | 109 | 5.92 | 111 | 2.26 |
| Thiacloprid | 98.5 | 2.21 | 103 | 3.24 | 98.8 | 1.68 |
| Thiamethoxam | 94.0 | 2.24 | 107 | 3.22 | 96.0 | 4.43 |
| Thiophanate-methyl | 108 | 5.86 | 100 | 1.74 | 112 | 4.96 |
| Tolfenpyrad | 109 | 6.05 | 113 | 2.57 | 111 | 0.60 |
| Triadimefon | 123 | 5.51 | 113 | 0.41 | 109 | 1.60 |
| Triadimenol | 127 | 6.53 | 127 | 2.66 | 113 | 1.07 |
| Tricyclazole | 90.1 | 3.59 | 95.0 | 0.79 | 102 | 0.78 |
| Trifloxystrobin | 115 | 3.61 | 110 | 4.97 | 116 | 4.16 |
| Triticonazole | 103 | 3.11 | 126 | 1.25 | 115 | 5.05 |
| Uniconazole | 124 | 6.68 | 104 | 0.89 | 111 | 1.36 |
| Vitavax | 100 | 0.64 | 95.4 | 3.83 | 109 | 2.35 |

Table S12 Recoveries of the pesticides in the spiked PRA-HB with the different concentrations of 20 μg/kg, 50 μg/kg and 200 μg/kg.

| Spiked concentration | 20 μg/kg | | 50 μg/kg | | 200 μg/kg | |
| --- | --- | --- | --- | --- | --- | --- |
| Pesticides | Recovery (%) | RSD (%) | Recovery (%) | RSD (%) | Recovery (%) | RSD (%) |
| 3-Hydroxy-carbofuran | -- | -- | -- | -- | 123 | 1.10 |
| Abamectin | -- | -- | 91.5 | 10.40 | 124 | 12.20 |
| Acephate | 95.3 | 1.54 | 81.8 | 2.52 | 84.0 | 0.96 |
| Acetamiprid | 107 | 3.99 | 94.7 | 2.41 | 106 | 1.96 |
| Alachlor | 104 | 1.30 | 96.1 | 1.40 | 110 | 0.81 |
| Atrazine | 106 | 8.00 | 101 | 2.51 | 103 | 0.74 |
| Azaconazole | 105 | 4.88 | 101 | 5.71 | 113 | 1.35 |
| Azamethiphos | 115 | 8.77 | 100 | 6.57 | 104 | 5.39 |
| Azoxystrobin | 112 | 1.93 | 100 | 2.76 | 111 | 3.47 |
| Benalaxyl | 108 | 6.53 | 95.5 | 3.83 | 109 | 0.73 |
| Bendiocarb | -- | -- | 91.1 | 7.31 | 112 | 1.35 |
| Bitertanol | 99.2 | 9.06 | 123 | 7.68 | 121 | 3.74 |
| Boscalid | -- | -- | 105 | 6.40 | 105 | 2.50 |
| Bromuconazole | 117 | 2.42 | 102 | 13.00 | 107 | 5.22 |
| Buprofezin | 98.3 | 0.77 | 86.8 | 2.95 | 109 | 2.71 |
| Butachlor | 111 | 2.16 | 100 | 3.46 | 111 | 0.30 |
| Cadusafos | 120 | 1.76 | 101 | 1.81 | 108 | 4.80 |
| Carbendazim | 92.0 | 4.97 | 78.9 | 2.29 | 98.1 | 5.32 |
| Carbofuran | 117 | 8.99 | 98.8 | 3.93 | 103 | 1.84 |
| Chlorantraniliprole | 111 | 14.80 | 102 | 2.77 | 113 | 3.48 |
| Chlorpyrifos | 102 | 2.41 | 96.5 | 0.91 | 107 | 3.82 |
| Chlortoluron | 102 | 3.65 | 91.9 | 2.49 | 96.2 | 1.97 |
| Clothianidin | -- | -- | 86.4 | 2.41 | 91.8 | 0.07 |
| Coumaphos | 110 | 2.79 | 103 | 5.39 | 112 | 2.88 |
| Cyanophenphos | 121 | 6.05 | 110 | 2.22 | 126 | 17.80 |
| Cyazofamid` | -- | -- | 116 | 6.89 | 117 | 10.90 |
| Cyproconazole | 122 | 2.24 | 103 | 5.75 | 117 | 0.32 |
| Cyprodinil | 110 | 7.52 | 93.4 | 3.43 | 111 | 0.84 |
| Dicofol | -- | -- | -- | -- | 104 | 4.96 |
| Dicrotophos | -- | -- | 88.9 | 10.00 | 89.2 | 2.26 |
| Difenoconazole | 109 | 1.37 | 103 | 0.52 | 111 | 2.01 |
| Dimethoate | 103 | 2.55 | 91.6 | 0.35 | 101 | 0.79 |
| Dimethomorph | 118 | 6.22 | 103 | 2.16 | 107 | 2.51 |
| dimethyl phthalate | 97.7 | 1.92 | 100 | 2.11 | 100 | 1.90 |
| Diniconazole | 103 | 6.19 | 106 | 3.14 | 111 | 0.59 |
| Dinotefuran | 94.5 | 2.53 | 89.5 | 0.68 | 92.5 | 2.13 |
| Emamectin B1 Benzoate | 102 | 1.60 | 88.8 | 1.11 | 104 | 0.57 |
| Epoxiconazole | -- | -- | 99.1 | 11.70 | 108 | 10.60 |
| Ethiofencarb | 98.3 | 0.83 | 90.1 | 4.08 | 95.4 | 0.86 |
| Etofenprox | -- | -- | 98.7 | 2.01 | 113 | 2.36 |
| Etoxazole | 110 | 1.90 | 94.0 | 0.17 | 101 | 1.62 |
| Fenamiphos | 120 | 2.52 | 103 | 2.39 | 111 | 1.89 |
| Fenamiphos sulfoxide | 100 | 3.08 | 100 | 1.92 | 114 | 0.45 |
| Fenbuconazole | -- | -- | 102 | 3.50 | 114 | 4.20 |
| Fenpropathrin | 116 | 16.80 | 109 | 7.74 | 132 | 12.30 |
| Fipronil | 110 | 8.37 | 107 | 11.50 | 101 | 0.86 |
| Fipronil-sulfone | 114 | 2.61 | 99.2 | 1.25 | 106 | 2.76 |
| Fludioxonil | 96.9 | 4.22 | 102 | 2.68 | 107 | 1.37 |
| Fluopyram | 126 | 1.27 | 104 | 1.46 | 108 | 2.51 |
| Fluoxastrobin | 121 | 2.42 | 102 | 3.83 | 110 | 7.70 |
| Flusilazole | 120 | 2.06 | 100 | 9.35 | 104 | 0.68 |
| Flutolanil | 111 | 1.74 | 97.6 | 5.28 | 109 | 0.72 |
| Flutriafol | 83.5 | 1.47 | 96.6 | 3.18 | 107 | 1.38 |
| Forchlorfenuron | 109 | 4.42 | 94.7 | 1.37 | 102 | 1.07 |
| Hexaconazole | 117 | 1.78 | 92.4 | 2.05 | 111 | 3.41 |
| Hexaflumuron | 110 | 4.10 | 104 | 3.19 | 118 | 8.19 |
| Hexazinone | 110 | 2.95 | 97.9 | 0.98 | 100 | 1.39 |
| Imazalil | 102 | 0.54 | 105 | 1.23 | 109 | 2.27 |
| Indoxacarb | -- | -- | 84.9 | 8.22 | 114 | 2.03 |
| Ipconazole | 120 | 2.00 | 98.5 | 2.41 | 109 | 0.63 |
| Isoprocarb | 113 | 1.57 | 96.4 | 2.40 | 103 | 4.63 |
| Kresoxim-methyl | -- | -- | 99.2 | 3.04 | 119 | 2.61 |
| Lufenuron | -- | -- | -- | -- | 105 | 0.59 |
| Malaoxon | 103 | 2.19 | 97.4 | 0.85 | 110 | 1.15 |
| Malathion | 107 | 8.13 | 101 | 2.53 | 108 | 1.39 |
| Mefenoxam | 109 | 2.33 | 98.2 | 4.20 | 112 | 1.09 |
| Mepronil | 112 | 4.90 | 108 | 7.65 | 107 | 1.34 |
| Metalaxyl | 110 | 0.61 | 100 | 3.72 | 108 | 2.43 |
| Methidathion | 127 | 9.22 | 90.3 | 4.19 | 100 | 3.87 |
| Methomyl | 95.1 | 2.74 | 83.4 | 5.67 | 103 | 3.19 |
| Methoprene | 78.4 | 14.80 | 96.0 | 4.14 | 103 | 1.20 |
| Methoxyfenozide | 117 | 5.71 | 100 | 1.80 | 114 | 4.75 |
| Mevinphos | 88.4 | 8.20 | 100 | 0.67 | 103 | 0.86 |
| Monocrotophos | 103 | 10.20 | 98.3 | 4.56 | 96.8 | 4.97 |
| Myclobutanil | 112 | 3.74 | 103 | 2.79 | 104 | 0.60 |
| Omethoate | 91.9 | 4.79 | 89.6 | 1.37 | 90.2 | 2.74 |
| Paclobutrazol | 111 | 1.87 | 97.7 | 3.33 | 105 | 4.54 |
| Penconazole | 97.4 | 7.89 | 98.5 | 10.90 | 112 | 1.01 |
| Phenthoate | 125 | 8.37 | 95.5 | 0.84 | 106 | 1.27 |
| Phorate | 110 | 13.90 | 94.5 | 7.45 | 105 | 1.62 |
| Phorate sulfone | 107 | 6.33 | 90.8 | 0.73 | 111 | 0.30 |
| Phosalone | 116 | 0.33 | 111 | 3.16 | 110 | 2.40 |
| Phosfolan | 105 | 0.79 | 95.7 | 5.27 | 101 | 0.80 |
| Phosmet | -- | -- | 112 | 8.79 | 129 | 1.14 |
| Phosphamidon | 107 | 2.56 | 96.0 | 0.45 | 110 | 0.83 |
| Piperonyl butoxide | 117 | 1.79 | 100 | 2.36 | 107 | 2.79 |
| Pirimicarb | 111 | 1.10 | 95.1 | 1.18 | 98.5 | 3.67 |
| Pirimiphos-methyl | 112 | 3.75 | 95.4 | 0.06 | 115 | 2.19 |
| Prochloraz | 105 | 4.31 | 98.5 | 0.48 | 103 | 0.11 |
| Profenofos | 104 | 1.65 | 95.3 | 1.69 | 115 | 3.15 |
| Propamocarb | 71.3 | 4.60 | 56.3 | 0.75 | 67.7 | 1.81 |
| Propiconazole | 112 | 1.68 | 102 | 0.95 | 110 | 0.54 |
| Prothiophos | -- | -- | -- | -- | 109 | 19.50 |
| Pyraclostrobine | 108 | 3.58 | 103 | 6.93 | 108 | 5.77 |
| Pyridaben | 77.3 | 9.59 | 92.1 | 0.67 | 110 | 1.31 |
| Pyridafenthion | 117 | 8.32 | 105 | 3.44 | 108 | 5.92 |
| Pyrimethanil | 112 | 3.99 | 105 | 6.37 | 104 | 2.65 |
| Pyrimitate | 105 | 0.91 | 96.5 | 5.58 | 108 | 4.87 |
| Pyriproxyfen | 107 | 0.68 | 96.8 | 0.91 | 118 | 1.11 |
| Pyrisoxazole | 93.3 | 12.90 | 112 | 9.27 | 103 | 3.29 |
| Quizalofop-p-ethyl | 113 | 4.80 | 106 | 3.62 | 113 | 1.74 |
| Simeconazole | 110 | 3.09 | 108 | 2.35 | 103 | 2.67 |
| Spirodiclofen | 93.3 | 6.33 | 95.2 | 5.47 | 117 | 1.39 |
| Sulfotep | 119 | 9.05 | 102 | 1.93 | 110 | 0.51 |
| Sulprofos | 94.4 | 3.39 | 101 | 1.48 | 104 | 0.91 |
| Tebuconazole | 100 | 1.80 | 96.0 | 2.93 | 109 | 0.90 |
| Tebufenozide | -- | -- | 93.8 | 2.37 | 107 | 2.96 |
| Tebufenpyrad | 105 | 2.16 | 85.9 | 1.53 | 116 | 1.56 |
| Temephos | 103 | 2.11 | 96.7 | 5.36 | 117 | 2.45 |
| Terbufos | 87.5 | 8.27 | 96.5 | 8.36 | 111 | 2.93 |
| Tetrachlorvinphos | 107 | 0.44 | 104 | 4.42 | 107 | 5.28 |
| Tetraconazole | 125 | 5.32 | 108 | 2.21 | 113 | 1.54 |
| Thiacloprid | 102 | 3.80 | 91.4 | 3.07 | 96.9 | 1.58 |
| Thiamethoxam | 104 | 12.20 | 91.0 | 4.98 | 108 | 2.05 |
| Thiophanate-methyl | 105 | 3.41 | 96.7 | 0.75 | 106 | 1.37 |
| Tolfenpyrad | 108 | 0.54 | 98.8 | 2.52 | 115 | 0.69 |
| Triadimefon | 109 | 1.20 | 101 | 6.03 | 105 | 3.71 |
| Triadimenol | 122 | 13.60 | 89.3 | 2.61 | 106 | 4.61 |
| Tricyclazole | 97.4 | 2.31 | 89.3 | 5.83 | 103 | 3.27 |
| Trifloxystrobin | 111 | 0.34 | 102 | 3.67 | 108 | 2.40 |
| Triticonazole | 112 | 0.77 | 111 | 6.50 | 115 | 7.67 |
| Uniconazole | 123 | 6.53 | 100 | 3.60 | 113 | 4.60 |
| Vitavax | 104 | 3.75 | 93.5 | 4.13 | 109 | 0.42 |
